# Tumor treating fields affect mesothelioma cell proliferation by exerting histotype-dependent cell cycle checkpoint activations and transcriptional modulations

**DOI:** 10.1038/s41419-022-05073-4

**Published:** 2022-07-15

**Authors:** Laura Mannarino, Federica Mirimao, Nicolò Panini, Lara Paracchini, Sergio Marchini, Luca Beltrame, Rosy Amodeo, Federica Grosso, Roberta Libener, Irene De Simone, Giovanni L. Ceresoli, Paolo A. Zucali, Monica Lupi, Maurizio D’Incalci

**Affiliations:** 1 Laboratory of Cancer Pharmacology, IRCCS Humanitas Research Hospital, Rozzano, Milano, Italy; 2 Department of Biomedical Sciences, Humanitas University, Pieve Emanuele, Milano, Italy; 3 Department of Oncology, Istituto di Ricerche Farmacologiche Mario Negri IRCCS, Milano, Italy; 4 Oncology Division, Azienda Ospedaliera SS Antonio e Biagio e Cesare Arrigo, Alessandria, Italy; 5 Department of Integrated Activities Research and Innovation, Azienda Ospedaliera SS Antonio e Biagio e Cesare Arrigo, Alessandria, Italy; 6 Medical Oncology Unit, Saronno Hospital, ASST Valle Olona, Saronno, Varese, Italy; 7 Department of Oncology, IRCCS Humanitas Research Hospital, Rozzano, Milano, Italy

**Keywords:** Mesothelioma, Cancer therapy

## Abstract

Although clinical antitumor activity of Tumor Treating Fields (TTFields) has been reported in malignant pleural mesothelioma (MPM) patients, the mechanisms behind the different selectivity displayed by the various MPM histotypes to this physical therapy has not been elucidated yet. Taking advantage of the development of well characterized human MPM cell lines derived from pleural effusion and/or lavages of patients’ thoracic cavity, we investigated the biological effects of TTFields against these cells, representative of epithelioid, biphasic, and sarcomatoid histotypes. Growth inhibition and cell cycle perturbations caused by TTFields were investigated side by side with RNA-Seq analyses at different exposure times to identify pathways involved in cell response to treatment. We observed significant differences of response to TTFields among the cell lines. Cell cycle analysis revealed that the most sensitive cells (epithelioid CD473) were blocked in G_2_M phase followed by formation of polyploid cells. The least sensitive cells (sarcomatoid CD60) were only slightly affected by TTFields with a general delay in all cell cycle phases. Apoptosis was present in all samples, but while epithelioid cell death was already observed during the first 24 h of treatment, sarcomatoid cells needed longer times before they engaged apoptotic pathways. RNA-Seq experiments demonstrated that TTFields induced a transcriptional response already detectable at early time points (8 h). The number of differentially expressed genes was higher in CD473 than in CD60 cells, involving several pathways, such as those pertinent to cell cycle checkpoints, DNA repair, and histone modifications. Our data provide further support to the notion that the antitumor effects of TTFields are not simply related to a non-specific reaction to a physical stimulus, but are dependent on the biological background of the cells and the particular sensitivity to TTFields observed in epithelioid MPM cells is associated with a higher transcriptional activity than that observed in sarcomatoid models.

## Introduction

Malignant pleural mesothelioma (MPM) is an aggressive malignancy with a 5-year survival rate of less than 10% [1]. Despite the increasing knowledge of the biology of this tumor, patients with MPM have a deplorably poor prognosis, with an estimated median survival of only 12–16 months [2], depending on tumor stage and histological subtype. Patients with sarcomatoid MPM have particularly poor outcomes compared to patients with epithelioid histology [3, 4].

Asbestos exposure represents the main cause for the development of this tumor, and its use has been banned or strictly regulated in many countries for several decades. The latency period of 30 to 50 years between asbestos exposure and mesothelioma development means that mortality rates for this neoplasia are still increasing worldwide [5].

The use of surgery in patients with this disease is very limited and still controversial, and most patients are candidates for medical therapy only. Chemotherapy with the combination of platinum compounds with pemetrexed has been the standard of care for many years. The development of novel drug regimens for MPM treatment has been limited during the last two decades, with the exception of combinations including immune checkpoint inhibitors [6]. Recently, the combination of nivolumab and ipilimumab has shown a survival improvement as compared to standard chemotherapy, although the benefit seems limited to patient subgroups (mainly patients with non-epithelioid histology) [7]. In any case, mesothelioma remains a cancer lacking effective therapy options when tumors progress [8]. Therefore, it seems important and timely to search for new and efficacious therapeutic strategies.

A recent phase II clinical trial in patients treated with TTFields plus pemetrexed and either cisplatin or carboplatin has shown encouraging results (median overall survival (OS) of 18.2 months and median progression free survival (PFS) of 7.6 months), without serious adverse effects [9]. These results contributed to the approval by the FDA of TTFields in combination with pemetrexed plus platinum-based chemotherapy for the first-line treatment of unresectable, locally advanced or metastatic, MPM.

TTFields are low intensity, intermediate frequency, alternating electric fields delivered through arrays placed around the anatomic region of the tumor. They are able to disrupt cell division, and their antimitotic effect has been demonstrated in vitro in different tumor types [10]. Recent studies have demonstrated that, apart from their antimitotic effect, TTFields also affect cell motility, cell membrane permeability, and DNA repair; furthermore, they stimulate autophagy and immunogenic cell death (reviewed in [11]).

Clinical and preclinical studies aimed to understand the impact of TTFields administered with chemotherapy to MPM are in progress. Recently, the application of 150 kHz TTFields to two non-epithelioid MPM cell lines was found to increase the formation of DNA double strand breaks, induce the expression of cell cycle inhibitors, and reduce the levels of Fanconi Anemia-BRCA DNA repair pathway proteins. All these effects can conceivably contribute to the increased activity of cisplatin and pemetrexed observed both in in vitro and in vivo tumor models when administered together with TTFields [12]. Nevertheless, there are still gaps in our understanding of the antineoplastic mechanisms engaged by TTFields in MPM tumors. The availability of patient-derived mesothelioma cell lines represents an important tool for studying the response of this tumor to new therapies. We wished to address hitherto unresolved clinically pertinent issues, such as which factors influence MPM susceptibility towards TTFields in the different MPM subtypes.

## Results

### Cell lines characterization and sensitivity to TTFields

**Fig. 1:**
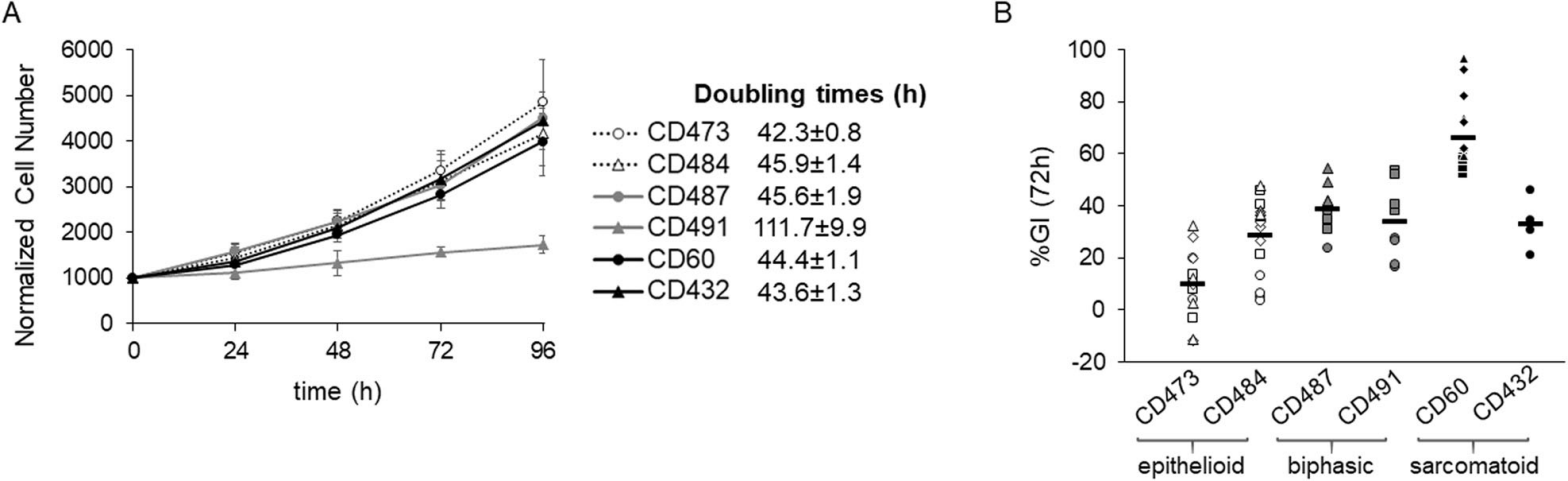
Cell growth in control conditions and TTTFields antiproliferative effect. **A** Growth curves of MPM cell lines in normal culture conditions. Cell growth was monitored by counting the cells with a Coulter Counter every 24 h for 4 days. Each symbol represents the mean of at least four technical replicates of two independent experiments with its standard deviations. Calculated doubling times are shown by the respective cell line. **B** Growth inhibitory effects at 72 h of treatment (%GI (72 h)), calculated as the ratio of the growth rate of treated to control samples. Each symbol represents one replicate, while different symbols in the same data set are used to identify independent biological repeated experiments. Black lines represent the mean value of each data set.

Before exposing the six patient-derived MPM cells to TTFields, cell proliferation was studied in unperturbed conditions. Cells in exponential growth were counted every 24 h for 4 days in order to calculate their doubling times. All the cell lines, except for the biphasic CD491, presented similar doubling times of about 48 h (Fig. 1A).

Sensitivity to TTFields was tested by exposing the cells to continuous treatment at 150 kHz frequency and 1.12 V/cm intensity, conditions similar to those used for the treatment of patients with MPM [9]. At 72 h cell proliferation was inhibited in all cell lines, while MPM epithelioid cells were the most sensitive to TTFields treatment (Fig. 1B). In order to investigate the reasons for the different growth response, we performed further experiments in the cells which were the most and the least sensitive to TTFields, epithelioid CD473, and sarcomatoid CD60, respectively.

The characterization of the genomes of epithelioid CD473 and sarcomatoid CD60 cells demonstrated that the two cell lines have a comparable mutation burden (Supplementary Fig. S1), with 3837 and 3931 synonymous variants, respectively. The number of high confidence genes affected by Somatic Copy Number Alterations (SCNAs) is comparable (2037 and 2684, respectively). The genomic context of the genes affected by SCNAs suggests that these cell lines had a relatively stable genome, with localized regions mainly affected by losses while most of the chromosomes remained neutral (Supplementary Fig. S2).

Among mutated genes, only 13 were affected with somatic variants with a likely pathogenic impact and two (*HERC2* and *ESR1*) were mutated in both cell lines (Supplementary Fig. S1). Epithelioid CD473 cells presented a higher number of variants and some of them are known to be involved in cell proliferation and DNA damage repair (*BAP1* [13], *PMS2* [14], *CDK12* [15], and *ASPM* [16]).

### Antiproliferative effects of TTFields

Both CD473 and CD60 cells were subjected to absolute cell count and flow cytometric analysis of DNA content at several times of exposure to different TTFields intensities, one in common for both cell lines and another higher for the most resistant CD60 cells or lower for the most sensitive CD473 cells.

**Fig. 2:**
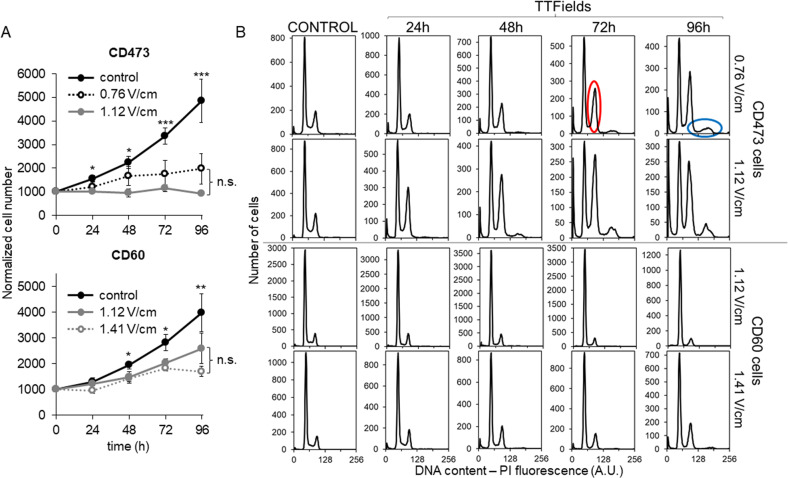
Intensity-dependence of the antiproliferative effects of TTFields. **A** Growth curves of epithelioid CD473 and sarcomatoid CD60 cells during TTFields treatment at two different intensities or under control conditions. Each symbol represents the mean cell number of four replicates in at least two biological independent experiments with its standard deviation. Statistical significance was analyzed by one-way Anova test (*p < 0.05; **p < 0.01; ***p < 0.001). The differences observed between the two treated samples were not statistically significant (n.s.). **B** Representative DNA histograms of epithelioid CD473 and sarcomatoid CD60 controls and treated samples are shown. Examples of polyploid or G_2_M blocked cells are outlined in blue and in red, respectively. Fluorescence signals are reported as arbitrary units (A.U.).

As shown in Fig. 2A, the antiproliferative effects of TTFields are dependent on the intensity of the treatment. Epithelioid cells exposed to 0.76 V/cm TTFields were slowed down in their progression through the cell cycle and a portion of them was intercepted at the G_2_M checkpoint and remained blocked in this phase (Fig. 2B). This portion increased with time and after 48 h some of the cells overcame the block, re-starting DNA replication, yet without subsequent cytokinesis, as demonstrated by the presence of an increasing amount of polyploid cells. As expected, TTFields applied at higher intensity (1.12 V/cm) resulted in lower CD473 cell number and more cells blocked in G_2_M (especially after 48 h of treatment) (Fig. 2B – upper panel, Supplementary Fig. S3).

CD60 cells were also susceptible to the intensity-dependent cell cycle effects of TTFields, but the growth inhibition was not associated to a major cell cycle perturbation, thus suggesting a delay in the progression of cells in all cell cycle phases. Only after 72 h of treatment some of the cells were mildly blocked in G_1_ phase (Fig. 2B – lower panel, Supplementary Fig. S3). Long exposure of CD60 cells to 1.41 V/cm intensity was able to activate the G_2_M checkpoint, reflected by an increasing number of cells remaining blocked in this phase after 96 h of treatment.

**Fig. 3:**
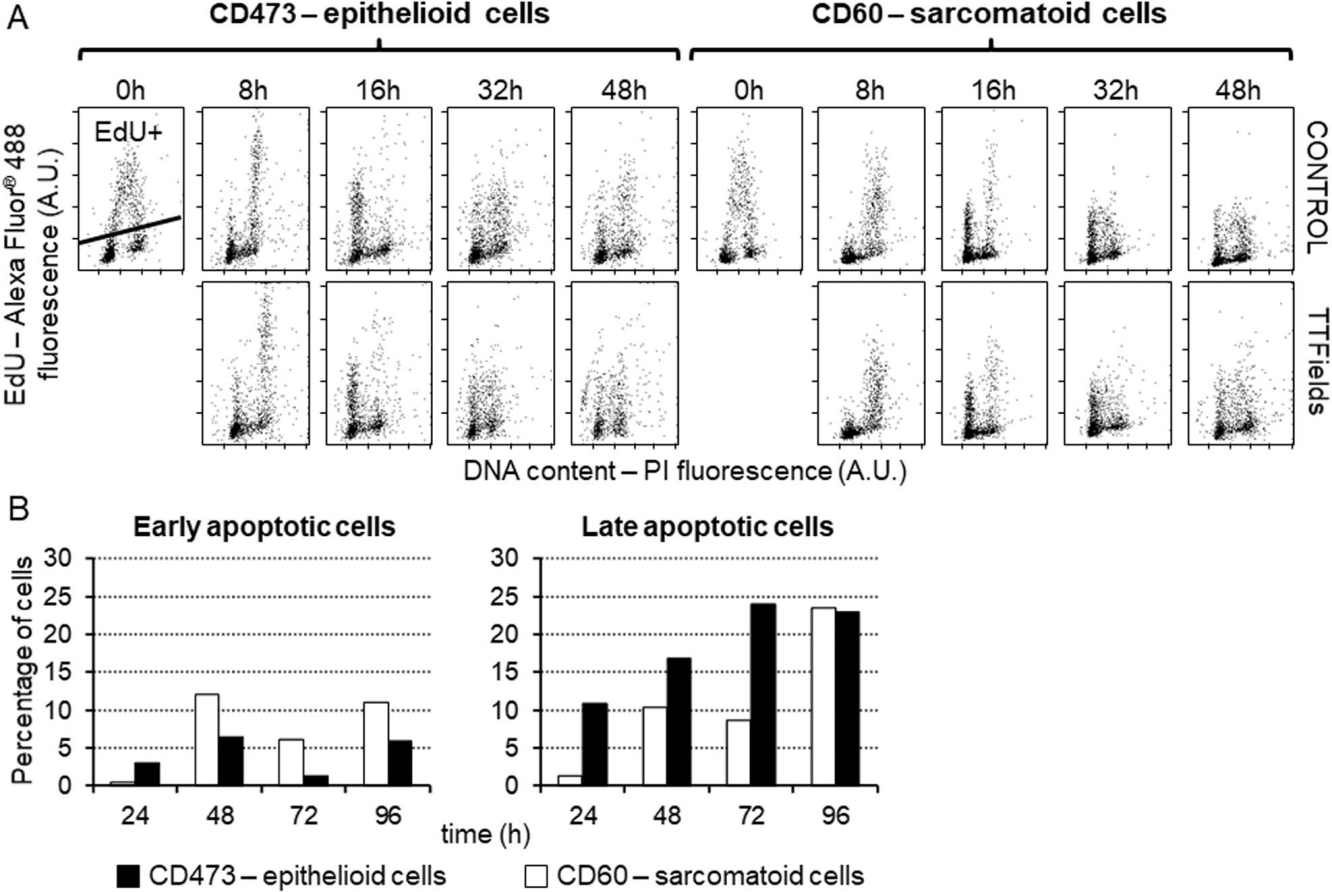
Short-term cell cycle effects of TTFields and apoptosis. **A** Biparametric dot plots of EdU incorporation vs DNA content of epithelioid CD473 and sarcomatoid CD60 cells at different times of exposure to 1.12 V/cm TTFields. Cells detected above the line are EdU-positive (EdU+). **B** Percentage of apoptotic cells obtained by flow cytometric analysis of biparametric staining with ANNEXIN V and PI of epithelioid CD473 and sarcomatoid CD60 cells at different times of exposure to 1.12 V/cm TTFields.

To further investigate cell cycle perturbations induced by TTFields at 1.12 V/cm at short times of treatment, flow cytometric analysis of 5-ethynyl-2′-deoxyuridine (EdU) pulse-chase was performed. Cells were incubated for 1 h with EdU, so that cells in S phase became EdU-positive allowing us to follow their fate during 48 h of TTFields exposure. Both cell types exposed to TTFields proliferated as under control conditions (Fig. 3A). Being the doubling times of both cell lines about 48 h, we might suppose that even in the most sensitive models (epithelioid CD473) almost all the cells were able to conclude at least one cycle during the time of treatment, and that impairments in cell cycle progression might occur after the first cell division. This behavior further corroborates the schedule selected for clinical treatment, where patients undergo a prolonged exposure to TTFields. In contrast, analysis of cell death dynamics performed by Annexin V-PI staining at several times of treatment detected differences in behavior between the two cell types. Apoptosis was observed already at early time points (24 h) in the epithelioid cells whilst only at later time points (from 48 h) in the sarcomatoid cells (Fig. 3B, Supplementary Fig. S4). This was in agreement with the results obtained by western blot analysis of caspase-9 and −3, where increasing levels of caspase-9 were observed only in epithelioid cells (Supplementary Fig. S5).

Western blot analysis of cell cycle checkpoint related proteins (Supplementary Fig. S5) confirmed the main effects observed by flow cytometric assays. The presence of a block in G_1_ phase was associated with an increase of p27 expression in both cell lines and the increase of Chk1 levels observed in CD473 cells matched with the high amount of cells blocked in G_2_M. The different timing observed in the accumulation of p53, starting from 24 h in CD473 cells and from 48 h in CD60 cells, may reflect the delayed activation of cell death and blocking activities observed in the sarcomatoid cells.

**Fig. 4:**
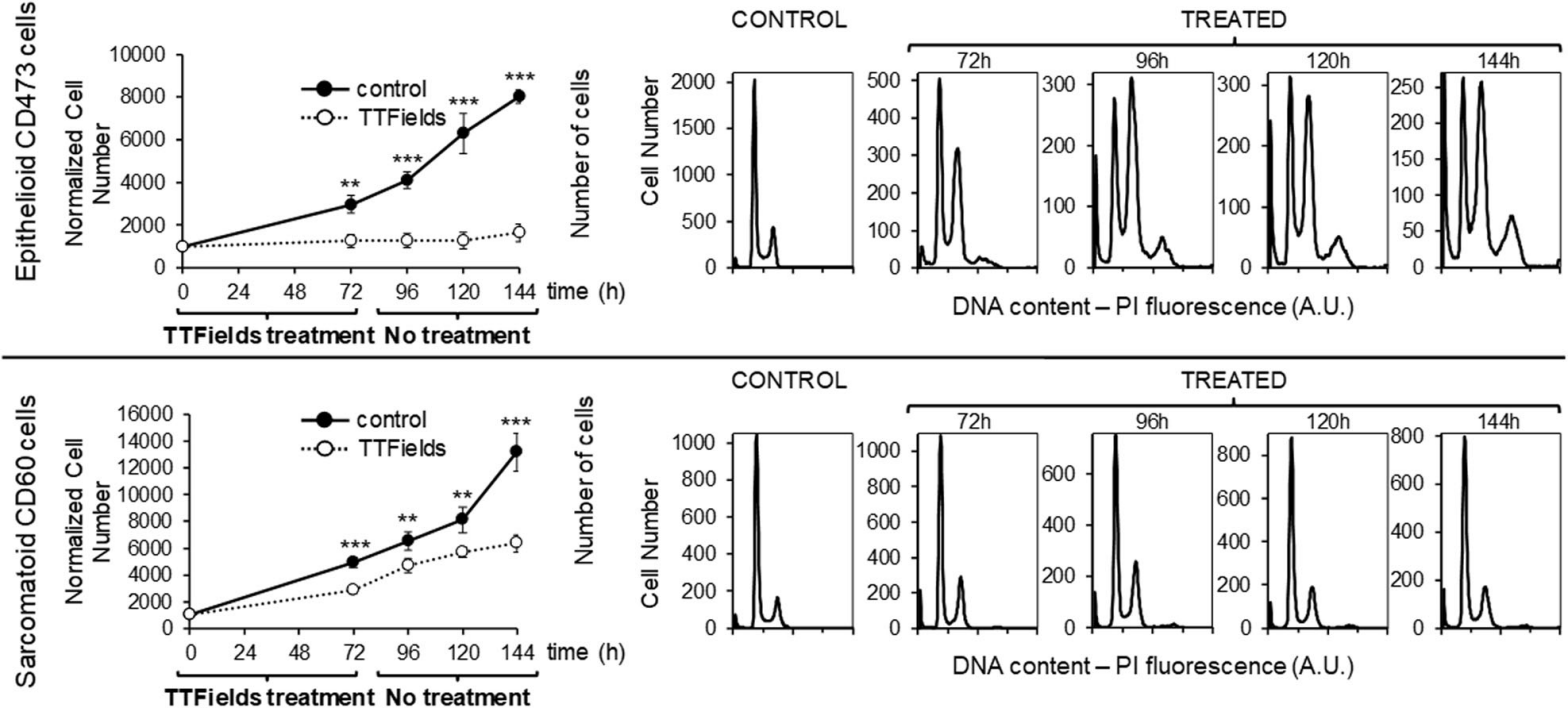
Cell proliferation after TTFields discontinuation. Cell count and DNA histograms of epithelioid CD473 and sarcomatoid CD60 MPM cells exposed for 72 h to 1.12 V/cm TTFields or left untreated, and then let grow under control conditions for an additional 72 h. Statistical significance was analyzed by Student’s *t* test (**p < 0.01; ***p < 0.001).

Sarcomatoid CD60 and epithelioid CD473 cells continued to behave differently also after TTFields discontinuation. When, after 72 h of treatment at 1.12 V/cm intensity, the cells were grown in normal conditions, the epithelioid cells did not re-start growth, whereas the sarcomatoid cells re-commenced cycling, although their growth was slower than that observed in the controls (Fig. 4). The increasing amount of polyploid cells observed in CD473 cultures after TTFields treatment suggests that the antiproliferative effect might be ascribed not only to the physical interaction between cells and electric fields, but also to the induction of permanent or semi-permanent transcriptional modifications or DNA damage permitting escape from cell division even after the end of treatment.

Interestingly, the impaired proliferation of biphasic MPM cells CD487 exposed to 1.12 V/cm was associated with effects on DNA distributions similar to those observed in sarcomatoid CD60 cells, without any accumulation in particular cell cycle phases (Supplementary Fig. S6A). On the other side, cell death replicated the dynamics observed in the epithelioid subtype with about 10% of apoptotic cells already detectable after 24 h of treatment (Supplementary Fig. S6B). After TTFields discontinuation, CD487 cells behave similarly to sarcomatoid cells with an indication of recovery of cell growth at the end of treatment (Supplementary Fig. S6C).

### Transcriptional profiles and modifications

In order to investigate any changes induced by TTFields at the transcriptional level, CD473 and CD60 cells were subjected to transcriptomic analysis under basal conditions and after different times of exposure to 1.12 V/cm TTFields.

**Fig. 5:**
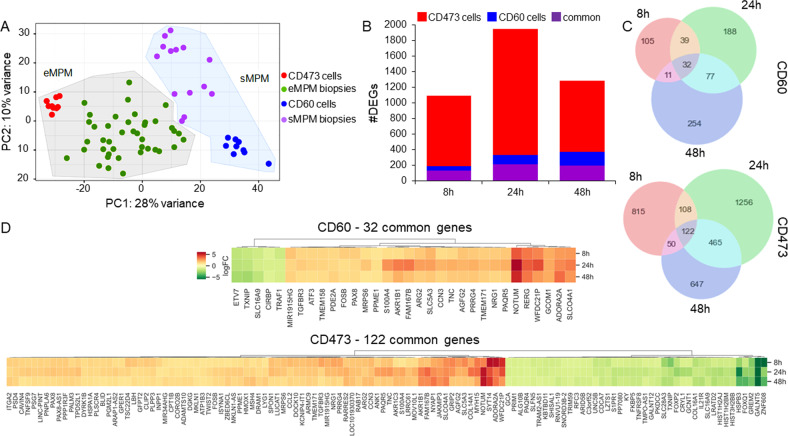
Transcriptomic analysis of control and treated samples. **A** Principal component analysis of the transcriptional profiles of control CD60 and CD473 cells and MPM patient biopsies (eMPM epithelioid MPM, sMPM sarcomatoid MPM, PC principal component). **B** Number (#) of DEGs. **C** Venn diagrams of DEGs. **D** Fold-changes of the common regulated genes in epithelioid CD473 and sarcomatoid CD60 cells at 8, 24 and 48 h of 1.12 V/cm TTFields treatment.

RNA-Seq analysis of control cells showed that their transcriptional profiles were comparable with those derived from 69 patient biopsies of the respective histotype (Fig. 5A). This result confirmed that these cells faithfully reproduce the molecular features of patients’ tumors, constituting a good model for in vitro and future in vivo studies of MPM. RNA-Seq analyses of both cell lines showed that TTFields induced a transcriptional response detectable already at an early time point of treatment (8 h) (Fig. 5B). The overall number of differentially expressed genes (DEGs) was higher in CD473 than in CD60 cells, with a maximal effect at 24 h in CD473 (1951 DEGs) and at 48 h in CD60 (374 DEGs). Some of them were exclusively modulated in the epithelioid or in the sarcomatoid subtype, whereas 128, 211, and 197 were in common to both cell lines at 8 h, 24 h, and 48 h, respectively. The result is consistent with the observed differential sensitivity of the cells towards TTFields, with CD473 more strongly affected than CD60.

In order to identify genes with a selective sensitivity to TTFields modulation, we focused on those with the same transcriptional regulation at all times and we found 32 genes related to CD60 cells and 122 genes related to CD473 cells (Fig. 5C, D). Among these, 24 genes had the same modulation also between cell lines, being mostly up-regulated, while only two, namely *SLC16A9* and *TXNIP*, were down-regulated (Supplementary Table S1). Being the palmitoleoyl-protein carboxylesterase *NOTUM* one of the most up-regulated genes at all time-points in both cell lines, we tested whether the transcriptional modulation was effectively present also at the protein level. As demonstrated by western blot analysis, despite the basal expression of NOTUM was very different in the two MPM subtypes, TTFields exposure increased the protein levels in both cell lines (Supplementary Fig. S7).

**Fig. 6:**
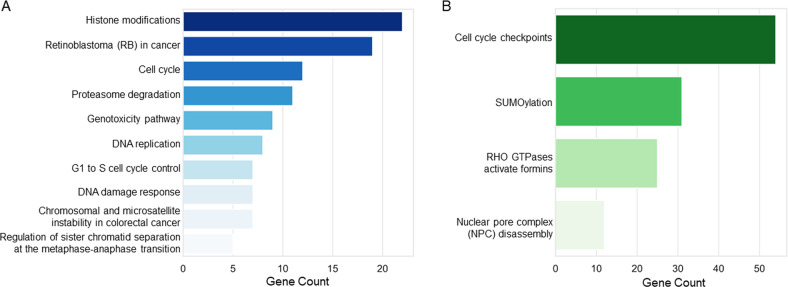
Pathways deregulated by TTFields. **A** Pathways shared by epithelioid and sarcomatoid cells. **B** Pathways exclusively modulated in the epithelioid CD473 cells.

Pathway analysis of DEGs involved in the response to TTFields allowed us to identify deregulated pathways in both cell types, independently (Supplementary Table S2). Accordingly to a greater transcriptomic modulation occurred in epithelioid CD473 cells, we found in this cell line 96 down-regulated pathways at 24 h that were still down-regulated at 48 h (*N* = 84), while only 38 pathways were associated with DEGs at 24 h in the CD60 cell line and none at 48 h. These lasts were down-regulated also in the epithelioid subtype at the same time point. A set of genes, mainly related to histone modifications, cell cycle, DNA replication and DNA damage response, was part of the core enrichment of many of these pathways (Supplementary Table S2) suggesting a specific effect of TTFields (Fig. 6A and Supplementary Fig. S8).

On the other side, comparing CD473 and CD60 treated samples at 24 h, we identified 1329 DEGs that were exclusively modulated in the epithelioid subtype. These DEGs were associated with pathways mainly related to cell cycle checkpoints, SUMOylation, nuclear pore complex and RHO GTPases (Fig. 6B and Supplementary Table S3), letting suppose that they might be responsible for the enhanced sensitivity of epithelioid cells to TTFields.

## Discussion

TTFields are an innovative and non-invasive treatment modality for cancer patients, and have the potential to be an important weapon against MPM. Their activity in the treatment of MPM has been already demonstrated by the results obtained in the STELLAR study [9]. However, one of the issue still unsolved concerns the identification of those factors that might contribute to MPM susceptibility to TTFields. The shortest OS and PFS observed in non-epithelioid patients treated with TTFields let open the hypothesis that the two subtypes may have different sensitivity to this treatment and the comprehension of the causes that determine this behavior could be helpful in the design of more efficacious therapies. However, the lack of appropriate preclinical models representative of the complexity of the disease is an important limitation to translational research [17].

In this study, we took advantage of the possibility to use cell lines derived from MPM patients which were already known to faithfully reproduce the clinical features of the tumor from the histopathological point of view [18]. Comparing the results obtained by the genomic analysis of epithelioid CD473 and sarcomatoid CD60 cells with previously published genomic characterization of MPM [4, 19], we confirmed that these cellular models are representative of the patient tumor and RNA-Seq data demonstrated that their transcriptional profiles were comparable with those derived from patient biopsies of the respective histotype. All these characteristics contribute to make these cells good models for preclinical studies and, for the first time, we used them to investigate the antiproliferative effects of TTFields.

In spite of dissimilar histology and genetic background, CD473 and CD60 cells present similar proliferative characteristics with comparable doubling times. As in line with TTFields antimitotic properties, a correlation between treatment efficacy and cell doubling time has been previously demonstrated [20], the possibility to work with cells with similar kinetic properties is optimal to avoid misinterpretation of the results coming from TTFields sensitivity studies.

We demonstrated that the effects induced by continuous exposure to TTFields – applied at the same intensity and frequency used in the clinic [9] – are different in the different MPM subtypes, indicating that the cellular response to TTFields is not simply the reaction to a physical stimulus, but is also dependent on the genetic background of the cells [21].

The genomic characterization of the epithelioid CD473 and sarcomatoid CD60 cells has been demonstrated to be helpful in the identification of some genetic alterations that may have a role in the cell response to TTFields. CD473 cells present variants with a likely pathogenic impact in different genes potentially involved in cell proliferation and DNA damage repair pathways and this might represent one of the possible explanation of their sensitivity to TTFields. Further studies employing isogenic MPM cell lines will be useful to elucidate and confirm this hypothesis.

RNA-Seq analysis showed that TTFields cause several transcriptional changes in the epithelioid CD473 and in the sarcomatoid CD60 cells. The fact that some changes related to genes involved in regulatory pathways, such as RHO GTPase, SUMOylation, nuclear pore complex and cytoskeleton, were exclusively or preferentially found in epithelioid CD473 cells suggests that the higher sensitivity of these cells to TTFields could be related to differential transcriptional effects. In particular, the ability of TTFields to affect microtubule organization and to activate Rho-associated coiled-coil kinase (ROCK) signaling pathways has recently been demonstrated also in other cell types, such as glioblastoma cells, identifying it as a novel mechanism by which TTFields may disrupt motility in cancer cells [22].

On the other hand, there are several genes that are similarly modulated in both cell lines, suggesting that some common mechanisms of response to TTFields are unrelated to the growth inhibitory effects. It is attractive to speculate that these transcriptional effects could be exploited to design mechanism-based synergistic combinations. In this respect it is worthwhile underlining that among the genes that were found modulated by TTFields there are some involved in DNA repair and DNA damage response, supporting the rational to combine TTFields with drugs causing DNA damage or radiotherapy, and some recent preclinical and clinical evidences support this hypothesis [12, 23, 24].

An original finding of the present study is that TTFields cause a marked upregulation of *NOTUM* gene. Although the function of *NOTUM* gene is not fully elucidated yet, it has been reported that it is a negative regulator of Wnt signaling [25]. Since the deregulation of Wnt pathway plays an important role not only in the development of many different tumors, but also to the resistance of cancer cells to anticancer drugs [26–28], the upregulation of NOTUM by TTFields might have a therapeutic effect against resistant tumors.

Even though further experiments, both in vitro and in vivo, need to be performed in order to confirm our speculations, the present study show convincingly evidence that TTFields induce specific effects on cell proliferation in different subsets of MPM cells and provide a mechanistic rationale for future therapies and combinations with anticancer drugs, with the possibility to contribute to the design of more effective clinical trials for MPM patients.

## Materials and methods

### Cell lines

MPM cell lines, representative of the three MPM histotypes, were isolated from pleural effusions and/or lavage of patients’ thoracic cavity, before administration of therapeutic treatments with the collaboration of the SS. Antonio e Biagio e Cesare Arrigo Hospital in Alessandria (Italy). All the cell lines were cultured in HAM’s F10 medium (Euroclone, Milan, Italy) supplemented with 10% Fetal Bovine Serum (Euroclone) and 2 mM L-Glutammine (Lonza, Basel, Swiss) [18] and periodically tested for mycoplasma contamination by PCR testing.

MPM cell lines used in this work represent the following subtypes:

CD473 and CD484 MPM cells – epithelioid

CD487 and CD491 MPM cells – biphasic

CD60 and CD432 MPM cells – sarcomatoid

### Treatment

72 h before treatment, 500 µL cell suspension containing about 20000 cells/ml were plated on Thermanox coverslips (Nunc #174977 – ThermoFisher, Waltham, Massachusetts, USA) and put inside the Inovitro™ ceramic dishes (Novocure Ltd, Haifa, Israel) [29]. Once in exponential growth, the cells were treated with TTFields at 150 kHz frequency and 1.12 V/cm intensity for different treatment durations. Different treatment conditions are reported in the figure legends. Control cells were let grow under normal conditions, with no application of TTFields.

### Cell count and flow cytometric analyses

#### Cell count

Cells were harvested and counted with a Coulter Counter MS3 (Beckman Coulter, Brea, California, USA) at different times in normal conditions and during or after TTFields exposure. The normalized cell number was calculated: N(t)*1000/N(0 h), where N(t) is the number obtained from the Coulter Counter at the different time points and N(0 h) is the number of cells at the beginning of treatment. Growth inhibitory effect of TTFields was calculated after 72 h of exposure according to the formula: %GI(72 h) = 100*(N_TTF_(72 h)-N_ctrl_(0 h))/(N_ctrl_(72 h)-N_ctrl_(0 h)), where N_TTF_(72 h) is the number of treated cells at 72 h; N_ctrl_(0 h) and N_ctrl_(72 h) are the numbers of control cells at 0 h and 72 h, respectively.

#### Cell cycle analysis via DNA staining

Cells fixed in 70% ethanol after different times of treatment with TTFields were washed in PBS and incubated for 2 h at room temperature with 25 µg/mL Propidium Iodide (PI) (PromoKine, Heidelberg, Germany) plus 25 µL of 1 mg/mL RNase (Merck, Darmstadt, Germania) in water.

#### Cell proliferation analysis with 5-ethynyl-2′-deoxyuridine (EdU) pulse-chase and DNA staining

Cells were labeled for 1 h with 10 µM EdU (Click-iT® EdU Flow Cytometry Assay Kit #C10425 – Invitrogen, ThermoFisher) and at the end of the incubation they were washed with PBS and put in EdU-free medium. Control and treated samples were harvested at 0, 8, 16, 32, and 48 h, fixed in 70% ethanol and stained following the manufacturer’s instructions.

#### Cell death analysis by ANNEXIN V – PI staining

Cells were stained following the manufacturer’s instructions (eBioscience #BMS500FI-300 – ThermoFisher).

All the samples were analyzed acquiring at least 10000 events with Gallios Flow Cytometer (Beckman Coulter).

### Patient selection

Patients’ biopsies used for transcriptional profiles comparison were collected during the MATCH (Mesothelioma And Translational Clinical and Health) study [30], an observational biological study for the identification of biomolecular markers of MPM, their relationship with survival and tumor response in a retrospective clinical cohort, and their evaluation in a prospective cohort of consecutive patients with newly diagnosed MPM.

The study complied with the Declaration of Helsinki and was conducted per Good Clinical Practice guidelines; it was approved by the Coordinating Ethics Committee “ASO SS. Antonio and Biagio and C. Arrigo” on February, 20th 2019 (committee’s reference number ASO.Meso.19.03) and then by the Ethics Committees of all other six study sites. Written informed consent was obtained from all patients.

### DNA and RNA extraction

DNA and RNA were extracted and purified from cells using an automated extractor (QIACube, QIAGEN, Hilden, Germany) and following the manufacturers’ procedures (QIAmp DNA mini kit #51304 and miRNeasy mini kit #217604, QIAGEN).

DNA and RNA isolated were then quantified using fluorescent assay (BR dsDNA qubit assay #Q32853 and BR RNA qubit assay #Q10211, Life Technologies – ThermoFisher) and their quality was evaluated using Tape station 4200 system (Agilent Technologies, Santa Clara, California, USA).

### Transcriptomic analysis

500 ng of RNA extracted and purified from cell lines was used to prepare total RNA sequencing libraries following the manufacturers’ procedures (TruSeq Stranded Total RNA, Illumina, San Diego, California, USA). Barcoded libraries were then evaluated for quality (Tape Station 4200, Agilent Technologies) and amount (HS dsDNA Qubit assay #Q32854, Life Technologies).

Total RNA stranded libraries were run on a benchtop sequencing system NextSeq500 (Illumina). Raw data were demultiplexed with bcl2fastq (Illumina) and quality assessment was done with FastQC. bcbio-nextgen pipeline (10.5281/zenodo.5781867) was configured for RNA-Seq analysis. Data alignment was done with Hisat2 [31] with the human genome assembly hg38. Gene counts were calculated through pseudo-counts with Salmon [32]. Batch correction was done using ComBat-Seq [33]. Data processing and differential expression analysis were done with DESeq2 [34]. Once assessed that control samples at 8 h, 24 h, and 48 h did not present transcriptional differences in each cell line independently, TTFields treated cells were compared to controls to assess differentially expressed genes (DEGs). Then CD473 and CD60 cells were compared under each condition (basal, 8 h, 24 h, 48 h) to identify differences between cell lines and their response to TTFields. Venn diagrams were done with the matplotlib-venn package (https://github.com/konstantint/matplotlib-venn). Data visualization was done either with embedded functions of DESeq2 or with matplotlib [35]. Heatmaps and clustering were done using *clustermap* from the seaborn package [36] with Ward method. DEGs were associated with biological pathways through the Gene Set Enrichment Analysis (GSEA) [37] with clusterProfiler [38] considering the Reactome database [39] and a corrected p-value (False Discovery Rate, FDR) of 0.01. Since we noticed a redundancy of the same genes in most pathways, we selected these recurrent genes and classified them into pathways using the WikiPathways database [40] with the *enricher* function from clusterProfiler [38]. Genes and pathways were then represented as a network using the Enrichment Map app [41] for Cytoscape [42].

### DNA sequencing

200 ng of purified DNA was enzymatically fragmented and used for a whole-genome amplification. After barcoding procedure, samples were pooled and hybridized with probes covering 6000 clinical-relevant genes (KAPA HyperExome # 09062637001, Roche) and run on NextSeq-500 sequencer system (Illumina).

Raw data were demultiplexed with bcl2fastq (Illumina). bcbio-nextgen pipeline (10.5281/zenodo.5781867) was configured for DNA variant calling analysis. Cell lines were matched to the blood tissue from a mesothelioma patient. Alignment was done through the BWA aligner [43] with the human genome assembly hg38. Somatic variant calling was done with MuTect2 [44] and VarDict [45] considering only variants called by both. Variants were filtered with a 60x coverage and 10% of allelic fraction and annotated with variant effect predictor (VEP) [46]. The Cancer Genome Interpreter (CGI) was used to identify potentially oncogenic variants [47]. Analysis of somatic copy number variants (CNV) was done with CNVkit [48]. Variants and CNV representation was done using CoMut [49]. In order to avoid false positive results due to the use of only one reference for variant calling, genes affected by copy number alterations were selected with stringent conditions using the “genemetrics” option of CNVkit [48] and defined as “high confidence genes”. The copy number profile derived from these genes was plotted using GenVisR [50].

### Western blot analysis

After different times of exposure to TTFields, cells were lysed in RIPA buffer (Sigma Aldrich, Darmstadt, Germany) supplemented with phosphatase and protease inhibitor tablets (PhosSTOP™cOmplete™, EDTA-free Protease Inhibitor Cocktail, Sigma Aldrich) and prepared as described [51]. The primary antibodies used were: anti-NOTUM (#SAB3500082, RRID: AB_10604118; diluted 1:1000, Sigma Aldrich), p27 (F-8) (#sc-1641, RRID: AB_628074; diluted 1:500, Santa Cruz Biotechnology, Dallas, Texas, USA), p53 (DO-1) (#sc-126, RRID: AB_628082; diluted 1:500, Santa Cruz Biotechnology), Chk1 (G4) (#sc-8408, RRID: AB_627257; diluted 1:1000, Santa Cruz Biotechnology), phospho-Chk1 (Ser317) (D12H3) (#12302 S, AB_2783865; diluted 1:1000, Cell Signaling Technology, Danvers, Massachusetts, USA), caspase-3 (#9662 S, RRID: AB_331439; diluted 1:1000, Cell Signaling Technology), caspase-9 (#9502 S, RRID: AB_2068621; diluted 1:1000, Cell Signaling Technology), actin (C-2) (#sc-8432, RRID: AB_626630; diluted 1:500, Santa Cruz Biotechnology) and GAPDH (6C5) (#sc-32233, RRID: AB_627679; diluted 1:1000, Santa Cruz Biotechnology) followed by incubation with an anti-mouse (RRID: AB_11125547) or rabbit (RRID: AB_11125142) secondary antibody horseradish peroxidase (HRP)-conjugated (diluted 1:2500) (Bio-Rad, Hercules, California, USA). Blotted membranes were incubated with Clarity Western ECL substrate or Clarity Max Western ECL substrate (Bio-Rad) and signal detected with ChemiDoc MP imaging system (Bio-Rad). Densitometric analysis of the obtained bands was done with ImageJ software (https://imagej.nih.gov/ij/). Normalized signals to GAPDH were expressed as fold change relative to control.

## Supplementary information

Supplementary Figures

Supplementary Table 1

Supplementary Table 2

Supplementary Table 3

Original Western Blot

Academic Journals Reporting Checklist

Author contribution for

## Data Availability

Sequence data for this study have been deposited in the European Nucleotide Archive (ENA) at EMBL-EBI under accession numbers PRJEB50751 and PRJEB52839. All the other data are available in this published article and its supplementary information files; raw data are available in the open repository Zenodo in the “IRCCS Humanitas Research Hospital & Humanitas University” community (DOI: 10.5281/zenodo.6623710).
