# Supplementary material for: Tumor treating fields affect mesothelioma cell proliferation by exerting histotype-dependent cell cycle checkpoint activations and transcriptional modulations
Source: Cell Death Dis. 2022 Jul 15;13(7):612. doi: 10.1038/s41419-022-05073-4 (PMC9287343; doi:10.1038/s41419-022-05073-4)
Supplement: Supplementary file 1 — Supplementary Figures [file 41419_2022_5073_MOESM1_ESM.pdf]

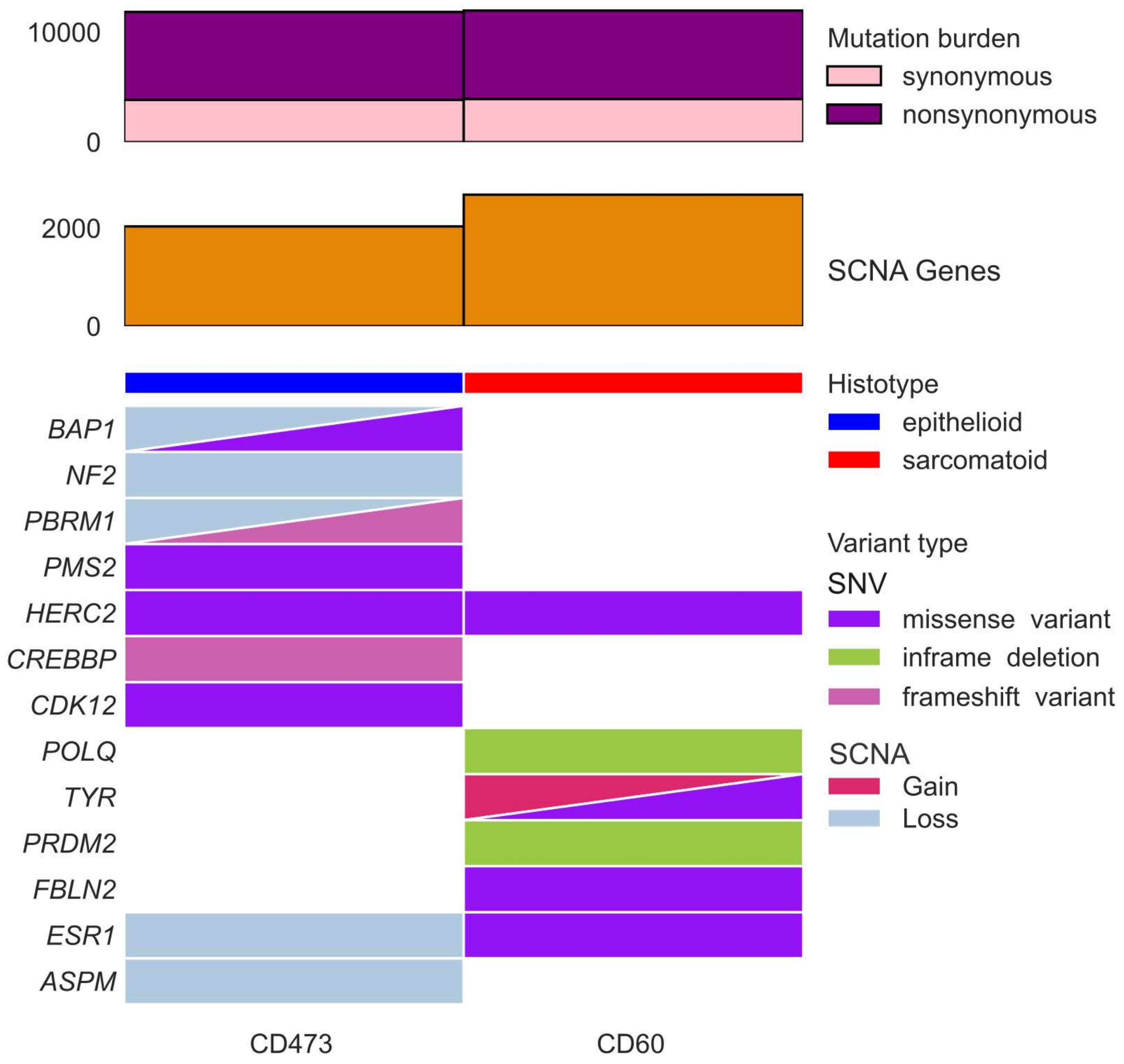

Supplementary Figure S1

Genomic characterization of the epithelioid CD473 and sarcomatoid CD60 cells.

Comparison of the mutation burden and the number of high confidence genes affected by somatic copy number alterations (SCNAs) in CD473 and CD60 cells (upper panels).

List of the genes affected with somatic variants with a likely pathogenic impact (lower panel). The SCNAs of the same genes have been overlapped to somatic nucleotide variants (SNVs).

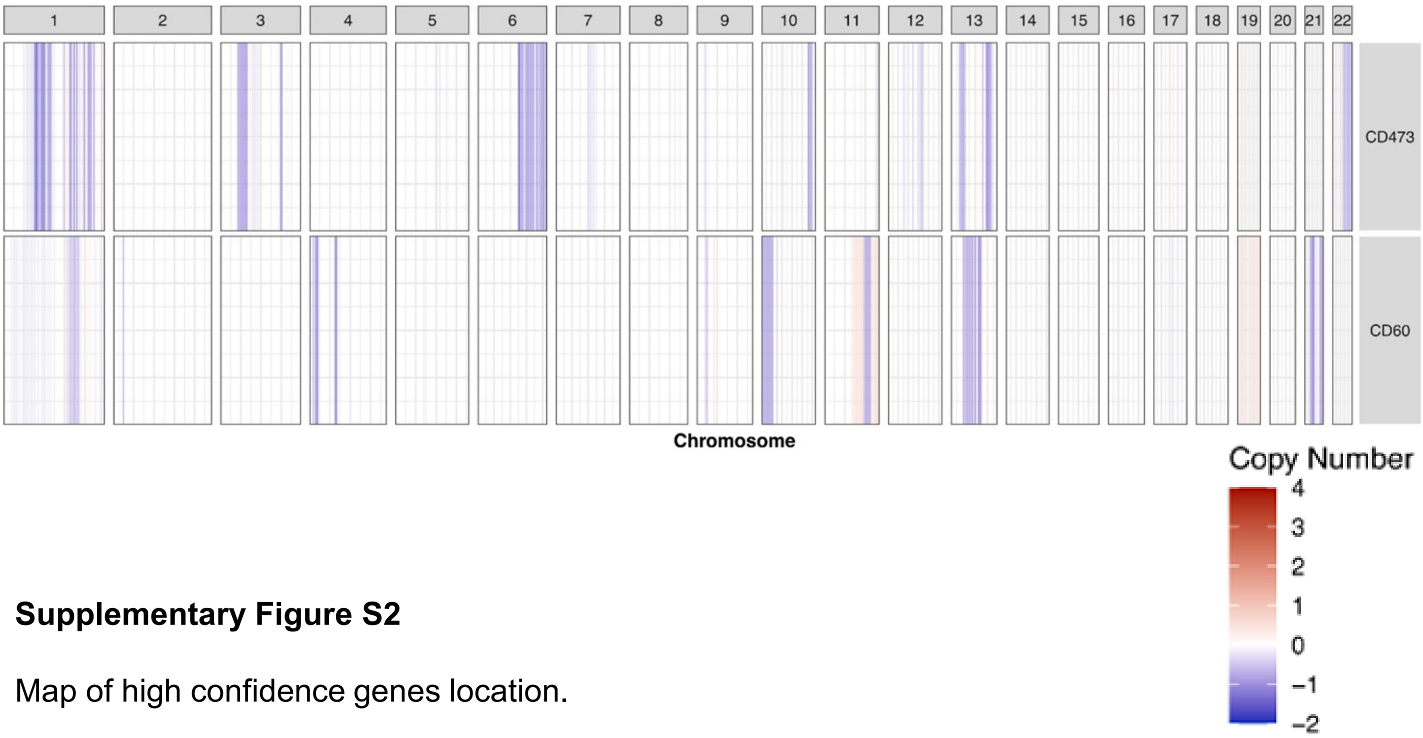

Supplementary Figure S2

Map of high confidence genes location.

Regions of loss (in blue) and gain (in red) in CD473 and CD60 cell lines obtained as reported in Materials and Methods. X-axis reports chromosomes. Colors as in the legend.

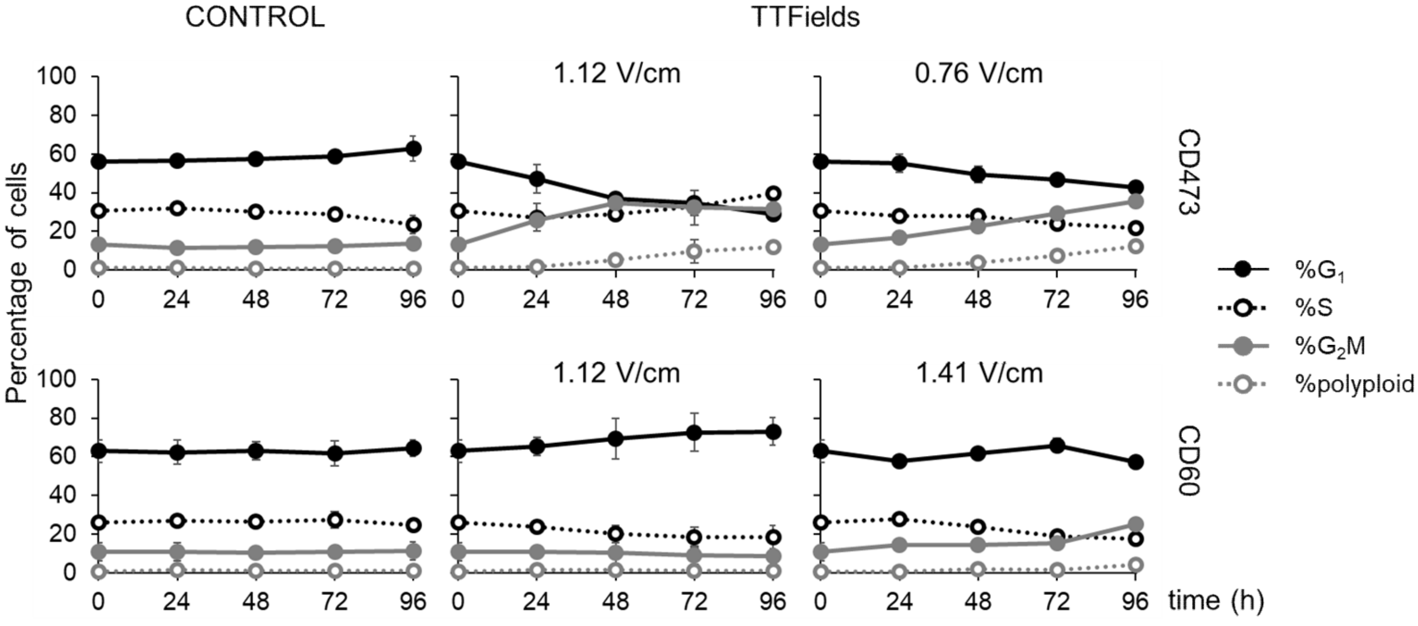

Supplementary Figure S3

Cell cycle effects of TTFields.

Percentage of cells in the different cell cycle phases in control and samples treated with two TTFields intensities. Each symbol represents the mean of at least two independent biological repeated experiments with its standard deviations.

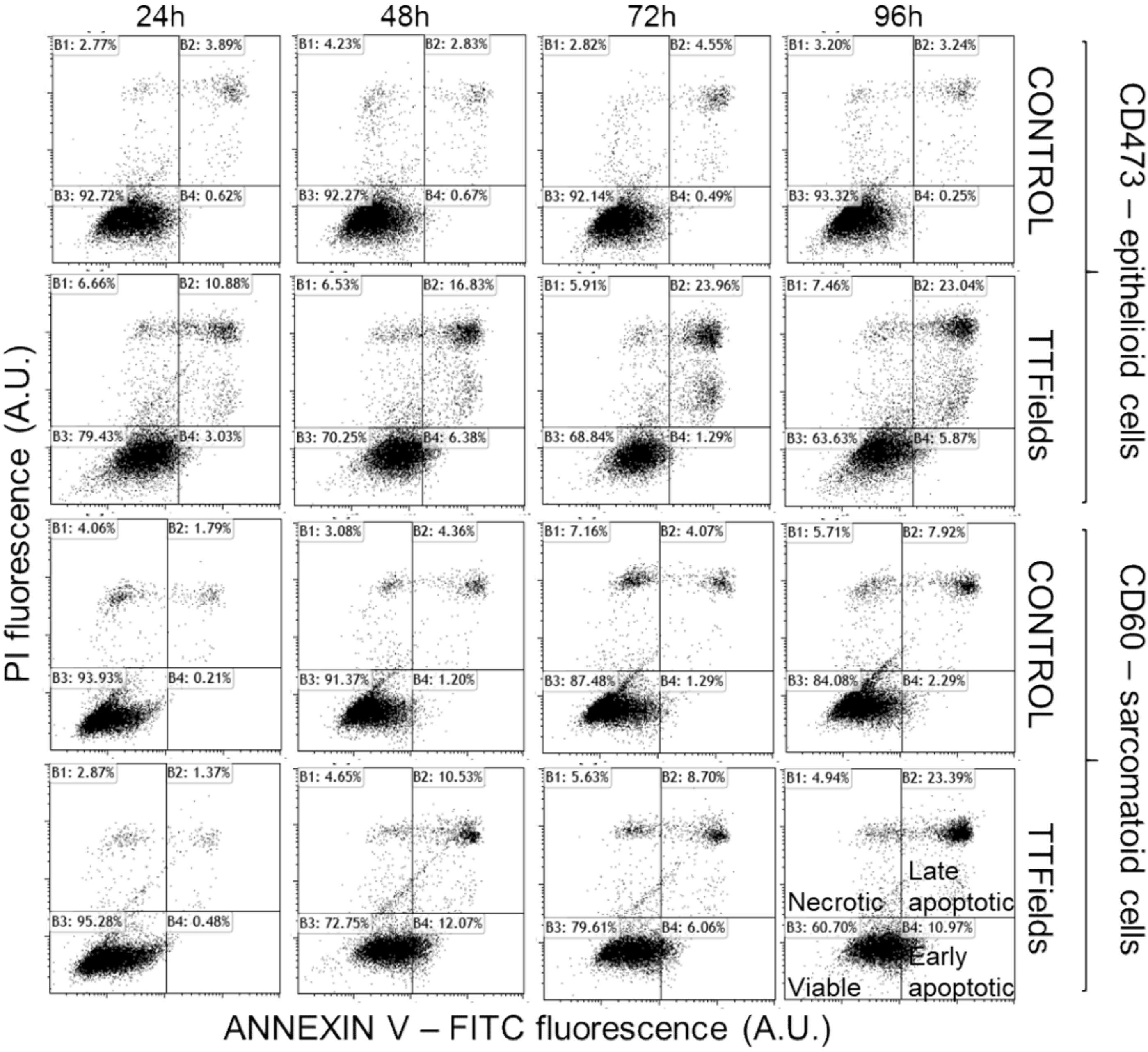

Supplementary Figure S4

Flow cytometric analysis of apoptosis.

Dot plots obtained by flow cytometric analysis of biparametric staining with ANNEXIN V-FITC and PI.

The percentages of viable and apoptotic cells at different times of exposure to TTFields are reported in each plot. At least 10000 cells for each sample were analyzed.

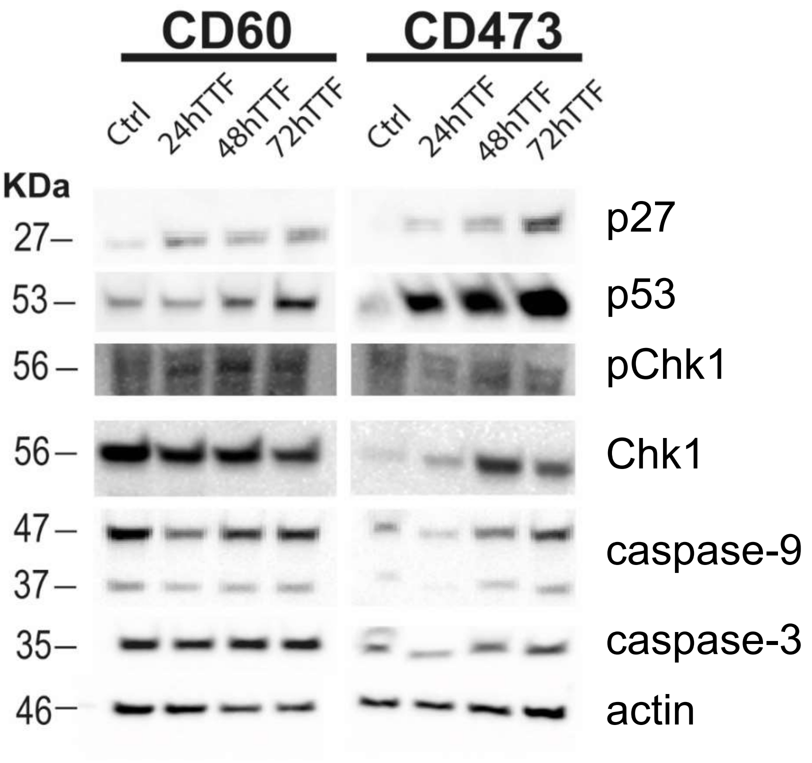

Supplementary Figure S5

Western blot analysis of cell cycle checkpoint related proteins and caspases after TTFields treatment.

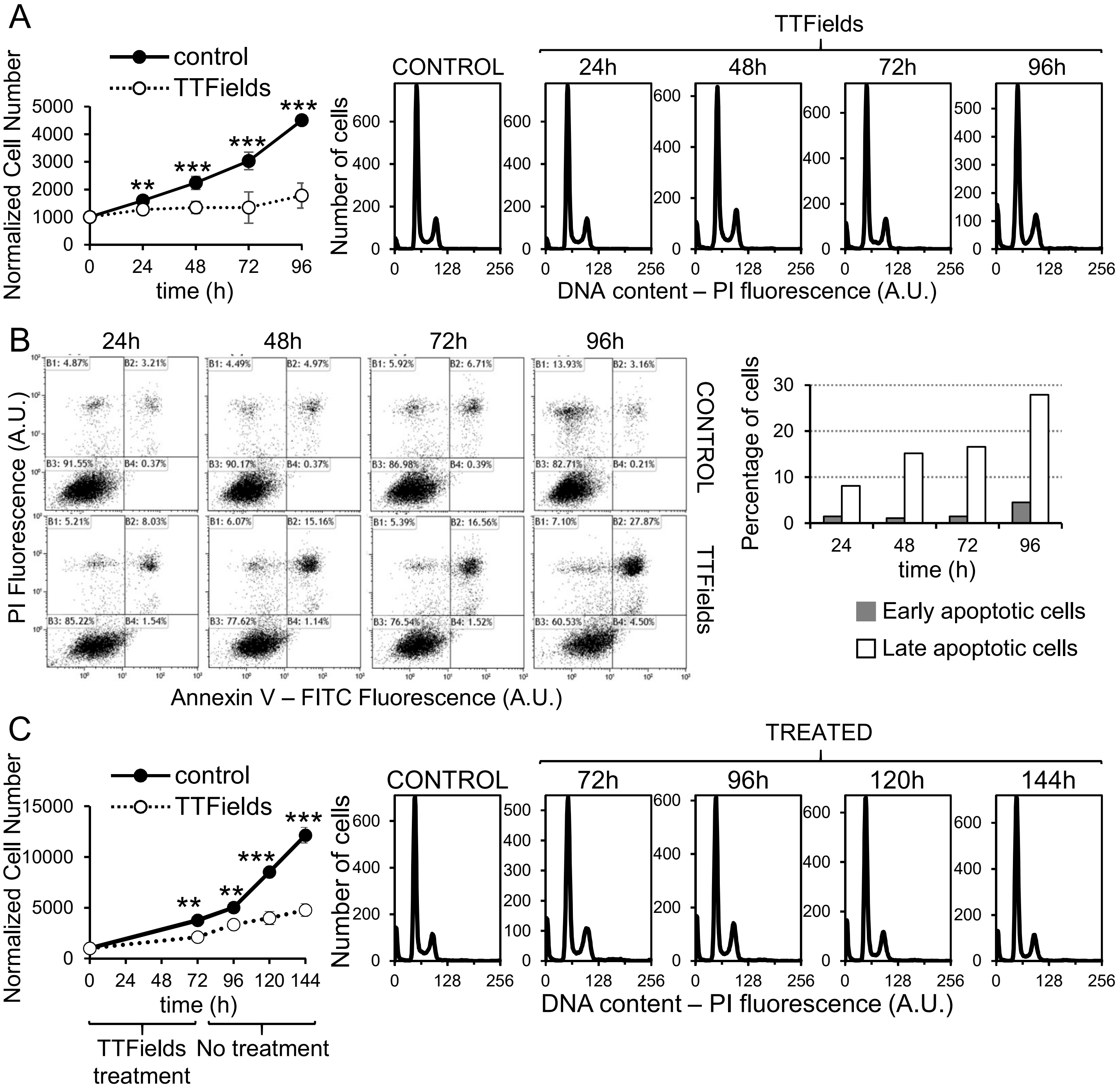

Supplementary Figure S6

Antiproliferative effects of TTFields on biphasic CD487 cells. **A** Cell count and representative DNA histograms of controls and treated samples continuously exposed to 1.12 V/cm TTFields at 150 kHz. **B** Dot plots obtained by flow cytometric analysis of biparametric staining with ANNEXIN V-FITC and PI. The percentages of early and late apoptotic cells in treated samples are compared in the histogram plot. **C** Cell count and representative DNA histograms of CD487 cells exposed for 72h to 1.12 V/cm TTFields or left untreated, that were then grown under control conditions for an additional 72h.

Statistical significance was analyzed by Student's t test (**p<0.01; ***p<0.001).

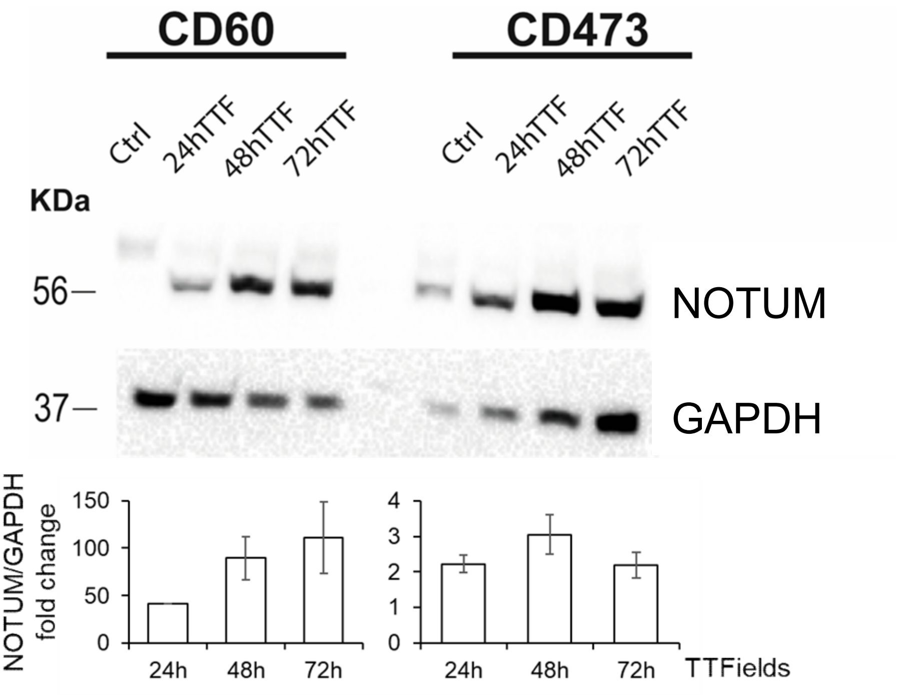

Supplementary Figure S7

Representative western blot of NOTUM expression and densitometric analysis.

Mean fold changes relative to control with their standard deviation were shown.

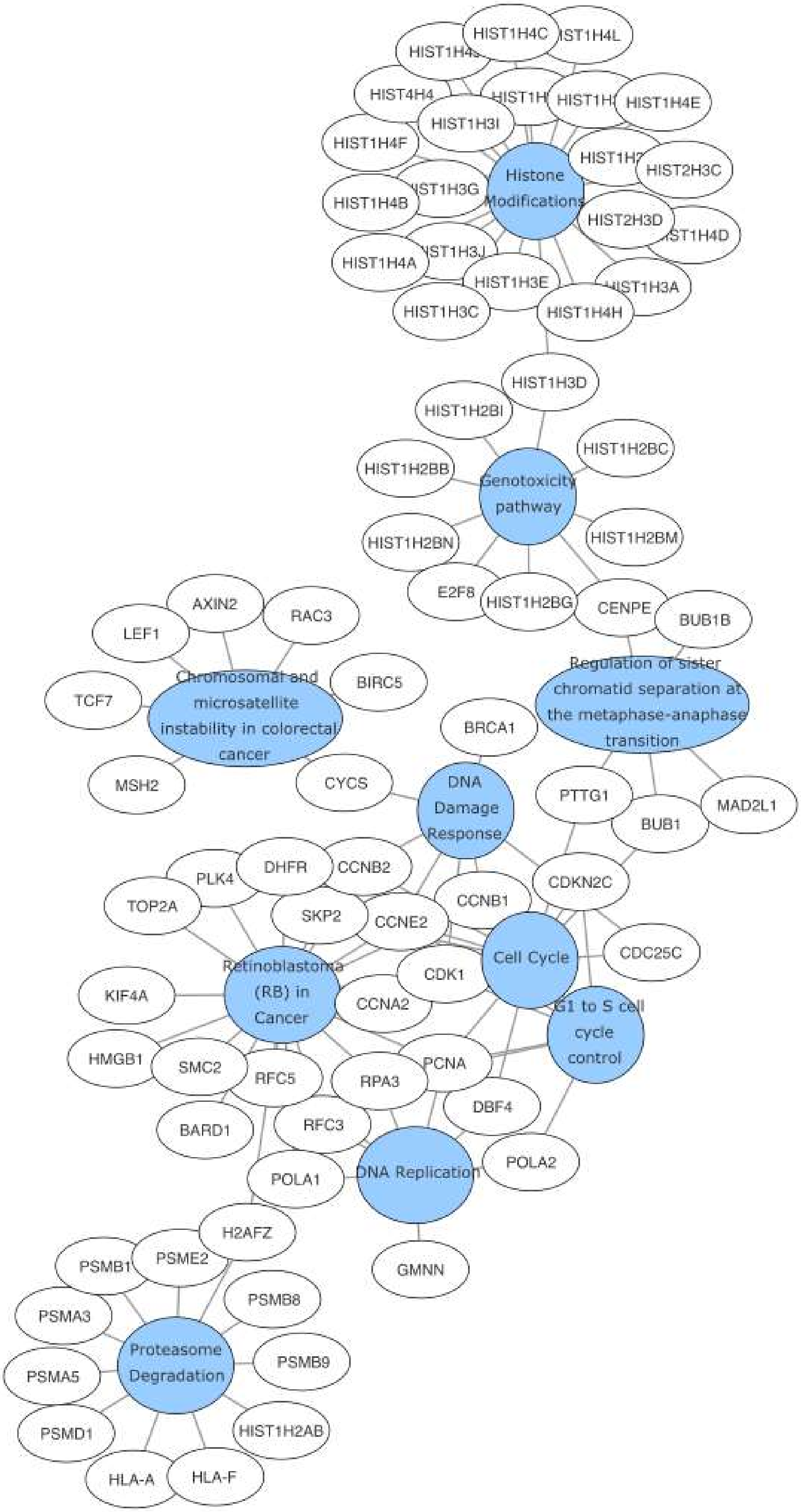

Supplementary Figure S8

Network representation of genes regulated by TTFields treatment.
