# Supplementary material for: Tumor treating fields affect mesothelioma cell proliferation by exerting histotype-dependent cell cycle checkpoint activations and transcriptional modulations
Source: Cell Death Dis. 2022 Jul 15;13(7):612. doi: 10.1038/s41419-022-05073-4 (PMC9287343; doi:10.1038/s41419-022-05073-4)
Supplement: Supplementary file 2 — Supplementary Table 1 [file 41419_2022_5073_MOESM2_ESM.docx]

| **Supplementary TABLE S1: Genes with the same transcriptional regulation at all times of TTFields exposure in both cell lines.** | | | | | | | |
| --- | --- | --- | --- | --- | --- | --- | --- |
| All the genes are listed with their fold changes. | | | | |  |  |  |
|  | **CD60** | | | **CD473** | | |  |
| **symbol** | **8h** | **24h** | **48h** | **8h** | **24h** | **48h** |  |
| **ADORA2A** | 2.739564 | 3.078458 | 3.655935 | 5.513658 | 3.448814 | 3.42647 |  |
| **AGFG2** | 1.863828 | 2.245034 | 1.724236 | 2.732833 | 2.273231 | 1.762884 |  |
| **AKR1B1** | 1.253993 | 3.298 | 2.339236 | 1.92334 | 3.259107 | 3.006638 |  |
| **ARG2** | 1.747615 | 2.670045 | 1.597988 | 1.907797 | 1.606741 | 2.015078 |  |
| **CCN3** | 1.294378 | 2.231708 | 1.467967 | 2.083939 | 1.953208 | 2.035721 |  |
| **FAM167B** | 1.521838 | 3.412189 | 1.966371 | 1.863252 | 3.670184 | 3.033729 |  |
| **FOSB** | 1.594272 | 1.261271 | 1.294872 | 1.081545 | 1.533328 | 1.312754 |  |
| **MIR1915HG** | 1.864835 | 1.44985 | 1.524894 | 2.292088 | 2.089797 | 1.346936 |  |
| **MRPS6** | 1.212085 | 1.786532 | 1.023553 | 1.89604 | 1.790543 | 1.250319 |  |
| **NOTUM** | 4.010149 | 5.828501 | 5.038379 | 3.149887 | 5.450688 | 5.457619 |  |
| **NRG1** | 1.653929 | 2.174335 | 1.44894 | 2.533425 | 2.053197 | 1.46048 |  |
| **PAQR5** | 1.741696 | 2.312736 | 1.515797 | 1.855799 | 2.715426 | 1.752504 |  |
| **PAX8** | 1.639288 | 1.401475 | 1.329339 | 1.604682 | 1.828363 | 1.278417 |  |
| **PPME1** | 1.001771 | 1.218095 | 1.06283 | 1.217851 | 1.667355 | 1.479051 |  |
| **PRRG4** | 1.722362 | 2.33236 | 1.773213 | 2.491521 | 2.162882 | 1.357355 |  |
| **S100A4** | 1.221597 | 2.868532 | 1.595561 | 1.01642 | 2.486486 | 2.040479 |  |
| **SLC16A9** | -1.43339 | -1.2806 | -1.8021 | -1.59796 | -1.91695 | -1.47789 |  |
| **SLC5A3** | 2.176532 | 2.638861 | 1.756224 | 3.065091 | 2.364459 | 1.789036 |  |
| **SLCO4A1** | 2.780166 | 3.300125 | 2.827917 | 2.983184 | 3.019632 | 3.233959 |  |
| **TGFBR3** | 1.446434 | 1.652956 | 1.275469 | 2.558839 | 1.47948 | 1.118124 |  |
| **TMEM171** | 1.634643 | 2.043196 | 1.570466 | 2.025697 | 1.802252 | 1.470095 |  |
| **TNC** | 1.399539 | 2.365217 | 1.721745 | 1.905173 | 2.777594 | 1.645724 |  |
| **TXNIP** | -1.60965 | -1.80645 | -2.42794 | -3.18385 | -1.93108 | -2.05469 |  |
| **WFDC21P** | 3.163506 | 4.264259 | 2.729177 | 4.882719 | 4.23296 | 2.80026 |  |
