# Supplementary material for: Tumor treating fields affect mesothelioma cell proliferation by exerting histotype-dependent cell cycle checkpoint activations and transcriptional modulations
Source: Cell Death Dis. 2022 Jul 15;13(7):612. doi: 10.1038/s41419-022-05073-4 (PMC9287343; doi:10.1038/s41419-022-05073-4)
Supplement: Supplementary file 3 — Supplementary Table 2 [file 41419_2022_5073_MOESM3_ESM.docx]

| **Supplementary Table S2: Deregulated pathways in epithelioid CD473 cells at 24h** | | | | | | | | | |  |  |
| --- | --- | --- | --- | --- | --- | --- | --- | --- | --- | --- | --- |
| **ID** | | **Pathway name** | | **NES** | | | **p.adjust** | **Genes in pathway** | | |  |
| R-HSA-3214858 | | RMTs methylate histone arginines | | -3.625491948 | | | 0.001002937 | HIST1H2AC/HIST4H4/HIST1H3I/HIST1H2AG/HIST2H3C/HIST1H3E/HIST1H4B/HIST2H2AA3/HIST1H4E/HIST2H4A/HIST1H4J/HIST1H4L/HIST1H2AL/HIST1H4C/HIST1H3C/HIST1H4H/HIST2H2AB/HIST1H2AK/HIST1H2AB/HIST1H2AD/HIST1H3G/HIST1H2AM/HIST1H3B/HIST1H3H/HIST1H2AI/HIST1H2AH/HIST1H2AE/HIST1H3A/HIST1H4D/HIST1H3J/HIST1H4A/HIST1H2AJ/HIST2H3D/HIST1H3D/HIST1H3F | | |  |
| R-HSA-5693607 | | Processing of DNA double-strand break ends | | -3.174849077 | | | 0.001002937 | HIST1H2BK/BARD1/DNA2/HIST1H4B/RPA3/CCNA2/HIST1H2BD/HIST1H2BJ/HIST1H2BC/HIST1H2BG/HIST1H4E/HIST2H4A/HIST1H4J/HIST1H4L/HIST1H4C/HIST1H4H/HIST1H2BB/HIST1H2BO/HIST1H2BN/HIST1H2BF/HIST1H2BM/HIST1H2BL/HIST1H2BI/HIST3H2BB/HIST1H4D/HIST1H2BH/HIST1H4A/HIST1H2BE | | |  |
| R-HSA-69473 | | G2/M DNA damage checkpoint | | -3.179273848 | | | 0.001002937 | HIST1H2BK/BARD1/DNA2/HIST1H4B/RPA3/CDC25C/HIST1H2BD/HIST1H2BJ/HIST1H2BC/HIST1H2BG/HIST1H4E/HIST2H4A/HIST1H4J/CDK1/HIST1H4L/HIST1H4C/HIST1H4H/HIST1H2BB/HIST1H2BO/HIST1H2BN/HIST1H2BF/HIST1H2BM/HIST1H2BL/HIST1H2BI/HIST3H2BB/HIST1H4D/HIST1H2BH/HIST1H4A/HIST1H2BE | | |  |
| R-HSA-2559586 | | DNA Damage/Telomere Stress Induced Senescence | | -3.610052868 | | | 0.001002937 | HIST1H2AC/HIST4H4/CCNE2/LMNB1/HIST2H2BE/HIST1H2BK/HIST1H4B/HIST2H2AA3/CCNA2/HIST1H2BD/HIST1H2BJ/HIST1H2BC/HIST1H2BG/HIST1H4E/HIST2H4A/HIST1H4J/HIST1H4L/HIST1H4C/HIST1H4H/HIST1H2AB/HIST1H2BB/HIST1H2AD/HIST1H1D/HIST1H1B/HIST1H2BO/HIST1H2BN/HIST1H2BF/HIST1H2BM/HIST1H2AE/HIST1H2BL/HIST1H2BI/HIST3H2BB/HIST1H4D/HIST1H2BH/HIST1H4A/HIST1H2AJ/HIST1H2BE | | |  |
| R-HSA-73884 | | Base Excision Repair | | -3.142290542 | | | 0.001002937 | HIST1H2BK/PCNA/HIST1H4B/RPA3/HIST2H2AA3/HIST1H2BD/HIST1H2BJ/HIST1H2BC/HIST1H2BG/HIST1H4E/HIST2H4A/HIST1H4J/HIST1H4L/HIST1H4C/HIST1H4H/HIST1H2AB/HIST1H2BB/HIST1H2AD/HIST1H2BO/HIST1H2BN/HIST1H2BF/HIST1H2BM/HIST1H2AE/HIST1H2BL/HIST1H2BI/HIST3H2BB/HIST1H4D/HIST1H2BH/HIST1H4A/HIST1H2AJ/HIST1H2BE | | |  |
| R-HSA-606279 | | Deposition of new CENPA-containing nucleosomes at the centromere | | -3.356522585 | | | 0.001002937 | HIST1H2BK/CENPW/CENPU/HIST1H4B/HIST2H2AA3/HIST1H2BD/HIST1H2BJ/HIST1H2BC/HIST1H2BG/HIST1H4E/KNL1/HIST2H4A/HIST1H4J/HIST1H4L/CENPH/HIST1H4C/HIST1H4H/HIST1H2AB/HIST1H2BB/HIST1H2AD/HIST1H2BO/HIST1H2BN/HIST1H2BF/HIST1H2BM/HIST1H2AE/HIST1H2BL/HIST1H2BI/HIST3H2BB/HIST1H4D/HIST1H2BH/HIST1H4A/HIST1H2AJ/HIST1H2BE | | |  |
| R-HSA-774815 | | Nucleosome assembly | | -3.356522585 | | | 0.001002937 | HIST1H2BK/CENPW/CENPU/HIST1H4B/HIST2H2AA3/HIST1H2BD/HIST1H2BJ/HIST1H2BC/HIST1H2BG/HIST1H4E/KNL1/HIST2H4A/HIST1H4J/HIST1H4L/CENPH/HIST1H4C/HIST1H4H/HIST1H2AB/HIST1H2BB/HIST1H2AD/HIST1H2BO/HIST1H2BN/HIST1H2BF/HIST1H2BM/HIST1H2AE/HIST1H2BL/HIST1H2BI/HIST3H2BB/HIST1H4D/HIST1H2BH/HIST1H4A/HIST1H2AJ/HIST1H2BE | | |  |
| R-HSA-427359 | | SIRT1 negatively regulates rRNA expression | | -4.144224042 | | | 0.001002937 | HIST2H2BE/HIST1H2BK/HIST2H3C/HIST1H3E/HIST1H4B/HIST2H2AA3/HIST1H2BD/HIST1H2BJ/HIST1H2BC/HIST1H2BG/HIST1H4E/HIST2H4A/HIST1H4J/HIST1H4L/HIST1H4C/HIST1H3C/HIST1H4H/HIST1H2AB/HIST1H2BB/HIST1H2AD/HIST1H3G/HIST1H3B/HIST1H3H/HIST1H2BO/HIST1H2BN/HIST1H2BF/HIST1H2BM/HIST1H2AE/HIST1H3A/HIST1H2BL/HIST1H2BI/HIST3H2BB/HIST1H4D/HIST1H3J/HIST1H2BH/HIST1H4A/HIST1H2AJ/HIST2H3D/HIST1H2BE/HIST1H3D/HIST1H3F | | |  |
| R-HSA-5334118 | | DNA methylation | | -4.223901169 | | | 0.001002937 | HIST2H2BE/HIST1H2BK/HIST2H3C/HIST1H3E/HIST1H4B/HIST2H2AA3/HIST1H2BD/HIST1H2BJ/HIST1H2BC/HIST1H2BG/HIST1H4E/HIST2H4A/HIST1H4J/HIST1H4L/DNMT3B/HIST1H4C/HIST1H3C/HIST1H4H/HIST1H2AB/HIST1H2BB/HIST1H2AD/HIST1H3G/HIST1H3B/HIST1H3H/HIST1H2BO/HIST1H2BN/HIST1H2BF/HIST1H2BM/HIST1H2AE/HIST1H3A/HIST1H2BL/HIST1H2BI/HIST3H2BB/HIST1H4D/HIST1H3J/HIST1H2BH/HIST1H4A/HIST1H2AJ/HIST2H3D/HIST1H2BE/HIST1H3D/HIST1H3F | | |  |
| R-HSA-5625886 | | Activated PKN1 stimulates transcription of AR (androgen receptor) regulated genes KLK2 and KLK3 | | -4.172145966 | | | 0.001002937 | HIST2H2BE/HIST1H2BK/HIST2H3C/HIST1H3E/HIST1H4B/HIST2H2AA3/HIST1H2BD/HIST1H2BJ/HIST1H2BC/HIST1H2BG/HIST1H4E/HIST2H4A/HIST1H4J/HIST1H4L/HIST1H4C/HIST1H3C/HIST1H4H/HIST1H2AB/HIST1H2BB/HIST1H2AD/HIST1H3G/HIST1H3B/HIST1H3H/HIST1H2BO/HIST1H2BN/HIST1H2BF/HIST1H2BM/HIST1H2AE/HIST1H3A/HIST1H2BL/HIST1H2BI/HIST3H2BB/HIST1H4D/HIST1H3J/HIST1H2BH/HIST1H4A/HIST1H2AJ/HIST2H3D/HIST1H2BE/HIST1H3D/HIST1H3F | | |  |
| R-HSA-73728 | | RNA Polymerase I Promoter Opening | | -4.172145966 | | | 0.001002937 | HIST2H2BE/HIST1H2BK/HIST2H3C/HIST1H3E/HIST1H4B/HIST2H2AA3/HIST1H2BD/HIST1H2BJ/HIST1H2BC/HIST1H2BG/HIST1H4E/HIST2H4A/HIST1H4J/HIST1H4L/HIST1H4C/HIST1H3C/HIST1H4H/HIST1H2AB/HIST1H2BB/HIST1H2AD/HIST1H3G/HIST1H3B/HIST1H3H/HIST1H2BO/HIST1H2BN/HIST1H2BF/HIST1H2BM/HIST1H2AE/HIST1H3A/HIST1H2BL/HIST1H2BI/HIST3H2BB/HIST1H4D/HIST1H3J/HIST1H2BH/HIST1H4A/HIST1H2AJ/HIST2H3D/HIST1H2BE/HIST1H3D/HIST1H3F | | |  |
| R-HSA-110328 | | Recognition and association of DNA glycosylase with site containing an affected pyrimidine | | -3.498282695 | | | 0.001002937 | HIST2H2AA3/HIST1H2BD/HIST1H2BJ/HIST1H2BC/HIST1H2BG/HIST1H4E/HIST2H4A/HIST1H4J/HIST1H4L/HIST1H4C/HIST1H4H/HIST1H2AB/HIST1H2BB/HIST1H2AD/HIST1H2BO/HIST1H2BN/HIST1H2BF/HIST1H2BM/HIST1H2AE/HIST1H2BL/HIST1H2BI/HIST3H2BB/HIST1H4D/HIST1H2BH/HIST1H4A/HIST1H2AJ/HIST1H2BE | | |  |
| R-HSA-110329 | | Cleavage of the damaged pyrimidine | | -3.498282695 | | | 0.001002937 | HIST2H2AA3/HIST1H2BD/HIST1H2BJ/HIST1H2BC/HIST1H2BG/HIST1H4E/HIST2H4A/HIST1H4J/HIST1H4L/HIST1H4C/HIST1H4H/HIST1H2AB/HIST1H2BB/HIST1H2AD/HIST1H2BO/HIST1H2BN/HIST1H2BF/HIST1H2BM/HIST1H2AE/HIST1H2BL/HIST1H2BI/HIST3H2BB/HIST1H4D/HIST1H2BH/HIST1H4A/HIST1H2AJ/HIST1H2BE | | |  |
| R-HSA-1221632 | | Meiotic synapsis | | -3.56690581 | | | 0.001002937 | HIST1H2AC/HIST4H4/LMNB1/HIST2H2BE/HIST1H2BK/HIST1H4B/HIST2H2AA3/HIST1H2BD/HIST1H2BJ/HIST1H2BC/HIST1H2BG/HIST1H4E/HIST2H4A/HIST1H4J/HIST1H4L/HIST1H4C/HIST1H4H/HIST1H2AB/HIST1H2BB/HIST1H2AD/HIST1H2BO/HIST1H2BN/HIST1H2BF/HIST1H2BM/HIST1H2AE/HIST1H2BL/HIST1H2BI/HIST3H2BB/HIST1H4D/HIST1H2BH/HIST1H4A/HIST1H2AJ/HIST1H2BE | | |  |
| R-HSA-73928 | | Depyrimidination | | -3.498282695 | | | 0.001002937 | HIST2H2AA3/HIST1H2BD/HIST1H2BJ/HIST1H2BC/HIST1H2BG/HIST1H4E/HIST2H4A/HIST1H4J/HIST1H4L/HIST1H4C/HIST1H4H/HIST1H2AB/HIST1H2BB/HIST1H2AD/HIST1H2BO/HIST1H2BN/HIST1H2BF/HIST1H2BM/HIST1H2AE/HIST1H2BL/HIST1H2BI/HIST3H2BB/HIST1H4D/HIST1H2BH/HIST1H4A/HIST1H2AJ/HIST1H2BE | | |  |
| R-HSA-73929 | | Base-Excision Repair. AP Site Formation | | -3.498282695 | | | 0.001002937 | HIST2H2AA3/HIST1H2BD/HIST1H2BJ/HIST1H2BC/HIST1H2BG/HIST1H4E/HIST2H4A/HIST1H4J/HIST1H4L/HIST1H4C/HIST1H4H/HIST1H2AB/HIST1H2BB/HIST1H2AD/HIST1H2BO/HIST1H2BN/HIST1H2BF/HIST1H2BM/HIST1H2AE/HIST1H2BL/HIST1H2BI/HIST3H2BB/HIST1H4D/HIST1H2BH/HIST1H4A/HIST1H2AJ/HIST1H2BE | | |  |
| R-HSA-3214841 | | PKMTs methylate histone lysines | | -3.092650779 | | | 0.001002937 | HIST4H4/HIST1H3I/HIST2H3C/HIST1H3E/HIST1H4B/HIST1H4E/HIST2H4A/HIST1H4J/HIST1H4L/HIST1H4C/HIST1H3C/HIST1H4H/HIST1H3G/HIST1H3B/HIST1H3H/HIST1H3A/HIST1H4D/HIST1H3J/HIST1H4A/HIST2H3D/HIST1H3D/HIST1H3F | | |  |
| R-HSA-5693606 | | DNA Double Strand Break Response | | -3.428928253 | | | 0.001002937 | HIST1H2BK/BARD1/HIST1H4B/HIST1H2BD/HIST1H2BJ/HIST1H2BC/HIST1H2BG/HIST1H4E/HIST2H4A/HIST1H4J/HIST1H4L/HIST1H4C/HIST1H4H/HIST1H2BB/HIST1H2BO/HIST1H2BN/HIST1H2BF/HIST1H2BM/HIST1H2BL/HIST1H2BI/HIST3H2BB/HIST1H4D/HIST1H2BH/HIST1H4A/HIST1H2BE | | |  |
| R-HSA-1912408 | | Pre-NOTCH Transcription and Translation | | -4.094519128 | | | 0.001002937 | HIST2H2BE/HIST1H2BK/HIST2H3C/HIST1H3E/HIST1H4B/HIST2H2AA3/HIST1H2BD/HIST1H2BJ/HIST1H2BC/HIST1H2BG/HIST1H4E/HIST2H4A/HIST1H4J/HIST1H4L/HIST1H4C/HIST1H3C/HIST1H4H/HIST1H2AB/HIST1H2BB/HIST1H2AD/HIST1H3G/HIST1H3B/HIST1H3H/HIST1H2BO/HIST1H2BN/HIST1H2BF/HIST1H2BM/HIST1H2AE/HIST1H3A/HIST1H2BL/HIST1H2BI/HIST3H2BB/HIST1H4D/HIST1H3J/HIST1H2BH/HIST1H4A/HIST1H2AJ/HIST2H3D/HIST1H2BE/HIST1H3D/HIST1H3F | | |  |
| R-HSA-5625740 | | RHO GTPases activate PKNs | | -4.174079524 | | | 0.001002937 | HIST2H2BE/HIST1H2BK/HIST2H3C/HIST1H3E/HIST1H4B/HIST2H2AA3/CDC25C/HIST1H2BD/HIST1H2BJ/HIST1H2BC/HIST1H2BG/HIST1H4E/HIST2H4A/HIST1H4J/HIST1H4L/HIST1H4C/HIST1H3C/HIST1H4H/HIST1H2AB/HIST1H2BB/HIST1H2AD/HIST1H3G/HIST1H3B/HIST1H3H/HIST1H2BO/HIST1H2BN/HIST1H2BF/HIST1H2BM/HIST1H2AE/HIST1H3A/HIST1H2BL/HIST1H2BI/HIST3H2BB/HIST1H4D/HIST1H3J/HIST1H2BH/HIST1H4A/HIST1H2AJ/HIST2H3D/HIST1H2BE/HIST1H3D/HIST1H3F | | |  |
| R-HSA-8936459 | | RUNX1 regulates genes involved in megakaryocyte differentiation and platelet function | | -4.104494209 | | | 0.001002937 | HIST2H2BE/HIST1H2BK/HIST2H3C/HIST1H3E/HIST1H4B/HIST2H2AA3/HIST1H2BD/HIST1H2BJ/HIST1H2BC/HIST1H2BG/HIST1H4E/HIST2H4A/HIST1H4J/HIST1H4L/HIST1H4C/HIST1H3C/HIST1H4H/HIST1H2AB/HIST1H2BB/HIST1H2AD/HIST1H3G/HIST1H3B/HIST1H3H/HIST1H2BO/HIST1H2BN/HIST1H2BF/HIST1H2BM/HIST1H2AE/HIST1H3A/HIST1H2BL/HIST1H2BI/HIST3H2BB/HIST1H4D/HIST1H3J/HIST1H2BH/HIST1H4A/HIST1H2AJ/HIST2H3D/HIST1H2BE/HIST1H3D/HIST1H3F | | |  |
| R-HSA-5693532 | | DNA Double-Strand Break Repair | | -2.73227357 | | | 0.001002937 | HIST1H2BK/BARD1/PCNA/DNA2/HIST1H4B/RPA3/CCNA2/HIST1H2BD/HIST1H2BJ/HIST1H2BC/HIST1H2BG/HIST1H4E/HIST2H4A/HIST1H4J/HIST1H4L/HIST1H4C/HIST1H4H/HIST1H2BB/HIST1H2BO/HIST1H2BN/HIST1H2BF/HIST1H2BM/HIST1H2BL/HIST1H2BI/HIST3H2BB/HIST1H4D/HIST1H2BH/HIST1H4A/HIST1H2BE/SLX1B | | |  |
| R-HSA-912446 | | Meiotic recombination | | -3.830580743 | | | 0.001002937 | HIST2H2BE/HIST1H2BK/HIST2H3C/HIST1H3E/HIST1H4B/RPA3/HIST2H2AA3/HIST1H2BD/HIST1H2BJ/HIST1H2BC/HIST1H2BG/HIST1H4E/HIST2H4A/HIST1H4J/HIST1H4L/HIST1H4C/HIST1H3C/HIST1H4H/HIST1H2AB/HIST1H2BB/HIST1H2AD/HIST1H3G/HIST1H3B/HIST1H3H/HIST1H2BO/HIST1H2BN/HIST1H2BF/HIST1H2BM/HIST1H2AE/HIST1H3A/HIST1H2BL/HIST1H2BI/HIST3H2BB/HIST1H4D/HIST1H3J/HIST1H2BH/HIST1H4A/HIST1H2AJ/HIST2H3D/HIST1H2BE/HIST1H3D/HIST1H3F | | |  |
| R-HSA-9018519 | | Estrogen-dependent gene expression | | -3.728070576 | | | 0.001002937 | HIST2H2BE/HIST1H2BK/HIST2H3C/HIST1H3E/HIST1H4B/HIST2H2AA3/HIST1H2BD/HIST1H2BJ/HIST1H2BC/HIST1H2BG/HIST1H4E/HIST2H4A/HIST1H4J/HIST1H4L/HIST1H4C/HIST1H3C/HIST1H4H/HIST1H2AB/HIST1H2BB/HIST1H2AD/HIST1H3G/HIST1H3B/HIST1H3H/HIST1H2BO/HIST1H2BN/HIST1H2BF/HIST1H2BM/HIST1H2AE/HIST1H3A/HIST1H2BL/HIST1H2BI/HIST3H2BB/HIST1H4D/HIST1H3J/HIST1H2BH/HIST1H4A/HIST1H2AJ/HIST2H3D/HIST1H2BE/HIST1H3D/HIST1H3F | | |  |
| R-HSA-5578749 | | Transcriptional regulation by small RNAs | | -3.752745667 | | | 0.001002937 | HIST1H2AC/HIST4H4/NUP37/HIST1H3I/HIST2H2BE/HIST1H2BK/HIST2H3C/HIST1H3E/HIST1H4B/HIST2H2AA3/HIST1H2BD/HIST1H2BJ/HIST1H2BC/HIST1H2BG/HIST1H4E/HIST2H4A/HIST1H4J/HIST1H4L/HIST1H4C/HIST1H3C/HIST1H4H/HIST1H2AB/HIST1H2BB/HIST1H2AD/HIST1H3G/HIST1H3B/HIST1H3H/HIST1H2BO/HIST1H2BN/HIST1H2BF/HIST1H2BM/HIST1H2AE/HIST1H3A/HIST1H2BL/HIST1H2BI/HIST3H2BB/HIST1H4D/HIST1H3J/HIST1H2BH/HIST1H4A/HIST1H2AJ/HIST2H3D/HIST1H2BE/HIST1H3D/HIST1H3F | | |  |
| R-HSA-5693565 | | Recruitment and ATM-mediated phosphorylation of repair and signaling proteins at DNA double strand breaks | | -3.478583102 | | | 0.001002937 | HIST1H2BK/BARD1/HIST1H4B/HIST1H2BD/HIST1H2BJ/HIST1H2BC/HIST1H2BG/HIST1H4E/HIST2H4A/HIST1H4J/HIST1H4L/HIST1H4C/HIST1H4H/HIST1H2BB/HIST1H2BO/HIST1H2BN/HIST1H2BF/HIST1H2BM/HIST1H2BL/HIST1H2BI/HIST3H2BB/HIST1H4D/HIST1H2BH/HIST1H4A/HIST1H2BE | | |  |
| R-HSA-5693571 | | Nonhomologous End-Joining (NHEJ) | | -3.323702964 | | | 0.001002937 | HIST1H2BK/BARD1/HIST1H4B/HIST1H2BD/HIST1H2BJ/HIST1H2BC/HIST1H2BG/HIST1H4E/HIST2H4A/HIST1H4J/HIST1H4L/HIST1H4C/HIST1H4H/HIST1H2BB/HIST1H2BO/HIST1H2BN/HIST1H2BF/HIST1H2BM/HIST1H2BL/HIST1H2BI/HIST3H2BB/HIST1H4D/HIST1H2BH/HIST1H4A/HIST1H2BE | | |  |
| R-HSA-212165 | | Epigenetic regulation of gene expression | | -3.890184882 | | | 0.001002937 | HIST2H2BE/HIST1H2BK/HIST2H3C/HIST1H3E/HIST1H4B/HIST2H2AA3/HIST1H2BD/HIST1H2BJ/HIST1H2BC/HIST1H2BG/HIST1H4E/HIST2H4A/HIST1H4J/HIST1H4L/DNMT3B/HIST1H4C/HIST1H3C/HIST1H4H/HIST1H2AB/HIST1H2BB/HIST1H2AD/HIST1H3G/HIST1H3B/HIST1H3H/HIST1H2BO/HIST1H2BN/HIST1H2BF/HIST1H2BM/HIST1H2AE/HIST1H3A/HIST1H2BL/HIST1H2BI/HIST3H2BB/HIST1H4D/HIST1H3J/HIST1H2BH/HIST1H4A/HIST1H2AJ/HIST2H3D/HIST1H2BE/HIST1H3D/HIST1H3F | | |  |
| R-HSA-2559582 | | Senescence-Associated Secretory Phenotype (SASP) | | -4.079682571 | | | 0.001002937 | HIST2H2BE/HIST1H2BK/HIST2H3C/HIST1H3E/HIST1H4B/HIST2H2AA3/CCNA2/RPS6KA1/HIST1H2BD/HIST1H2BJ/HIST1H2BC/HIST1H2BG/HIST1H4E/HIST2H4A/HIST1H4J/HIST1H4L/HIST1H4C/HIST1H3C/HIST1H4H/HIST1H2AB/HIST1H2BB/HIST1H2AD/HIST1H3G/HIST1H3B/HIST1H3H/HIST1H2BO/HIST1H2BN/HIST1H2BF/HIST1H2BM/HIST1H2AE/HIST1H3A/HIST1H2BL/HIST1H2BI/HIST3H2BB/HIST1H4D/HIST1H3J/HIST1H2BH/HIST1H4A/HIST1H2AJ/HIST2H3D/HIST1H2BE/HIST1H3D/HIST1H3F | | |  |
| R-HSA-110330 | | Recognition and association of DNA glycosylase with site containing an affected purine | | -3.533832366 | | | 0.001002937 | HIST1H2AC/HIST4H4/HIST2H2BE/HIST1H2BK/HIST1H4B/HIST2H2AA3/HIST1H2BD/HIST1H2BJ/HIST1H2BC/HIST1H2BG/HIST1H4E/HIST2H4A/HIST1H4J/HIST1H4L/HIST1H4C/HIST1H4H/HIST1H2AB/HIST1H2BB/HIST1H2AD/HIST1H2BO/HIST1H2BN/HIST1H2BF/HIST1H2BM/HIST1H2AE/HIST1H2BL/HIST1H2BI/HIST3H2BB/HIST1H4D/HIST1H2BH/HIST1H4A/HIST1H2AJ/HIST1H2BE | | |  |
| R-HSA-110331 | | Cleavage of the damaged purine | | -3.533832366 | | | 0.001002937 | HIST1H2AC/HIST4H4/HIST2H2BE/HIST1H2BK/HIST1H4B/HIST2H2AA3/HIST1H2BD/HIST1H2BJ/HIST1H2BC/HIST1H2BG/HIST1H4E/HIST2H4A/HIST1H4J/HIST1H4L/HIST1H4C/HIST1H4H/HIST1H2AB/HIST1H2BB/HIST1H2AD/HIST1H2BO/HIST1H2BN/HIST1H2BF/HIST1H2BM/HIST1H2AE/HIST1H2BL/HIST1H2BI/HIST3H2BB/HIST1H4D/HIST1H2BH/HIST1H4A/HIST1H2AJ/HIST1H2BE | | |  |
| R-HSA-5250913 | | Positive epigenetic regulation of rRNA expression | | -3.957097239 | | | 0.001002937 | HIST2H2BE/HIST1H2BK/HIST2H3C/HIST1H3E/HIST1H4B/HIST2H2AA3/HIST1H2BD/HIST1H2BJ/HIST1H2BC/HIST1H2BG/HIST1H4E/HIST2H4A/HIST1H4J/HIST1H4L/HIST1H4C/HIST1H3C/HIST1H4H/HIST1H2AB/HIST1H2BB/HIST1H2AD/HIST1H3G/HIST1H3B/HIST1H3H/HIST1H2BO/HIST1H2BN/HIST1H2BF/HIST1H2BM/HIST1H2AE/HIST1H3A/HIST1H2BL/HIST1H2BI/HIST3H2BB/HIST1H4D/HIST1H3J/HIST1H2BH/HIST1H4A/HIST1H2AJ/HIST2H3D/HIST1H2BE/HIST1H3D/HIST1H3F | | |  |
| R-HSA-73927 | | Depurination | | -3.533832366 | | | 0.001002937 | HIST1H2AC/HIST4H4/HIST2H2BE/HIST1H2BK/HIST1H4B/HIST2H2AA3/HIST1H2BD/HIST1H2BJ/HIST1H2BC/HIST1H2BG/HIST1H4E/HIST2H4A/HIST1H4J/HIST1H4L/HIST1H4C/HIST1H4H/HIST1H2AB/HIST1H2BB/HIST1H2AD/HIST1H2BO/HIST1H2BN/HIST1H2BF/HIST1H2BM/HIST1H2AE/HIST1H2BL/HIST1H2BI/HIST3H2BB/HIST1H4D/HIST1H2BH/HIST1H4A/HIST1H2AJ/HIST1H2BE | | |  |
| R-HSA-171306 | | Packaging Of Telomere Ends | | -3.580000334 | | | 0.001002937 | HIST1H2AC/HIST4H4/HIST2H2BE/HIST1H2BK/HIST1H4B/HIST2H2AA3/HIST1H2BD/HIST1H2BJ/HIST1H2BC/HIST1H2BG/HIST1H4E/HIST2H4A/HIST1H4J/HIST1H4L/HIST1H4C/HIST1H4H/HIST1H2AB/HIST1H2BB/HIST1H2AD/HIST1H2BO/HIST1H2BN/HIST1H2BF/HIST1H2BM/HIST1H2AE/HIST1H2BL/HIST1H2BI/HIST3H2BB/HIST1H4D/HIST1H2BH/HIST1H4A/HIST1H2AJ/HIST1H2BE | | |  |
| R-HSA-427413 | | NoRC negatively regulates rRNA expression | | -4.137589821 | | | 0.001002937 | HIST2H2BE/HIST1H2BK/HIST2H3C/HIST1H3E/HIST1H4B/HIST2H2AA3/HIST1H2BD/HIST1H2BJ/HIST1H2BC/HIST1H2BG/HIST1H4E/HIST2H4A/HIST1H4J/HIST1H4L/DNMT3B/HIST1H4C/HIST1H3C/HIST1H4H/HIST1H2AB/HIST1H2BB/HIST1H2AD/HIST1H3G/HIST1H3B/HIST1H3H/HIST1H2BO/HIST1H2BN/HIST1H2BF/HIST1H2BM/HIST1H2AE/HIST1H3A/HIST1H2BL/HIST1H2BI/HIST3H2BB/HIST1H4D/HIST1H3J/HIST1H2BH/HIST1H4A/HIST1H2AJ/HIST2H3D/HIST1H2BE/HIST1H3D/HIST1H3F | | |  |
| R-HSA-977225 | | Amyloid fiber formation | | -4.128837685 | | | 0.001002937 | HIST2H2BE/HIST1H2BK/HIST2H3C/HIST1H3E/HIST1H4B/HIST2H2AA3/HIST1H2BD/HIST1H2BJ/HIST1H2BC/HIST1H2BG/HIST1H4E/HIST2H4A/HIST1H4J/HIST1H4L/HIST1H4C/HIST1H3C/HIST1H4H/HIST1H2AB/HIST1H2BB/HIST1H2AD/HIST1H3G/HIST1H3B/HIST1H3H/HIST1H2BO/HIST1H2BN/HIST1H2BF/HIST1H2BM/HIST1H2AE/HIST1H3A/HIST1H2BL/HIST1H2BI/HIST3H2BB/HIST1H4D/HIST1H3J/HIST1H2BH/HIST1H4A/HIST1H2AJ/HIST2H3D/HIST1H2BE/HIST1H3D/TSPAN33/HIST1H3F | | |  |
| R-HSA-73772 | | RNA Polymerase I Promoter Escape | | -4.05794594 | | | 0.001002937 | HIST2H2BE/HIST1H2BK/HIST2H3C/HIST1H3E/HIST1H4B/HIST2H2AA3/HIST1H2BD/HIST1H2BJ/HIST1H2BC/HIST1H2BG/HIST1H4E/HIST2H4A/HIST1H4J/HIST1H4L/HIST1H4C/HIST1H3C/HIST1H4H/HIST1H2AB/HIST1H2BB/HIST1H2AD/HIST1H3G/HIST1H3B/HIST1H3H/HIST1H2BO/HIST1H2BN/HIST1H2BF/HIST1H2BM/HIST1H2AE/HIST1H3A/HIST1H2BL/HIST1H2BI/HIST3H2BB/HIST1H4D/HIST1H3J/HIST1H2BH/HIST1H4A/HIST1H2AJ/HIST2H3D/HIST1H2BE/HIST1H3D/HIST1H3F | | |  |
| R-HSA-212300 | | PRC2 methylates histones and DNA | | -4.136370152 | | | 0.001002937 | HIST2H2BE/HIST1H2BK/HIST2H3C/HIST1H3E/HIST1H4B/HIST2H2AA3/HIST1H2BD/HIST1H2BJ/HIST1H2BC/HIST1H2BG/HIST1H4E/HIST2H4A/HIST1H4J/HIST1H4L/DNMT3B/HIST1H4C/HIST1H3C/HIST1H4H/HIST1H2AB/HIST1H2BB/HIST1H2AD/HIST1H3G/HIST1H3B/HIST1H3H/HIST1H2BO/HIST1H2BN/HIST1H2BF/HIST1H2BM/HIST1H2AE/HIST1H3A/HIST1H2BL/HIST1H2BI/HIST3H2BB/HIST1H4D/HIST1H3J/HIST1H2BH/HIST1H4A/HIST1H2AJ/HIST2H3D/HIST1H2BE/HIST1H3D/HIST1H3F | | |  |
| R-HSA-427389 | | ERCC6 (CSB) and EHMT2 (G9a) positively regulate rRNA expression | | -4.056725354 | | | 0.001002937 | HIST2H2BE/HIST1H2BK/HIST2H3C/HIST1H3E/HIST1H4B/HIST2H2AA3/HIST1H2BD/HIST1H2BJ/HIST1H2BC/HIST1H2BG/HIST1H4E/HIST2H4A/HIST1H4J/HIST1H4L/HIST1H4C/HIST1H3C/HIST1H4H/HIST1H2AB/HIST1H2BB/HIST1H2AD/HIST1H3G/HIST1H3B/HIST1H3H/HIST1H2BO/HIST1H2BN/HIST1H2BF/HIST1H2BM/HIST1H2AE/HIST1H3A/HIST1H2BL/HIST1H2BI/HIST3H2BB/HIST1H4D/HIST1H3J/HIST1H2BH/HIST1H4A/HIST1H2AJ/HIST2H3D/HIST1H2BE/HIST1H3D/HIST1H3F | | |  |
| R-HSA-5250924 | | B-WICH complex positively regulates rRNA expression | | -4.061326297 | | | 0.001002937 | HIST2H2BE/HIST1H2BK/HIST2H3C/HIST1H3E/HIST1H4B/HIST2H2AA3/HIST1H2BD/HIST1H2BJ/HIST1H2BC/HIST1H2BG/HIST1H4E/HIST2H4A/HIST1H4J/HIST1H4L/HIST1H4C/HIST1H3C/HIST1H4H/HIST1H2AB/HIST1H2BB/HIST1H2AD/HIST1H3G/HIST1H3B/HIST1H3H/HIST1H2BO/HIST1H2BN/HIST1H2BF/HIST1H2BM/HIST1H2AE/HIST1H3A/HIST1H2BL/HIST1H2BI/HIST3H2BB/HIST1H4D/HIST1H3J/HIST1H2BH/HIST1H4A/HIST1H2AJ/HIST2H3D/HIST1H2BE/HIST1H3D/HIST1H3F | | |  |
| R-HSA-8852135 | | Protein ubiquitination | | -2.874475955 | | | 0.001002937 | RNF144A/PCNA/UBE2T/HIST1H2BD/HIST1H2BJ/HIST1H2BC/HIST1H2BG/HIST1H2BB/HIST1H2BO/HIST1H2BN/HIST1H2BF/HIST1H2BM/HIST1H2BL/HIST1H2BI/HIST1H2BH/HIST1H2BE | | |  |
| R-HSA-5693538 | | Homology Directed Repair | | -2.873340434 | | | 0.001002937 | HIST1H2BK/BARD1/PCNA/DNA2/HIST1H4B/RPA3/CCNA2/HIST1H2BD/HIST1H2BJ/HIST1H2BC/HIST1H2BG/HIST1H4E/HIST2H4A/HIST1H4J/HIST1H4L/HIST1H4C/HIST1H4H/HIST1H2BB/HIST1H2BO/HIST1H2BN/HIST1H2BF/HIST1H2BM/HIST1H2BL/HIST1H2BI/HIST3H2BB/HIST1H4D/HIST1H2BH/HIST1H4A/HIST1H2BE/SLX1B | | |  |
| R-HSA-2559580 | | Oxidative Stress Induced Senescence | | -3.890948395 | | | 0.001002937 | HIST2H2BE/HIST1H2BK/HIST2H3C/HIST1H3E/HIST1H4B/HIST2H2AA3/HIST1H2BD/HIST1H2BJ/HIST1H2BC/HIST1H2BG/HIST1H4E/HIST2H4A/HIST1H4J/HIST1H4L/HIST1H4C/HIST1H3C/HIST1H4H/HIST1H2AB/HIST1H2BB/HIST1H2AD/HIST1H3G/HIST1H3B/HIST1H3H/HIST1H2BO/HIST1H2BN/HIST1H2BF/HIST1H2BM/HIST1H2AE/HIST1H3A/HIST1H2BL/HIST1H2BI/HIST3H2BB/HIST1H4D/HIST1H3J/HIST1H2BH/HIST1H4A/HIST1H2AJ/HIST2H3D/HIST1H2BE/HIST1H3D/HIST1H3F | | |  |
| R-HSA-5693567 | | HDR through Homologous Recombination (HRR) or Single Strand Annealing (SSA) | | -2.873340434 | | | 0.001002937 | HIST1H2BK/BARD1/PCNA/DNA2/HIST1H4B/RPA3/CCNA2/HIST1H2BD/HIST1H2BJ/HIST1H2BC/HIST1H2BG/HIST1H4E/HIST2H4A/HIST1H4J/HIST1H4L/HIST1H4C/HIST1H4H/HIST1H2BB/HIST1H2BO/HIST1H2BN/HIST1H2BF/HIST1H2BM/HIST1H2BL/HIST1H2BI/HIST3H2BB/HIST1H4D/HIST1H2BH/HIST1H4A/HIST1H2BE/SLX1B | | |  |
| R-HSA-5617472 | | Activation of anterior HOX genes in hindbrain development during early embryogenesis | | -3.89588757 | | | 0.001002937 | HIST2H2BE/HIST1H2BK/HIST2H3C/HIST1H3E/HIST1H4B/HIST2H2AA3/HIST1H2BD/HIST1H2BJ/HIST1H2BC/HIST1H2BG/HIST1H4E/HIST2H4A/HIST1H4J/HIST1H4L/HIST1H4C/HIST1H3C/HIST1H4H/HIST1H2AB/HIST1H2BB/HIST1H2AD/HIST1H3G/HIST1H3B/HIST1H3H/HIST1H2BO/HIST1H2BN/HIST1H2BF/HIST1H2BM/HIST1H2AE/HIST1H3A/HIST1H2BL/HIST1H2BI/HIST3H2BB/HIST1H4D/HIST1H3J/HIST1H2BH/HIST1H4A/HIST1H2AJ/HIST2H3D/HIST1H2BE/HIST1H3D/HIST1H3F | | |  |
| R-HSA-5619507 | | Activation of HOX genes during differentiation | | -3.89588757 | | | 0.001002937 | HIST2H2BE/HIST1H2BK/HIST2H3C/HIST1H3E/HIST1H4B/HIST2H2AA3/HIST1H2BD/HIST1H2BJ/HIST1H2BC/HIST1H2BG/HIST1H4E/HIST2H4A/HIST1H4J/HIST1H4L/HIST1H4C/HIST1H3C/HIST1H4H/HIST1H2AB/HIST1H2BB/HIST1H2AD/HIST1H3G/HIST1H3B/HIST1H3H/HIST1H2BO/HIST1H2BN/HIST1H2BF/HIST1H2BM/HIST1H2AE/HIST1H3A/HIST1H2BL/HIST1H2BI/HIST3H2BB/HIST1H4D/HIST1H3J/HIST1H2BH/HIST1H4A/HIST1H2AJ/HIST2H3D/HIST1H2BE/HIST1H3D/HIST1H3F | | |  |
| R-HSA-1500620 | | Meiosis | | -3.866271412 | | | 0.001002937 | LMNB1/HIST2H2BE/HIST1H2BK/HIST2H3C/HIST1H3E/HIST1H4B/RPA3/HIST2H2AA3/HIST1H2BD/HIST1H2BJ/HIST1H2BC/HIST1H2BG/HIST1H4E/HIST2H4A/HIST1H4J/HIST1H4L/HIST1H4C/HIST1H3C/HIST1H4H/HIST1H2AB/HIST1H2BB/HIST1H2AD/HIST1H3G/HIST1H3B/HIST1H3H/HIST1H2BO/HIST1H2BN/HIST1H2BF/HIST1H2BM/HIST1H2AE/HIST1H3A/HIST1H2BL/HIST1H2BI/HIST3H2BB/HIST1H4D/HIST1H3J/HIST1H2BH/HIST1H4A/HIST1H2AJ/HIST2H3D/HIST1H2BE/HIST1H3D/HIST1H3F | | |  |
| R-HSA-1474165 | | Reproduction | | -3.837524384 | | | 0.001002937 | LMNB1/HIST2H2BE/HIST1H2BK/HIST2H3C/HIST1H3E/HIST1H4B/RPA3/HIST2H2AA3/HIST1H2BD/HIST1H2BJ/HIST1H2BC/HIST1H2BG/HIST1H4E/HIST2H4A/HIST1H4J/HIST1H4L/HIST1H4C/HIST1H3C/HIST1H4H/HIST1H2AB/HIST1H2BB/HIST1H2AD/HIST1H3G/HIST1H3B/HIST1H3H/HIST1H2BO/HIST1H2BN/HIST1H2BF/HIST1H2BM/HIST1H2AE/HIST1H3A/HIST1H2BL/HIST1H2BI/HIST3H2BB/HIST1H4D/HIST1H3J/HIST1H2BH/HIST1H4A/HIST1H2AJ/HIST2H3D/HIST1H2BE/HIST1H3D/HIST1H3F | | |  |
| R-HSA-211000 | | Gene Silencing by RNA | | -3.620834659 | | | 0.001002937 | HIST2H2BE/HIST1H2BK/HIST2H3C/HIST1H3E/HIST1H4B/HIST2H2AA3/HIST1H2BD/HIST1H2BJ/HIST1H2BC/HIST1H2BG/HIST1H4E/HIST2H4A/HIST1H4J/HIST1H4L/HIST1H4C/HIST1H3C/HIST1H4H/HIST1H2AB/HIST1H2BB/HIST1H2AD/HIST1H3G/HIST1H3B/HIST1H3H/HIST1H2BO/HIST1H2BN/HIST1H2BF/HIST1H2BM/HIST1H2AE/HIST1H3A/HIST1H2BL/HIST1H2BI/HIST3H2BB/HIST1H4D/HIST1H3J/HIST1H2BH/HIST1H4A/HIST1H2AJ/HIST2H3D/HIST1H2BE/HIST1H3D/HIST1H3F | | |  |
| R-HSA-8939236 | | RUNX1 regulates transcription of genes involved in differentiation of HSCs | | -3.717902935 | | | 0.001002937 | HIST2H2BE/HIST1H2BK/HIST2H3C/HIST1H3E/PSME2/HIST1H4B/HIST2H2AA3/HIST1H2BD/HIST1H2BJ/HIST1H2BC/HIST1H2BG/HIST1H4E/HIST2H4A/HIST1H4J/HIST1H4L/HIST1H4C/HIST1H3C/HIST1H4H/HIST1H2AB/HIST1H2BB/HIST1H2AD/HIST1H3G/HIST1H3B/HIST1H3H/HIST1H2BO/HIST1H2BN/HIST1H2BF/HIST1H2BM/HIST1H2AE/HIST1H3A/HIST1H2BL/HIST1H2BI/HIST3H2BB/HIST1H4D/HIST1H3J/HIST1H2BH/HIST1H4A/HIST1H2AJ/HIST2H3D/HIST1H2BE/HIST1H3D/HIST1H3F | | |  |
| R-HSA-5688426 | | Deubiquitination | | -3.13668719 | | | 0.001002937 | HIST1H2AG/HIST2H2BE/HIST1H2BK/BARD1/HIST2H2BF/PSME2/HIST2H2AA3/CCNA2/HIST1H2BD/HIST1H2BJ/HIST1H2BC/HIST1H2BG/CDK1/HIST1H2AL/HIST2H2AB/HIST1H2AK/HIST1H2AB/HIST1H2BB/HIST1H2AD/HIST1H2AM/HIST1H2AI/HIST1H2AH/HIST1H2BO/HIST1H2BN/HIST1H2BF/HIST1H2BM/HIST1H2AE/AXIN2/HIST1H2BL/HIST1H2BI/HIST3H2BB/HIST1H2BH/HIST1H2AJ/HIST1H2BE/IL33/RNF128 | | |  |
| R-HSA-68875 | | Mitotic Prophase | | -3.842527569 | | | 0.001002937 | HIST1H2AC/HIST4H4/NUP37/SMC2/HIST1H3I/LMNB1/HIST2H2BE/HIST1H2BK/HIST2H3C/HIST1H3E/CCNB2/HIST1H4B/HIST2H2AA3/HIST1H2BD/HIST1H2BJ/HIST1H2BC/HIST1H2BG/HIST1H4E/HIST2H4A/HIST1H4J/CDK1/HIST1H4L/HIST1H4C/HIST1H3C/HIST1H4H/HIST1H2AB/HIST1H2BB/HIST1H2AD/HIST1H3G/HIST1H3B/HIST1H3H/HIST1H2BO/HIST1H2BN/HIST1H2BF/HIST1H2BM/HIST1H2AE/HIST1H3A/HIST1H2BL/HIST1H2BI/HIST3H2BB/HIST1H4D/HIST1H3J/HIST1H2BH/HIST1H4A/HIST1H2AJ/HIST2H3D/HIST1H2BE/HIST1H3D/HIST1H3F | | |  |
| R-HSA-3214815 | | HDACs deacetylate histones | | -4.499462463 | | | 0.001002937 | HIST1H2AG/HIST2H2BE/HIST1H2BK/HIST2H3C/HIST1H3E/HIST2H2BF/HIST1H4B/HIST2H2AA3/HIST1H2BD/HIST1H2BJ/HIST1H2BC/HIST1H2BG/HIST1H4E/HIST2H4A/HIST1H4J/HIST1H4L/HIST1H2AL/HIST1H4C/HIST1H3C/HIST1H4H/HIST2H2AB/HIST1H2AK/HIST1H2AB/HIST1H2BB/HIST1H2AD/HIST1H3G/HIST1H2AM/HIST1H3B/HIST1H3H/HIST1H2AI/HIST1H2AH/HIST1H2BO/HIST1H2BN/HIST1H2BF/HIST1H2BM/HIST1H2AE/HIST1H3A/HIST1H2BL/HIST1H2BI/HIST3H2BB/HIST1H4D/HIST1H3J/HIST1H2BH/HIST1H4A/HIST1H2AJ/HIST2H3D/HIST1H2BE/HIST1H3D/HIST1H3F | | |  |
| R-HSA-3214847 | | HATs acetylate histones | | -4.498378431 | | | 0.001002937 | HIST1H2AG/HIST2H2BE/HIST1H2BK/HIST2H3C/HIST1H3E/HIST2H2BF/HIST1H4B/HIST2H2AA3/HIST1H2BD/HIST1H2BJ/HIST1H2BC/HIST1H2BG/HIST1H4E/HIST2H4A/HIST1H4J/HIST1H4L/HIST1H2AL/HIST1H4C/HIST1H3C/HIST1H4H/HIST2H2AB/HIST1H2AK/HIST1H2AB/HIST1H2BB/HIST1H2AD/HIST1H3G/HIST1H2AM/HIST1H3B/HIST1H3H/HIST1H2AI/HIST1H2AH/HIST1H2BO/HIST1H2BN/HIST1H2BF/HIST1H2BM/HIST1H2AE/HIST1H3A/HIST1H2BL/HIST1H2BI/HIST3H2BB/HIST1H4D/HIST1H3J/HIST1H2BH/HIST1H4A/HIST1H2AJ/HIST2H3D/HIST1H2BE/HIST1H3D/HIST1H3F | | |  |
| R-HSA-3247509 | | Chromatin modifying enzymes | | -3.914665065 | | | 0.001002937 | HIST1H2AG/HIST2H2BE/HIST1H2BK/HIST2H3C/HIST1H3E/HIST2H2BF/HIST1H4B/HIST2H2AA3/HIST1H2BD/HIST1H2BJ/HIST1H2BC/HIST1H2BG/HIST1H4E/HIST2H4A/HIST1H4J/HIST1H4L/HIST1H2AL/HIST1H4C/HIST1H3C/HIST1H4H/HIST2H2AB/HIST1H2AK/HIST1H2AB/HIST1H2BB/HIST1H2AD/HIST1H3G/HIST1H2AM/HIST1H3B/HIST1H3H/HIST1H2AI/HIST1H2AH/HIST1H2BO/HIST1H2BN/HIST1H2BF/HIST1H2BM/HIST1H2AE/HIST1H3A/HIST1H2BL/HIST1H2BI/HIST3H2BB/HIST1H4D/HIST1H3J/HIST1H2BH/HIST1H4A/HIST1H2AJ/HIST2H3D/HIST1H2BE/HIST1H3D/HIST1H3F | | |  |
| R-HSA-4839726 | | Chromatin organization | | -3.914665065 | | | 0.001002937 | HIST1H2AG/HIST2H2BE/HIST1H2BK/HIST2H3C/HIST1H3E/HIST2H2BF/HIST1H4B/HIST2H2AA3/HIST1H2BD/HIST1H2BJ/HIST1H2BC/HIST1H2BG/HIST1H4E/HIST2H4A/HIST1H4J/HIST1H4L/HIST1H2AL/HIST1H4C/HIST1H3C/HIST1H4H/HIST2H2AB/HIST1H2AK/HIST1H2AB/HIST1H2BB/HIST1H2AD/HIST1H3G/HIST1H2AM/HIST1H3B/HIST1H3H/HIST1H2AI/HIST1H2AH/HIST1H2BO/HIST1H2BN/HIST1H2BF/HIST1H2BM/HIST1H2AE/HIST1H3A/HIST1H2BL/HIST1H2BI/HIST3H2BB/HIST1H4D/HIST1H3J/HIST1H2BH/HIST1H4A/HIST1H2AJ/HIST2H3D/HIST1H2BE/HIST1H3D/HIST1H3F | | |  |
| R-HSA-2299718 | | Condensation of Prophase Chromosomes | | -4.067344069 | | | 0.001002937 | HIST1H3I/HIST2H2BE/HIST1H2BK/HIST2H3C/HIST1H3E/HIST1H4B/HIST2H2AA3/HIST1H2BD/HIST1H2BJ/HIST1H2BC/HIST1H2BG/HIST1H4E/HIST2H4A/HIST1H4J/CDK1/HIST1H4L/HIST1H4C/HIST1H3C/HIST1H4H/HIST1H2AB/HIST1H2BB/HIST1H2AD/HIST1H3G/HIST1H3B/HIST1H3H/HIST1H2BO/HIST1H2BN/HIST1H2BF/HIST1H2BM/HIST1H2AE/HIST1H3A/HIST1H2BL/HIST1H2BI/HIST3H2BB/HIST1H4D/HIST1H3J/HIST1H2BH/HIST1H4A/HIST1H2AJ/HIST2H3D/HIST1H2BE/HIST1H3D/HIST1H3F | | |  |
| R-HSA-73864 | | RNA Polymerase I Transcription | | -3.985797192 | | | 0.001002937 | HIST2H2BE/HIST1H2BK/HIST2H3C/HIST1H3E/HIST1H4B/HIST2H2AA3/HIST1H2BD/HIST1H2BJ/HIST1H2BC/HIST1H2BG/HIST1H4E/HIST2H4A/HIST1H4J/HIST1H4L/HIST1H4C/HIST1H3C/HIST1H4H/HIST1H2AB/HIST1H2BB/HIST1H2AD/HIST1H3G/HIST1H3B/HIST1H3H/HIST1H2BO/HIST1H2BN/HIST1H2BF/HIST1H2BM/HIST1H2AE/HIST1H3A/HIST1H2BL/HIST1H2BI/HIST3H2BB/HIST1H4D/HIST1H3J/HIST1H2BH/HIST1H4A/HIST1H2AJ/HIST2H3D/HIST1H2BE/HIST1H3D/HIST1H3F | | |  |
| R-HSA-73854 | | RNA Polymerase I Promoter Clearance | | -3.985797192 | | | 0.001002937 | HIST2H2BE/HIST1H2BK/HIST2H3C/HIST1H3E/HIST1H4B/HIST2H2AA3/HIST1H2BD/HIST1H2BJ/HIST1H2BC/HIST1H2BG/HIST1H4E/HIST2H4A/HIST1H4J/HIST1H4L/HIST1H4C/HIST1H3C/HIST1H4H/HIST1H2AB/HIST1H2BB/HIST1H2AD/HIST1H3G/HIST1H3B/HIST1H3H/HIST1H2BO/HIST1H2BN/HIST1H2BF/HIST1H2BM/HIST1H2AE/HIST1H3A/HIST1H2BL/HIST1H2BI/HIST3H2BB/HIST1H4D/HIST1H3J/HIST1H2BH/HIST1H4A/HIST1H2AJ/HIST2H3D/HIST1H2BE/HIST1H3D/HIST1H3F | | |  |
| R-HSA-157579 | | Telomere Maintenance | | -3.27559548 | | | 0.001002937 | POLA1/HIST1H4F/NHP2/HIST1H2AC/HIST4H4/HIST2H2BE/HIST1H2BK/PCNA/DNA2/HIST1H4B/RPA3/HIST2H2AA3/HIST1H2BD/HIST1H2BJ/HIST1H2BC/HIST1H2BG/HIST1H4E/HIST2H4A/HIST1H4J/HIST1H4L/HIST1H4C/HIST1H4H/HIST1H2AB/HIST1H2BB/HIST1H2AD/HIST1H2BO/HIST1H2BN/HIST1H2BF/HIST1H2BM/HIST1H2AE/HIST1H2BL/HIST1H2BI/HIST3H2BB/HIST1H4D/HIST1H2BH/POLA2/HIST1H4A/HIST1H2AJ/HIST1H2BE | | |  |
| R-HSA-201722 | | Formation of the beta-catenin:TCF transactivating complex | | -4.221346119 | | | 0.001002937 | HIST2H2BE/HIST1H2BK/HIST2H3C/HIST1H3E/HIST1H4B/HIST2H2AA3/HIST1H2BD/HIST1H2BJ/HIST1H2BC/HIST1H2BG/HIST1H4E/HIST2H4A/HIST1H4J/HIST1H4L/HIST1H4C/HIST1H3C/HIST1H4H/HIST1H2AB/HIST1H2BB/TLE1/LEF1/HIST1H2AD/HIST1H3G/HIST1H3B/HIST1H3H/HIST1H2BO/HIST1H2BN/HIST1H2BF/HIST1H2BM/HIST1H2AE/AXIN2/HIST1H3A/HIST1H2BL/HIST1H2BI/HIST3H2BB/HIST1H4D/HIST1H3J/HIST1H2BH/HIST1H4A/HIST1H2AJ/HIST2H3D/HIST1H2BE/HIST1H3D/HIST1H3F | | |  |
| R-HSA-1912422 | | Pre-NOTCH Expression and Processing | | -4.012979961 | | | 0.001002937 | HIST2H2BE/HIST1H2BK/HIST2H3C/HIST1H3E/HIST1H4B/HIST2H2AA3/HIST1H2BD/HIST1H2BJ/HIST1H2BC/HIST1H2BG/HIST1H4E/HIST2H4A/HIST1H4J/HIST1H4L/HIST1H4C/HIST1H3C/HIST1H4H/HIST1H2AB/HIST1H2BB/HIST1H2AD/HIST1H3G/HIST1H3B/HIST1H3H/HIST1H2BO/HIST1H2BN/HIST1H2BF/HIST1H2BM/HIST1H2AE/HIST1H3A/HIST1H2BL/HIST1H2BI/HIST3H2BB/HIST1H4D/HIST1H3J/HIST1H2BH/HIST1H4A/HIST1H2AJ/HIST2H3D/HIST1H2BE/HIST1H3D/HIST1H3F | | |  |
| R-HSA-8878171 | | Transcriptional regulation by RUNX1 | | -3.483493229 | | | 0.001002937 | HIST2H2BE/HIST1H2BK/HIST2H3C/HIST1H3E/PSME2/HIST1H4B/HIST2H2AA3/HIST1H2BD/HIST1H2BJ/HIST1H2BC/HIST1H2BG/HIST1H4E/HIST2H4A/HIST1H4J/HIST1H4L/HIST1H4C/HIST1H3C/HIST1H4H/HIST1H2AB/HIST1H2BB/HIST1H2AD/HIST1H3G/HIST1H3B/HIST1H3H/HIST1H2BO/HIST1H2BN/HIST1H2BF/HIST1H2BM/HIST1H2AE/HIST1H3A/HIST1H2BL/HIST1H2BI/HIST3H2BB/HIST1H4D/HIST1H3J/HIST1H2BH/HIST1H4A/HIST1H2AJ/HIST2H3D/HIST1H2BE/HIST1H3D/HIST1H3F | | |  |
| R-HSA-5250941 | | Negative epigenetic regulation of rRNA expression | | -4.09707874 | | | 0.001002937 | HIST2H2BE/HIST1H2BK/HIST2H3C/HIST1H3E/HIST1H4B/HIST2H2AA3/HIST1H2BD/HIST1H2BJ/HIST1H2BC/HIST1H2BG/HIST1H4E/HIST2H4A/HIST1H4J/HIST1H4L/DNMT3B/HIST1H4C/HIST1H3C/HIST1H4H/HIST1H2AB/HIST1H2BB/HIST1H2AD/HIST1H3G/HIST1H3B/HIST1H3H/HIST1H2BO/HIST1H2BN/HIST1H2BF/HIST1H2BM/HIST1H2AE/HIST1H3A/HIST1H2BL/HIST1H2BI/HIST3H2BB/HIST1H4D/HIST1H3J/HIST1H2BH/HIST1H4A/HIST1H2AJ/HIST2H3D/HIST1H2BE/HIST1H3D/HIST1H3F | | |  |
| R-HSA-3214842 | | HDMs demethylate histones | | -3.182487316 | | | 0.001002937 | HIST4H4/HIST1H3I/HIST2H3C/HIST1H3E/HIST1H4B/HIST1H4E/HIST2H4A/HIST1H4J/HIST1H4L/HIST1H4C/HIST1H3C/HIST1H4H/HIST1H3G/HIST1H3B/HIST1H3H/HIST1H3A/HIST1H4D/HIST1H3J/HIST1H4A/HIST2H3D/HIST1H3D/HIST1H3F | | |  |
| R-HSA-1266695 | | Interleukin-7 signaling | | -2.914103858 | | | 0.001002937 | HIST2H3C/HIST1H3E/IRS1/HIST1H3C/HIST1H3G/HIST1H3B/HIST1H3H/HIST1H3A/HIST1H3J/HIST2H3D/HIST1H3D/HIST1H3F | | |  |
| R-HSA-157118 | | Signaling by NOTCH | | -3.619209689 | | | 0.001002937 | HIST2H2BE/NEURL1B/HIST1H2BK/HIST2H3C/HIST1H3E/PSME2/HIST1H4B/HIST2H2AA3/HIST1H2BD/HIST1H2BJ/HIST1H2BC/HIST1H2BG/HIST1H4E/HIST2H4A/HES1/HIST1H4J/HIST1H4L/DLGAP5/HIST1H4C/HIST1H3C/HIST1H4H/HIST1H2AB/HIST1H2BB/TLE1/HIST1H2AD/HIST1H3G/HIST1H3B/HIST1H3H/HIST1H2BO/HIST1H2BN/HIST1H2BF/HIST1H2BM/HIST1H2AE/HIST1H3A/HIST1H2BL/HIST1H2BI/HIST3H2BB/HIST1H4D/HIST1H3J/HIST1H2BH/HIST1H4A/HIST1H2AJ/HIST2H3D/HIST1H2BE/HIST1H3D/HIST1H3F | | |  |
| R-HSA-5689880 | | Ub-specific processing proteases | | -3.204260263 | | | 0.001002937 | HIST1H2AG/HIST2H2BE/HIST1H2BK/HIST2H2BF/PSME2/HIST2H2AA3/CCNA2/HIST1H2BD/HIST1H2BJ/HIST1H2BC/HIST1H2BG/HIST1H2AL/HIST2H2AB/HIST1H2AK/HIST1H2AB/HIST1H2BB/HIST1H2AD/HIST1H2AM/HIST1H2AI/HIST1H2AH/HIST1H2BO/HIST1H2BN/HIST1H2BF/HIST1H2BM/HIST1H2AE/AXIN2/HIST1H2BL/HIST1H2BI/HIST3H2BB/HIST1H2BH/HIST1H2AJ/HIST1H2BE/IL33/RNF128 | | |  |
| R-HSA-8866654 | | E3 ubiquitin ligases ubiquitinate target proteins | | -2.936158534 | | | 0.001002937 | RNF144A/PCNA/HIST1H2BD/HIST1H2BJ/HIST1H2BC/HIST1H2BG/HIST1H2BB/HIST1H2BO/HIST1H2BN/HIST1H2BF/HIST1H2BM/HIST1H2BL/HIST1H2BI/HIST1H2BH/HIST1H2BE | | |  |
| R-HSA-73886 | | Chromosome Maintenance | | -3.035640083 | | | 0.001002937 | POLA1/HIST1H4F/NHP2/HIST1H2AC/HIST4H4/HIST2H2BE/HIST1H2BK/PCNA/CENPW/CENPU/DNA2/HIST1H4B/RPA3/HIST2H2AA3/HIST1H2BD/HIST1H2BJ/HIST1H2BC/HIST1H2BG/HIST1H4E/KNL1/HIST2H4A/HIST1H4J/HIST1H4L/CENPH/HIST1H4C/HIST1H4H/HIST1H2AB/HIST1H2BB/HIST1H2AD/HIST1H2BO/HIST1H2BN/HIST1H2BF/HIST1H2BM/HIST1H2AE/HIST1H2BL/HIST1H2BI/HIST3H2BB/HIST1H4D/HIST1H2BH/POLA2/HIST1H4A/HIST1H2AJ/HIST1H2BE | | |  |
| R-HSA-2559583 | | Cellular Senescence | | -3.822770688 | | | 0.001002937 | LMNB1/HIST2H2BE/HIST1H2BK/HIST2H3C/HIST1H3E/HIST1H4B/HIST2H2AA3/CCNA2/RPS6KA1/HIST1H2BD/HIST1H2BJ/HIST1H2BC/HIST1H2BG/HIST1H4E/HIST2H4A/HIST1H4J/HIST1H4L/HIST1H4C/HIST1H3C/HIST1H4H/HIST1H2AB/HIST1H2BB/HIST1H2AD/HIST1H1D/HIST1H3G/HIST1H3B/HIST1H3H/HIST1H1B/HIST1H2BO/HIST1H2BN/HIST1H2BF/HIST1H2BM/HIST1H2AE/HIST1H3A/HIST1H2BL/HIST1H2BI/HIST3H2BB/HIST1H4D/HIST1H3J/HIST1H2BH/HIST1H4A/HIST1H2AJ/HIST2H3D/HIST1H2BE/HIST1H3D/HIST1H3F | | |  |
| R-HSA-8939211 | | ESR-mediated signaling | | -3.470177571 | | | 0.001002937 | HIST2H2BE/HIST1H2BK/HIST2H3C/HIST1H3E/HIST1H4B/HIST2H2AA3/HIST1H2BD/HIST1H2BJ/HIST1H2BC/HIST1H2BG/HIST1H4E/HIST2H4A/HIST1H4J/HIST1H4L/HIST1H4C/HIST1H3C/HIST1H4H/HIST1H2AB/FKBP5/HIST1H2BB/HIST1H2AD/HIST1H3G/HIST1H3B/HIST1H3H/HIST1H2BO/HIST1H2BN/HIST1H2BF/HIST1H2BM/HIST1H2AE/HIST1H3A/HIST1H2BL/HIST1H2BI/HIST3H2BB/HIST1H4D/HIST1H3J/HIST1H2BH/HIST1H4A/HIST1H2AJ/HIST2H3D/HIST1H2BE/HIST1H3D/HIST1H3F | | |  |
| R-HSA-201681 | | TCF dependent signaling in response to WNT | | -3.573479247 | | | 0.001002937 | HIST2H2BE/HIST1H2BK/HIST2H3C/HIST1H3E/PSME2/HIST1H4B/HIST2H2AA3/HIST1H2BD/HIST1H2BJ/HIST1H2BC/HIST1H2BG/HIST1H4E/HIST2H4A/HIST1H4J/HIST1H4L/HIST1H4C/HIST1H3C/HIST1H4H/HIST1H2AB/HIST1H2BB/TLE1/LEF1/HIST1H2AD/HIST1H3G/HIST1H3B/HIST1H3H/HIST1H2BO/HIST1H2BN/HIST1H2BF/HIST1H2BM/HIST1H2AE/AXIN2/HIST1H3A/HIST1H2BL/HIST1H2BI/HIST3H2BB/HIST1H4D/HIST1H3J/HIST1H2BH/HIST1H4A/HIST1H2AJ/HIST2H3D/HIST1H2BE/HIST1H3D/HIST1H3F | | |  |
| R-HSA-9006931 | | Signaling by Nuclear Receptors | | -3.284977668 | | | 0.001002937 | HIST2H2BE/HIST1H2BK/HIST2H3C/HIST1H3E/RDH10/HIST1H4B/HIST2H2AA3/HIST1H2BD/HIST1H2BJ/HIST1H2BC/HIST1H2BG/HIST1H4E/HIST2H4A/HIST1H4J/HIST1H4L/HIST1H4C/HIST1H3C/HIST1H4H/HIST1H2AB/FKBP5/HIST1H2BB/HIST1H2AD/ALDH1A3/HIST1H3G/HIST1H3B/HIST1H3H/HIST1H2BO/HIST1H2BN/HIST1H2BF/HIST1H2BM/HIST1H2AE/HIST1H3A/HIST1H2BL/HIST1H2BI/HIST3H2BB/HIST1H4D/HIST1H3J/HIST1H2BH/HIST1H4A/HIST1H2AJ/HIST2H3D/HIST1H2BE/HIST1H3D/HIST1H3F | | |  |
| R-HSA-195721 | | Signaling by WNT | | -3.318212802 | | | 0.001002937 | HIST1H4B/HIST2H2AA3/HIST1H2BD/HIST1H2BJ/HIST1H2BC/HIST1H2BG/RAC3/HIST1H4E/HIST2H4A/HIST1H4J/HIST1H4L/HIST1H4C/HIST1H3C/HIST1H4H/HIST1H2AB/HIST1H2BB/TLE1/LEF1/HIST1H2AD/HIST1H3G/HIST1H3B/HIST1H3H/HIST1H2BO/HIST1H2BN/HIST1H2BF/HIST1H2BM/HIST1H2AE/AXIN2/HIST1H3A/HIST1H2BL/HIST1H2BI/HIST3H2BB/HIST1H4D/HIST1H3J/HIST1H2BH/HIST1H4A/HIST1H2AJ/HIST2H3D/HIST1H2BE/HIST1H3D/HIST1H3F | | |  |
| R-HSA-195258 | | RHO GTPase Effectors | | -3.402503187 | | | 0.001002937 | HIST2H2AA4/PRC1/H2AFV/CENPP/HIST2H2AC/SGO1/CALM3/IQGAP3/NUP85/BIRC5/TUBA1C/H2AFZ/CENPF/BRK1/BUB1B/BUB1/SPC24/SKA1/HIST1H4F/HIST1H2AC/ZW10/KIF14/HIST4H4/NUP37/NUF2/ZWINT/CDCA8/SPC25/HIST1H3I/HIST2H2BE/HIST1H2BK/HIST2H3C/LIN7B/DIAPH3/RHPN2/MAD2L1/CENPE/HIST1H3E/CENPU/HIST1H4B/HIST2H2AA3/CDC25C/HIST1H2BD/HIST1H2BJ/HIST1H2BC/HIST1H2BG/NDC80/HIST1H4E/KNL1/HIST2H4A/KIF2C/HIST1H4J/HIST1H4L/CENPH/HIST1H4C/HIST1H3C/HIST1H4H/HIST1H2AB/HIST1H2BB/HIST1H2AD/HIST1H3G/HIST1H3B/HIST1H3H/HIST1H2BO/HIST1H2BN/HIST1H2BF/HIST1H2BM/HIST1H2AE/HIST1H3A/HIST1H2BL/HIST1H2BI/HIST3H2BB/HIST1H4D/HIST1H3J/HIST1H2BH/HIST1H4A/HIST1H2AJ/HIST2H3D/HIST1H2BE/HIST1H3D/HIST1H3F | | |  |
| R-HSA-2262752 | | Cellular responses to stress | | -3.153471793 | | | 0.001002937 | HIST2H2AA4/NUP188/TPR/H2AFV/PSMB9/HIST2H2AC/NDC1/CAT/NUP85/PSMD1/PSMA5/TUBA1C/PSMB8/UBE2C/PSMA3/H2AFZ/HIST1H1C/PSMB1/CDKN2C/HIST1H4F/HIST1H2AC/HIST4H4/NUP37/CCNE2/HIGD1A/CYCS/HIST1H3I/LMNB1/HIST2H2BE/HIST1H2BK/HIST2H3C/DCTN3/HSPA14/HIST1H3E/PSME2/HIST1H4B/RPA3/HIST2H2AA3/CCNA2/RPS6KA1/HIST1H2BD/HIST1H2BJ/HIST1H2BC/HIST1H2BG/HIST1H4E/HIST2H4A/HIST1H4J/HIST1H4L/HIST1H4C/HIST1H3C/HIST1H4H/HIST1H2AB/FKBP5/HIST1H2BB/HIST1H2AD/HIST1H1D/HIST1H3G/HIST1H3B/HIST1H3H/HIST1H1B/HIST1H2BO/HIST1H2BN/HIST1H2BF/HIST1H2BM/HIST1H2AE/HIST1H3A/HIST1H2BL/HIST1H2BI/HIST3H2BB/HIST1H4D/CAMK2B/HIST1H3J/HIST1H2BH/HIST1H4A/HIST1H2AJ/HIST2H3D/HIST1H2BE/HIST1H3D/HIST1H3F | | |  |
| R-HSA-69620 | | Cell Cycle Checkpoints | | -2.618603805 | | | 0.001002937 | CENPP/PSMB9/SGO1/DBF4/NUP85/BIRC5/PSMD1/PSMA5/PSMB8/UBE2C/PSMA3/BRCA1/CENPF/BUB1B/BUB1/CCNB1/SPC24/PSMB1/SKA1/RFC5/HIST1H4F/ZW10/HIST4H4/NUP37/CCNE2/NUF2/ZWINT/CDCA8/SPC25/HIST2H2BE/HIST1H2BK/BARD1/MAD2L1/CENPE/CCNB2/CENPU/DNA2/PSME2/HIST1H4B/RPA3/CCNA2/CDC25C/HIST1H2BD/HIST1H2BJ/HIST1H2BC/HIST1H2BG/NDC80/HIST1H4E/KNL1/HIST2H4A/KIF2C/HIST1H4J/CDK1/HIST1H4L/CENPH/HIST1H4C/HIST1H4H/HIST1H2BB/HIST1H2BO/HIST1H2BN/HIST1H2BF/HIST1H2BM/HIST1H2BL/HIST1H2BI/HIST3H2BB/HIST1H4D/HIST1H2BH/HIST1H4A/HIST1H2BE | | |  |
| R-HSA-73894 | | DNA Repair | | -2.423538319 | | | 0.001002937 | USP1/TDG/POLE2/NEIL3/RHNO1/NBN/LOC102724334/SUMO3/KPNA2/RBBP8/RAD51/DDB1/HMGN1/FANCI/RMI2/EME1/XRCC4/HIST2H2AA4/XRCC2/RFC3/H2AFV/LIG1/HIST2H2AC/GEN1/POLR2G/FANCD2/H2AFZ/POLE4/BRCA1/COPS3/MAD2L2/RFC5/HIST1H4F/HIST1H2AC/HIST4H4/RAD51AP1/FANCG/HIST2H2BE/HIST1H2BK/BARD1/PCNA/PCLAF/DNA2/MSH2/UBE2T/HIST1H4B/RPA3/HIST2H2AA3/CCNA2/HIST1H2BD/HIST1H2BJ/HIST1H2BC/HIST1H2BG/HIST1H4E/HIST2H4A/HIST1H4J/HIST1H4L/HIST1H4C/HIST1H4H/HIST1H2AB/HIST1H2BB/HIST1H2AD/HIST1H2BO/HIST1H2BN/HIST1H2BF/HIST1H2BM/HIST1H2AE/HIST1H2BL/HIST1H2BI/HIST3H2BB/HIST1H4D/HIST1H2BH/HIST1H4A/HIST1H2AJ/HIST1H2BE/SLX1B | | |  |
| R-HSA-194315 | | Signaling by Rho GTPases | | -3.3436575 | | | 0.001002937 | HIST1H2AC/ZW10/KIF14/ARHGEF40/HIST4H4/NUP37/ARHGAP20/NUF2/ZWINT/CDCA8/SPC25/RACGAP1/HIST1H3I/HIST2H2BE/HIST1H2BK/HIST2H3C/LIN7B/DEPDC1B/DIAPH3/RHPN2/MAD2L1/CENPE/HIST1H3E/CENPU/HIST1H4B/HIST2H2AA3/CDC25C/HIST1H2BD/HIST1H2BJ/HIST1H2BC/HIST1H2BG/RAC3/NDC80/HIST1H4E/KNL1/HIST2H4A/KIF2C/HIST1H4J/ARHGEF6/HIST1H4L/CENPH/HIST1H4C/HIST1H3C/HIST1H4H/HIST1H2AB/HIST1H2BB/HIST1H2AD/HIST1H3G/HIST1H3B/HIST1H3H/HIST1H2BO/HIST1H2BN/HIST1H2BF/HIST1H2BM/HIST1H2AE/HIST1H3A/HIST1H2BL/HIST1H2BI/HIST3H2BB/HIST1H4D/HIST1H3J/HIST1H2BH/HIST1H4A/HIST1H2AJ/HIST2H3D/HIST1H2BE/ARHGDIG/HIST1H3D/HIST1H3F | | |  |
| R-HSA-8953897 | | Cellular responses to external stimuli | | -2.821196956 | | | 0.001002937 | HIST2H2AA4/NUP188/TPR/H2AFV/PSMB9/HIST2H2AC/NDC1/CAT/NUP85/PSMD1/PSMA5/TUBA1C/PSMB8/UBE2C/PSMA3/H2AFZ/HIST1H1C/ATG12/PSMB1/CDKN2C/HIST1H4F/HIST1H2AC/HIST4H4/TOMM5/NUP37/CCNE2/HIGD1A/CYCS/HIST1H3I/LMNB1/HIST2H2BE/HIST1H2BK/HIST2H3C/DCTN3/HSPA14/HIST1H3E/PSME2/HIST1H4B/RPA3/HIST2H2AA3/CCNA2/RPS6KA1/HIST1H2BD/HIST1H2BJ/HIST1H2BC/HIST1H2BG/HIST1H4E/HIST2H4A/HIST1H4J/HIST1H4L/HIST1H4C/HIST1H3C/HIST1H4H/HIST1H2AB/FKBP5/HIST1H2BB/HIST1H2AD/HIST1H1D/HIST1H3G/HIST1H3B/HIST1H3H/HIST1H1B/HIST1H2BO/HIST1H2BN/HIST1H2BF/HIST1H2BM/HIST1H2AE/HIST1H3A/HIST1H2BL/HIST1H2BI/HIST3H2BB/HIST1H4D/CAMK2B/HIST1H3J/HIST1H2BH/HIST1H4A/HIST1H2AJ/HIST2H3D/HIST1H2BE/HIST1H3D/HIST1H3F | | |  |
| R-HSA-68886 | | M Phase | | -3.537371803 | | | 0.001002937 | HIST2H2AA4/NUP188/NCAPG2/TPR/H2AFV/CENPP/PSMB9/HIST2H2AC/SGO1/NDC1/NCAPH2/NUP85/BIRC5/PSMD1/PSMA5/TUBA1C/PSMB8/UBE2C/PSMA3/H2AFZ/CENPF/BUB1B/BUB1/CCNB1/SPC24/PSMB1/NEDD1/SKA1/KIF20A/HIST1H4F/HIST1H2AC/ZW10/HIST4H4/NUP37/NUF2/PLK4/HAUS2/ZWINT/CDCA8/SPC25/SMC2/HIST1H3I/LMNB1/HIST2H2BE/NCAPH/HIST1H2BK/HIST2H3C/DCTN3/NEK2/MZT1/MAD2L1/CENPE/HIST1H3E/CCNB2/KIF23/CENPU/PSME2/HIST1H4B/HIST2H2AA3/PTTG1/HIST1H2BD/HIST1H2BJ/HIST1H2BC/HIST1H2BG/PRKAR2B/NCAPG/NDC80/CNTRL/HIST1H4E/KNL1/HIST2H4A/KIF2C/HIST1H4J/CDK1/HIST1H4L/CENPH/HIST1H4C/HIST1H3C/HIST1H4H/HIST1H2AB/HIST1H2BB/HIST1H2AD/HIST1H3G/HIST1H3B/HIST1H3H/HIST1H2BO/HIST1H2BN/HIST1H2BF/HIST1H2BM/HIST1H2AE/HIST1H3A/HIST1H2BL/HIST1H2BI/HIST3H2BB/HIST1H4D/HIST1H3J/HIST1H2BH/HIST1H4A/HIST1H2AJ/HIST2H3D/HIST1H2BE/HIST1H3D/HIST1H3F | | |  |
| R-HSA-597592 | | Post-translational protein modification | | -2.199763745 | | | 0.001002937 | XRCC4/HIST2H2AA4/NUP188/TPR/TAF9B/SAE1/NSMCE4A/GALNT7/PSMB9/HIST2H2AC/CALM3/NDC1/COMMD4/NUP85/MGAT2/RAB27B/BIRC5/MANEA/PSMD1/PSMA5/TUBA1C/PSMB8/TTLL7/BET1/UBE2C/PSMA3/THSD1/HLA-A/PIGX/BRCA1/NSMCE2/SRD5A3/COPS3/ALG10/B3GNT5/PSMB1/BTBD1/VNN1/RAB7B/LRRC49/HIST1H4F/HIST1H2AC/C1GALT1/HIST4H4/RAB23/NUP37/RAB31/BIRC3/RAB8B/CDCA8/SKP2/HIST1H2AG/FBXO4/HIST2H2BE/HIST1H2BK/TRAPPC2L/IGFBP3/BARD1/RNF144A/DCTN3/ASB13/TOP2A/PCNA/LYPD1/HIST2H2BF/PSME2/RAB40B/UBE2T/HIST1H4B/RAB38/GALNT6/HIST2H2AA3/CCNA2/GCNT4/GALNT12/COPZ2/GALNT13/HIST1H2BD/HIST1H2BJ/HIST1H2BC/HIST1H2BG/HIST1H4E/HIST2H4A/HIST1H4J/CDK1/HIST1H4L/HIST1H2AL/DNMT3B/HIST1H4C/HIST1H4H/HIST2H2AB/HIST1H2AK/HIST1H2AB/HIST1H2BB/HIST1H2AD/HIST1H2AM/HIST1H2AI/PIGZ/HIST1H2AH/HIST1H2BO/ETFB/HIST1H2BN/HIST1H2BF/HIST1H2BM/HIST1H2AE/AXIN2/HIST1H2BL/HIST1H2BI/HIST3H2BB/HIST1H4D/HIST1H2BH/HIST1H4A/HIST1H2AJ/GCNT1/HIST1H2BE/SPTBN4/IL33/RNF128/GALNT5 | | |  |
| R-HSA-1640170 | | Cell Cycle | | -3.128897558 | | | 0.001002937 | HIST2H2AA4/NUP188/NCAPG2/TPR/RFC3/H2AFV/LIG1/CENPP/PSMB9/HIST2H2AC/SGO1/DBF4/NDC1/NCAPH2/NUP85/BIRC5/TK1/PSMD1/PSMA5/TUBA1C/PSMB8/UBE2C/PSMA3/H2AFZ/POLE4/BRCA1/CENPF/BUB1B/BUB1/CCNB1/SPC24/PSMB1/NEDD1/GMNN/SKA1/RFC5/KIF20A/CDKN2C/POLA1/HIST1H4F/NHP2/HIST1H2AC/ZW10/HIST4H4/NUP37/GINS4/CCNE2/NUF2/PLK4/HAUS2/ZWINT/CDCA8/SPC25/SMC2/SKP2/HIST1H3I/LMNB1/HIST2H2BE/NCAPH/HIST1H2BK/HIST2H3C/BARD1/DCTN3/NEK2/TOP2A/PCNA/MZT1/MAD2L1/CENPE/HIST1H3E/CCNB2/KIF23/CENPW/CENPU/DNA2/PSME2/HIST1H4B/RPA3/HIST2H2AA3/CCNA2/PTTG1/CDC25C/HIST1H2BD/HIST1H2BJ/HIST1H2BC/HMMR/GINS1/BORA/HIST1H2BG/PRKAR2B/NCAPG/DHFR/NDC80/CNTRL/HIST1H4E/KNL1/HIST2H4A/KIF2C/HIST1H4J/CDK1/HIST1H4L/CENPH/HIST1H4C/ESCO2/HIST1H3C/HIST1H4H/HIST1H2AB/HIST1H2BB/HIST1H2AD/HIST1H3G/HIST1H3B/HIST1H3H/HIST1H2BO/HIST1H2BN/HIST1H2BF/HIST1H2BM/HIST1H2AE/HIST1H3A/HIST1H2BL/HIST1H2BI/HIST3H2BB/HIST1H4D/HIST1H3J/HIST1H2BH/POLA2/HIST1H4A/HIST1H2AJ/HIST2H3D/HIST1H2BE/HIST1H3D/HIST1H3F | | |  |
| R-HSA-212436 | | Generic Transcription Pathway | | -2.455102968 | | | 0.001002937 | HIST1H3I/SESN3/HIST2H2BE/HIST1H2BK/HIST2H3C/IGFBP3/BARD1/PCNA/E2F8/HIST1H3E/DNA2/MEF2C/MSH2/PSME2/HIST1H4B/RPA3/HIST2H2AA3/CCNA2/CDC25C/HIST1H2BD/HIST1H2BJ/HIST1H2BC/HIST1H2BG/HIST1H4E/HIST2H4A/HES1/TRPC3/HIST1H4J/CDK1/HIST1H4L/MAF/HIST1H4C/HIST1H3C/HIST1H4H/HIST1H2AB/FKBP5/HIST1H2BB/LEF1/HIST1H2AD/HIST1H3G/HIST1H3B/HIST1H3H/HIST1H2BO/HIST1H2BN/HIST1H2BF/HIST1H2BM/HIST1H2AE/HIST1H3A/HIST1H2BL/HIST1H2BI/TXNIP/FOXO1/HIST3H2BB/HIST1H4D/CAMK2B/HIST1H3J/HIST1H2BH/HIST1H4A/HIST1H2AJ/HIST2H3D/HIST1H2BE/HIST1H3D/HIST1H3F | | |  |
| R-HSA-1266738 | | Developmental Biology | | -2.406560333 | | | 0.001002937 | SCN1B/PSME2/ADAM11/HIST1H4B/HIST2H2AA3/ACVR1C/UNC5B/RPS6KA1/HIST1H2BD/HIST1H2BJ/HIST1H2BC/HIST1H2BG/HIST1H4E/HIST2H4A/HES1/TRPC3/HIST1H4J/HIST1H4L/HIST1H4C/HIST1H3C/HIST1H4H/HIST1H2AB/HIST1H2BB/HIST1H2AD/HIST1H3G/COL9A2/HIST1H3B/HIST1H3H/VLDLR/HIST1H2BO/HIST1H2BN/HIST1H2BF/HIST1H2BM/HIST1H2AE/HIST1H3A/HIST1H2BL/HIST1H2BI/FOXO1/HIST3H2BB/HIST1H4D/HIST1H3J/HIST1H2BH/HIST1H4A/HIST1H2AJ/HIST2H3D/HIST1H2BE/HIST1H3D/SPTBN4/PAK6/KLK5/HIST1H3F | | |  |
| R-HSA-73857 | | RNA Polymerase II Transcription | | -2.410403877 | | | 0.001002937 | HIST1H3I/SESN3/HIST2H2BE/HIST1H2BK/HIST2H3C/IGFBP3/BARD1/NUDT21/SNRPB/PCNA/E2F8/HIST1H3E/DNA2/MEF2C/MSH2/PSME2/HIST1H4B/RPA3/HIST2H2AA3/CCNA2/CDC25C/HIST1H2BD/HIST1H2BJ/HIST1H2BC/HIST1H2BG/HIST1H4E/HIST2H4A/HES1/TRPC3/HIST1H4J/CDK1/HIST1H4L/MAF/HIST1H4C/HIST1H3C/HIST1H4H/HIST1H2AB/FKBP5/HIST1H2BB/LEF1/HIST1H2AD/HIST1H3G/HIST1H3B/HIST1H3H/HIST1H2BO/HIST1H2BN/HIST1H2BF/HIST1H2BM/HIST1H2AE/HIST1H3A/HIST1H2BL/HIST1H2BI/TXNIP/FOXO1/HIST3H2BB/HIST1H4D/CAMK2B/HIST1H3J/HIST1H2BH/HIST1H4A/HIST1H2AJ/HIST2H3D/HIST1H2BE/HIST1H3D/HIST1H3F | | |  |
| R-HSA-74160 | | Gene expression (Transcription) | | -2.194787534 | | | 0.001002937 | HIST1H3I/SESN3/HIST2H2BE/HIST1H2BK/HIST2H3C/IGFBP3/BARD1/NUDT21/SNRPB/PCNA/E2F8/HIST1H3E/DNA2/MEF2C/MSH2/PSME2/HIST1H4B/RPA3/HIST2H2AA3/CCNA2/CDC25C/HIST1H2BD/HIST1H2BJ/HIST1H2BC/HIST1H2BG/HIST1H4E/HIST2H4A/HES1/TRPC3/HIST1H4J/CDK1/HIST1H4L/MAF/DNMT3B/HIST1H4C/HIST1H3C/HIST1H4H/HIST1H2AB/FKBP5/HIST1H2BB/LEF1/HIST1H2AD/HIST1H3G/HIST1H3B/HIST1H3H/HIST1H2BO/HIST1H2BN/HIST1H2BF/HIST1H2BM/HIST1H2AE/HIST1H3A/HIST1H2BL/HIST1H2BI/TXNIP/FOXO1/HIST3H2BB/HIST1H4D/CAMK2B/HIST1H3J/HIST1H2BH/HIST1H4A/HIST1H2AJ/HIST2H3D/HIST1H2BE/HIST1H3D/HIST1H3F | | |  |
| R-HSA-69278 | | Cell Cycle. Mitotic | | -3.453251439 | | | 0.001002937 | HIST2H2AA4/NUP188/NCAPG2/TPR/RFC3/H2AFV/LIG1/CENPP/PSMB9/HIST2H2AC/SGO1/DBF4/NDC1/NCAPH2/NUP85/BIRC5/TK1/PSMD1/PSMA5/TUBA1C/PSMB8/UBE2C/PSMA3/H2AFZ/POLE4/CENPF/BUB1B/BUB1/CCNB1/SPC24/PSMB1/NEDD1/GMNN/SKA1/RFC5/KIF20A/CDKN2C/POLA1/HIST1H4F/HIST1H2AC/ZW10/HIST4H4/NUP37/GINS4/CCNE2/NUF2/PLK4/HAUS2/ZWINT/CDCA8/SPC25/SMC2/SKP2/HIST1H3I/LMNB1/HIST2H2BE/NCAPH/HIST1H2BK/HIST2H3C/DCTN3/NEK2/TOP2A/PCNA/MZT1/MAD2L1/CENPE/HIST1H3E/CCNB2/KIF23/CENPU/DNA2/PSME2/HIST1H4B/RPA3/HIST2H2AA3/CCNA2/PTTG1/CDC25C/HIST1H2BD/HIST1H2BJ/HIST1H2BC/HMMR/GINS1/BORA/HIST1H2BG/PRKAR2B/NCAPG/DHFR/NDC80/CNTRL/HIST1H4E/KNL1/HIST2H4A/KIF2C/HIST1H4J/CDK1/HIST1H4L/CENPH/HIST1H4C/ESCO2/HIST1H3C/HIST1H4H/HIST1H2AB/HIST1H2BB/HIST1H2AD/HIST1H3G/HIST1H3B/HIST1H3H/HIST1H2BO/HIST1H2BN/HIST1H2BF/HIST1H2BM/HIST1H2AE/HIST1H3A/HIST1H2BL/HIST1H2BI/HIST3H2BB/HIST1H4D/HIST1H3J/HIST1H2BH/POLA2/HIST1H4A/HIST1H2AJ/HIST2H3D/HIST1H2BE/HIST1H3D/HIST1H3F | | |  |
| R-HSA-162582 | | Signal Transduction | | -2.234393285 | | | 0.001007501 | LEPR/HIST1H2AC/ZW10/KIF14/ARHGEF40/HIST4H4/NUP37/DHRS3/ARHGAP20/NUF2/BIRC3/INHBB/ZWINT/CDCA8/SPC25/RACGAP1/GPR37/HIST1H3I/HIST2H2BE/NEURL1B/HIST1H2BK/HIST2H3C/LIN7B/DEPDC1B/DIAPH3/RHPN2/SMAD9/LAMC2/MAD2L1/CENPE/HIST1H3E/CENPU/MEF2C/IRS1/RDH10/PSME2/HIST1H4B/PTPN18/HIST2H2AA3/ACVR1C/REEP1/RPS6KA1/CDC25C/HIST1H2BD/HIST1H2BJ/PTPRU/HIST1H2BC/HIST1H2BG/PRKAR2B/RAC3/NDC80/HIST1H4E/KNL1/HIST2H4A/HES1/KIF2C/TRPC3/HIST1H4J/CDK1/ARHGEF6/HIST1H4L/CENPH/DLGAP5/HIST1H4C/HIST1H3C/HIST1H4H/HIST1H2AB/FKBP5/HIST1H2BB/TLE1/LEF1/HIST1H2AD/ALDH1A3/HIST1H3G/COL9A2/HIST1H3B/HIST1H3H/KDR/S1PR1/NMU/HIST1H2BO/HIST1H2BN/HIST1H2BF/XK/HIST1H2BM/HIST1H2AE/AXIN2/HIST1H3A/HIST1H2BL/HIST1H2BI/FOXO1/HIST3H2BB/SH3GL3/HIST1H4D/ADCYAP1/CAMK2B/HIST1H3J/HIST1H2BH/HIST1H4A/HIST1H2AJ/FLT3/HIST2H3D/HIST1H2BE/ARHGDIG/LINGO1/APLN/FGFR3/HIST1H3D/GREM2/SPTBN4/CGN/IL33/HIST1H3F | | |  |
| R-HSA-983231 | | Factors involved in megakaryocyte development and platelet production | | -2.470483825 | | | 0.001839302 | KIF20A/KIF4A/RACGAP1/KIF20B/HIST1H3I/HIST2H3C/CENPE/HIST1H3E/KIF23/KIF11/PRKAR2B/KIF2C/HIST1H3C/HIST1H3G/HIST1H3B/HIST1H3H/HIST1H3A/HIST1H3J/HIST2H3D/HIST1H3D/HIST1H3F | | |  |
| R-HSA-449147 | | Signaling by Interleukins | | -2.342951323 | | | 0.001839302 | MEF2C/IRS1/PSME2/PTPN18/RPS6KA1/IL21R/HIST1H3C/HIST1H3G/HIST1H3B/HIST1H3H/S1PR1/IL34/HIST1H3A/FOXO1/ITGB2/HIST1H3J/FLT3/HIST2H3D/SERPINB2/HIST1H3D/PTPRZ1/IL33/HIST1H3F | | |  |
| R-HSA-69481 | | G2/M Checkpoints | | -2.548300872 | | | 0.001839302 | HIST1H2BK/BARD1/CCNB2/DNA2/PSME2/HIST1H4B/RPA3/CDC25C/HIST1H2BD/HIST1H2BJ/HIST1H2BC/HIST1H2BG/HIST1H4E/HIST2H4A/HIST1H4J/CDK1/HIST1H4L/HIST1H4C/HIST1H4H/HIST1H2BB/HIST1H2BO/HIST1H2BN/HIST1H2BF/HIST1H2BM/HIST1H2BL/HIST1H2BI/HIST3H2BB/HIST1H4D/HIST1H2BH/HIST1H4A/HIST1H2BE | | |  |
| R-HSA-5689901 | | Metalloprotease DUBs | | -2.45244756 | | | 0.003638111 | HIST1H2AG/BARD1/HIST2H2AA3/HIST1H2AL/HIST2H2AB/HIST1H2AK/HIST1H2AB/HIST1H2AD/HIST1H2AM/HIST1H2AI/HIST1H2AH/HIST1H2AE/HIST1H2AJ | | |  |
| R-HSA-1280215 | | Cytokine Signaling in Immune system | | -2.192817153 | | | 0.003644719 | TRIM6/PTPN1/NUP188/IL4R/TPR/PSMB9/NDC1/NUP85/BIRC5/PSMD1/PSMA5/PSMB8/PSMA3/CHUK/HLA-A/TNFRSF1B/HMGB1/GSTO1/PSMB1/RELB/NUP37/BIRC3/HIST1H3I/LMNB1/TNFRSF9/HIST2H3C/HIST1H3E/MEF2C/IRS1/MID1/PSME2/PTPN18/RPS6KA1/IL21R/TNFRSF8/HIST1H3C/HIST1H3G/HIST1H3B/HIST1H3H/S1PR1/IFNAR2/HLA-F/IL34/HIST1H3A/FOXO1/TNFRSF11A/ITGB2/CAMK2B/HIST1H3J/FLT3/HIST2H3D/SERPINB2/HIST1H3D/PTPRZ1/IL33/HIST1H3F | | |  |
| R-HSA-392499 | | Metabolism of proteins | | -1.735811649 | | | 0.004736729 | NUP85/MGAT2/RAB27B/PPA1/BIRC5/MANEA/PSMD1/PSMA5/TUBA1C/PSMB8/TTLL7/BET1/UBE2C/PSMA3/H2AFZ/THSD1/HLA-A/PIGX/BRCA1/NSMCE2/MRPL39/SRD5A3/COPS3/ALG10/B3GNT5/RPL39L/PSMB1/RPS18/MME/BTBD1/VNN1/RAB7B/KARS/LRRC49/HIST1H4F/HIST1H2AC/C1GALT1/HIST4H4/RAB23/NUP37/RPL28/RAB31/CCNE2/BIRC3/INHBB/RAB8B/CDCA8/RPS29/SKP2/HIST1H3I/HIST1H2AG/FBXO4/HIST2H2BE/HIST1H2BK/HIST2H3C/TRAPPC2L/MRPL51/IGFBP3/BARD1/RNF144A/DCTN3/ASB13/TOP2A/PCNA/HIST1H3E/LYPD1/HIST2H2BF/PSME2/RAB40B/UBE2T/HIST1H4B/RAB38/GALNT6/HIST2H2AA3/CCNA2/GCNT4/CTSH/GALNT12/EXOSC8/COPZ2/GALNT13/HIST1H2BD/HIST1H2BJ/HIST1H2BC/HIST1H2BG/HIST1H4E/HIST2H4A/HIST1H4J/CDK1/HIST1H4L/HIST1H2AL/DNMT3B/HIST1H4C/HIST1H3C/HIST1H4H/HIST2H2AB/HIST1H2AK/HIST1H2AB/HIST1H2BB/HIST1H2AD/HIST1H3G/HIST1H2AM/HIST1H3B/HIST1H3H/HIST1H2AI/PIGZ/HIST1H2AH/HIST1H2BO/ETFB/HIST1H2BN/HIST1H2BF/HIST1H2BM/HIST1H2AE/AXIN2/HIST1H3A/HIST1H2BL/HIST1H2BI/HIST3H2BB/HIST1H4D/HIST1H3J/HIST1H2BH/HIST1H4A/HIST1H2AJ/HIST2H3D/GCNT1/HIST1H2BE/HIST1H3D/TSPAN33/SPTBN4/IL33/RNF128/GALNT5/HIST1H3F | | |  |
| **Supplementary Table S2: Deregulated pathways in epithelioid CD473 cells at 48h** | | | | | | | | | | | |
| **ID** | | **Pathway name** | | **NES** | **p.adjust** | | | **Genes in pathway** | | |  |
| R-HSA-194315 | | Signaling by Rho GTPases | | -2.651876178 | 0.000729402 | | | ITGB3BP/CLASP2/HIST1H3I/IQGAP3/RHOA/CDC25C/KIF14/CDC20/CDCA8/KIF18A/HIST2H2BE/ARHGAP11A/SGO2/PFN2/CENPO/HIST1H2BD/RHOBTB1/HIST1H3C/BUB1/HIST1H4B/MAD2L1/HIST1H3H/KIF2C/H2AFZ/HIST1H3E/HIST1H2BC/HIST1H3J/RHPN2/CIT/H3F3A/HIST1H4L/NDC80/CENPU/HIST1H4E/CENPH/CENPN/HIST1H2AB/BUB1B/HIST1H2BO/ARHGAP29/CENPP/PAK1/HIST1H3B/HIST1H4H/HIST1H2BJ/HIST1H3G/HIST1H4C/BIRC5/HIST1H4D/HIST2H4A/CENPF/HIST1H2AE/SPC25/HIST1H2BN/HIST1H2AD/SPDL1/DEPDC1B/HIST1H3A/HIST1H2BH/CENPE/HIST1H4F/HIST1H2BI/HIST1H2BL/CENPA/HIST1H2BF/HIST1H2BG/HIST1H2BE/NUF2/DIAPH3/HIST1H4A/HIST1H2BB/HIST3H2BB/HIST2H3D/HIST1H2AJ/CDH1/HIST1H2BM/HIST1H3D/HIST1H3F | | |  |
| R-HSA-195258 | | RHO GTPase Effectors | | -2.88745672 | 0.000729402 | | | ITGB3BP/CLASP2/HIST1H3I/IQGAP3/RHOA/CDC25C/KIF14/CDC20/CDCA8/KIF18A/HIST2H2BE/SGO2/PFN2/CENPO/HIST1H2BD/HIST1H3C/BUB1/HIST1H4B/MAD2L1/HIST1H3H/KIF2C/H2AFZ/HIST1H3E/HIST1H2BC/HIST1H3J/RHPN2/CIT/H3F3A/HIST1H4L/NDC80/CENPU/HIST1H4E/CENPH/CENPN/HIST1H2AB/BUB1B/HIST1H2BO/CENPP/PAK1/HIST1H3B/HIST1H4H/HIST1H2BJ/HIST1H3G/HIST1H4C/BIRC5/HIST1H4D/HIST2H4A/CENPF/HIST1H2AE/SPC25/HIST1H2BN/HIST1H2AD/SPDL1/HIST1H3A/HIST1H2BH/CENPE/HIST1H4F/HIST1H2BI/HIST1H2BL/CENPA/HIST1H2BF/HIST1H2BG/HIST1H2BE/NUF2/DIAPH3/HIST1H4A/HIST1H2BB/HIST3H2BB/HIST2H3D/HIST1H2AJ/CDH1/HIST1H2BM/HIST1H3D/HIST1H3F | | |  |
| R-HSA-68886 | | M Phase | | -2.819100941 | 0.000729402 | | | WAPL/KNL1/ANAPC15/HIST1H4J/HIST2H3C/NCAPH/PRKAR2B/PPP2CB/HAUS2/PTTG1/AHCTF1/HIST2H2AC/ITGB3BP/SMC2/CLASP2/HIST1H3I/NUP205/CDC20/CDCA8/KIF18A/CDCA5/HIST2H2BE/MASTL/SGO2/CENPO/HIST1H2BD/TMPO/HIST1H3C/BUB1/HIST1H4B/MAD2L1/HIST1H3H/NCAPG2/KIF2C/H2AFZ/LMNB1/HIST1H3E/HIST1H2BC/HIST1H3J/NEK2/CCNB2/UBE2C/KIF23/H3F3A/NUP35/HIST1H4L/NDC80/CCNB1/CENPU/HIST1H4E/CDK1/CENPH/CENPN/HIST1H2AB/BUB1B/HIST1H2BO/FBXO5/CENPP/HIST1H3B/HIST1H4H/HIST1H2BJ/HIST1H3G/HIST1H4C/BIRC5/HIST1H4D/HIST2H4A/CENPF/HAUS6/HIST1H2AE/KIF20A/SPC25/HIST1H2BN/HIST1H2AD/SPDL1/HIST1H3A/HIST1H2BH/CENPE/HIST1H4F/HIST1H2BI/HIST1H2BL/CENPA/HIST1H2BF/HIST1H2BG/HIST1H2BE/NUF2/HIST1H4A/HIST1H2BB/HIST3H2BB/HIST2H3D/HIST1H2AJ/HIST1H2BM/HIST1H3D/HIST1H3F | | |  |
| R-HSA-69620 | | Cell Cycle Checkpoints | | -2.510896928 | 0.000729402 | | | ITGB3BP/CCNE2/CLASP2/CDC25C/CDC20/CDCA8/KIF18A/HIST2H2BE/SGO2/CENPO/HIST1H2BD/BUB1/HIST1H4B/GTSE1/CDC7/MAD2L1/KIF2C/HIST1H2BC/RMI1/CCNB2/UBE2C/HIST1H4L/BARD1/NDC80/CCNB1/CENPU/DBF4/HIST1H4E/CDK1/CENPH/CENPN/BUB1B/HIST1H2BO/CENPP/WEE1/HIST1H4H/CCNA2/ORC1/HIST1H2BJ/HIST1H4C/BIRC5/HIST1H4D/HIST2H4A/PKMYT1/CENPF/SPC25/HIST1H2BN/RAD1/RFC3/SPDL1/HIST1H2BH/CENPE/HIST1H4F/HIST1H2BI/HIST1H2BL/CENPA/HIST1H2BF/HIST1H2BG/HIST1H2BE/NUF2/HIST1H4A/HIST1H2BB/HIST3H2BB/HIST1H2BM | | |  |
| R-HSA-157118 | | Signaling by NOTCH | | -2.573523684 | 0.000729402 | | | HES1/HIST1H3C/HIST1H4B/HIST1H3H/H2AFZ/HIST1H3E/HIST1H2BC/HIST1H3J/H3F3A/HIST1H4L/HIST1H4E/HIST1H2AB/HIST1H2BO/TLE1/HIST1H3B/HIST1H4H/HIST1H2BJ/HIST1H3G/HIST1H4C/HIST1H4D/HIST2H4A/HIST1H2AE/DLGAP5/HIST1H2BN/HIST1H2AD/HIST1H3A/HIST1H2BH/HIST1H4F/HIST1H2BI/HIST1H2BL/HIST1H2BF/HIST1H2BG/HIST1H2BE/HIST1H4A/HIST1H2BB/HIST3H2BB/HIST2H3D/HIST1H2AJ/HIST1H2BM/HIST1H3D/HIST1H3F | | |  |
| R-HSA-2559582 | | Senescence-Associated Secretory Phenotype (SASP) | | -2.602934319 | 0.000729402 | | | HIST1H3C/HIST1H4B/HIST1H3H/H2AFZ/HIST1H3E/HIST1H2BC/HIST1H3J/UBE2C/CDKN2C/H3F3A/HIST1H4L/HIST1H4E/HIST1H2AB/HIST1H2BO/HIST1H3B/HIST1H4H/CCNA2/HIST1H2BJ/HIST1H3G/HIST1H4C/HIST1H4D/HIST2H4A/HIST1H2AE/HIST1H2BN/HIST1H2AD/HIST1H3A/HIST1H2BH/HIST1H4F/HIST1H2BI/HIST1H2BL/HIST1H2BF/HIST1H2BG/HIST1H2BE/HIST1H4A/HIST1H2BB/HIST3H2BB/HIST2H3D/HIST1H2AJ/HIST1H2BM/HIST1H3D/HIST1H3F | | |  |
| R-HSA-9018519 | | Estrogen-dependent gene expression | | -2.709533577 | 0.000729402 | | | HIST1H3C/HIST1H4B/NRIP1/HIST1H3H/H2AFZ/HIST1H3E/HIST1H2BC/HIST1H3J/H3F3A/HIST1H4L/KPNA2/HIST1H4E/HIST1H2AB/GTF2F2/HIST1H2BO/HIST1H3B/HIST1H4H/HIST1H2BJ/HIST1H3G/HIST1H4C/HIST1H4D/HIST2H4A/HIST1H2AE/HIST1H2BN/HIST1H2AD/HIST1H3A/HIST1H2BH/HIST1H4F/HIST1H2BI/HIST1H2BL/HIST1H2BF/HIST1H2BG/HIST1H2BE/HIST1H4A/HIST1H2BB/HIST3H2BB/HIST2H3D/HIST1H2AJ/HIST1H2BM/HIST1H3D/ERBB4/HIST1H3F | | |  |
| R-HSA-3214815 | | HDACs deacetylate histones | | -2.776303747 | 0.000729402 | | | HIST1H2BD/HIST1H3C/HIST1H4B/HIST1H3H/HIST1H3E/HIST1H2BC/HIST1H3J/HIST1H2AL/HIST1H4L/HIST1H4E/HIST1H2AM/HIST1H2AB/HIST1H2BO/HIST1H3B/HIST1H4H/HIST1H2BJ/HIST1H3G/HIST1H4C/HIST1H4D/HIST2H4A/HIST1H2AE/HIST1H2BN/HIST1H2AD/HIST2H2BF/HIST1H2AH/HIST1H3A/HIST1H2AI/HIST1H2BH/HIST1H4F/HIST1H2BI/HIST1H2BL/HIST1H2BF/HIST1H2BG/HIST1H2BE/HIST1H4A/HIST1H2BB/HIST3H2BB/HIST2H3D/HIST1H2AJ/HIST1H2AK/HIST1H2BM/HIST1H3D/HIST1H3F | | |  |
| R-HSA-1474165 | | Reproduction | | -2.439624472 | 0.000729402 | | | HIST1H3C/HIST1H4B/HIST1H3H/H2AFZ/LMNB1/HIST1H3E/HIST1H2BC/HIST1H3J/H3F3A/HIST1H4L/HIST1H4E/HIST1H2AB/HIST1H2BO/HIST1H3B/HIST1H4H/HIST1H2BJ/HIST1H3G/HIST1H4C/HIST1H4D/HIST2H4A/HIST1H2AE/HIST1H2BN/HIST1H2AD/HIST1H3A/HIST1H2BH/HIST1H4F/HIST1H2BI/HIST1H2BL/HIST1H2BF/HIST1H2BG/HIST1H2BE/HIST1H4A/HIST1H2BB/HIST3H2BB/HIST2H3D/HIST1H2AJ/HIST1H2BM/HIST1H3D/HIST1H3F | | |  |
| R-HSA-1500620 | | Meiosis | | -2.439624472 | 0.000729402 | | | HIST1H3C/HIST1H4B/HIST1H3H/H2AFZ/LMNB1/HIST1H3E/HIST1H2BC/HIST1H3J/H3F3A/HIST1H4L/HIST1H4E/HIST1H2AB/HIST1H2BO/HIST1H3B/HIST1H4H/HIST1H2BJ/HIST1H3G/HIST1H4C/HIST1H4D/HIST2H4A/HIST1H2AE/HIST1H2BN/HIST1H2AD/HIST1H3A/HIST1H2BH/HIST1H4F/HIST1H2BI/HIST1H2BL/HIST1H2BF/HIST1H2BG/HIST1H2BE/HIST1H4A/HIST1H2BB/HIST3H2BB/HIST2H3D/HIST1H2AJ/HIST1H2BM/HIST1H3D/HIST1H3F | | |  |
| R-HSA-201681 | | TCF dependent signaling in response to WNT | | -2.726582739 | 0.000729402 | | | FZD5/HIST1H3C/HIST1H4B/HIST1H3H/H2AFZ/HIST1H3E/HIST1H2BC/HIST1H3J/H3F3A/HIST1H4L/HIST1H4E/HIST1H2AB/HIST1H2BO/TLE1/HIST1H3B/HIST1H4H/HIST1H2BJ/HIST1H3G/HIST1H4C/HIST1H4D/HIST2H4A/HIST1H2AE/HIST1H2BN/HIST1H2AD/HIST1H3A/HIST1H2BH/HIST1H4F/HIST1H2BI/KREMEN2/HIST1H2BL/HIST1H2BF/HIST1H2BG/HIST1H2BE/HIST1H4A/HIST1H2BB/HIST3H2BB/HIST2H3D/HIST1H2AJ/HIST1H2BM/HIST1H3D/HIST1H3F | | |  |
| R-HSA-69278 | | Cell Cycle. Mitotic | | -2.633541962 | 0.000729402 | | | RAD21/PPP1CB/CENPK/WAPL/KNL1/ANAPC15/HIST1H4J/CDK6/HIST2H3C/NCAPH/PRKAR2B/PPP2CB/HAUS2/PTTG1/AHCTF1/LIN54/HIST2H2AC/ITGB3BP/CCNE2/SMC2/CLASP2/HIST1H3I/NUP205/CDC25C/CDC20/CDCA8/KIF18A/CDCA5/HIST2H2BE/MASTL/PRIM1/SGO2/CENPO/HIST1H2BD/TMPO/HIST1H3C/BUB1/HIST1H4B/GTSE1/TPX2/TOP2A/CDC7/MAD2L1/HIST1H3H/NCAPG2/KIF2C/H2AFZ/LMNB1/HIST1H3E/HIST1H2BC/BORA/HIST1H3J/NEK2/CCNB2/UBE2C/KIF23/CDKN2C/H3F3A/NUP35/HIST1H4L/NDC80/CCNB1/CENPU/DBF4/HIST1H4E/CDK1/CENPH/CENPN/HIST1H2AB/BUB1B/HIST1H2BO/FBXO5/CENPP/WEE1/HIST1H3B/HIST1H4H/CCNA2/ORC1/HIST1H2BJ/HIST1H3G/HIST1H4C/BIRC5/SKP2/HIST1H4D/HIST2H4A/PKMYT1/CENPF/HAUS6/HIST1H2AE/KIF20A/SPC25/HIST1H2BN/HIST1H2AD/RFC3/SPDL1/HIST1H3A/HIST1H2BH/POLE4/CENPE/HIST1H4F/HIST1H2BI/HIST1H2BL/CENPA/HIST1H2BF/HIST1H2BG/HIST1H2BE/NUF2/HIST1H4A/HIST1H2BB/HIST3H2BB/HIST2H3D/HIST1H2AJ/HIST1H2BM/HIST1H3D/HIST1H3F | | |  |
| R-HSA-68875 | | Mitotic Prophase | | -2.844616468 | 0.000729402 | | | SMC2/HIST1H3I/NUP205/HIST2H2BE/MASTL/HIST1H2BD/TMPO/HIST1H3C/HIST1H4B/HIST1H3H/NCAPG2/H2AFZ/LMNB1/HIST1H3E/HIST1H2BC/HIST1H3J/CCNB2/H3F3A/NUP35/HIST1H4L/CCNB1/HIST1H4E/CDK1/HIST1H2AB/HIST1H2BO/HIST1H3B/HIST1H4H/HIST1H2BJ/HIST1H3G/HIST1H4C/HIST1H4D/HIST2H4A/HIST1H2AE/HIST1H2BN/HIST1H2AD/HIST1H3A/HIST1H2BH/HIST1H4F/HIST1H2BI/HIST1H2BL/HIST1H2BF/HIST1H2BG/HIST1H2BE/HIST1H4A/HIST1H2BB/HIST3H2BB/HIST2H3D/HIST1H2AJ/HIST1H2BM/HIST1H3D/HIST1H3F | | |  |
| R-HSA-1640170 | | Cell Cycle | | -2.548862302 | 0.000729402 | | | EXO1/RAD21/MND1/PPP1CB/CENPK/WAPL/KNL1/ANAPC15/HIST1H4J/CDK6/HIST2H3C/NCAPH/PRKAR2B/PPP2CB/HAUS2/PTTG1/AHCTF1/LIN54/BLM/HIST2H2AC/ITGB3BP/CCNE2/SMC2/CLASP2/HIST1H3I/NUP205/CDC25C/CDC20/CDCA8/KIF18A/CDCA5/HIST2H2BE/MASTL/PRIM1/SGO2/CENPO/HIST1H2BD/TMPO/HJURP/HIST1H3C/BUB1/HIST1H4B/GTSE1/TPX2/MIS18A/TOP2A/CDC7/MAD2L1/HIST1H3H/NCAPG2/KIF2C/H2AFZ/LMNB1/HIST1H3E/CENPW/HIST1H2BC/BORA/HIST1H3J/NPM1/RMI1/NEK2/CCNB2/UBE2C/KIF23/CDKN2C/H3F3A/NUP35/HIST1H4L/BARD1/NDC80/CCNB1/CENPU/DBF4/HIST1H4E/CDK1/CENPH/CENPN/HIST1H2AB/BUB1B/HIST1H2BO/FBXO5/CENPP/WEE1/HIST1H3B/HIST1H4H/CCNA2/ORC1/HIST1H2BJ/HIST1H3G/HIST1H4C/BIRC5/SKP2/HIST1H4D/HIST2H4A/PKMYT1/CENPF/HAUS6/HIST1H2AE/KIF20A/SPC25/HIST1H2BN/RAD1/HIST1H2AD/RFC3/SPDL1/HIST1H3A/HIST1H2BH/POLE4/CENPE/HIST1H4F/HIST1H2BI/HIST1H2BL/CENPA/HIST1H2BF/HIST1H2BG/HIST1H2BE/NUF2/HIST1H4A/HIST1H2BB/HIST3H2BB/HIST2H3D/HIST1H2AJ/HIST1H2BM/HIST1H3D/HIST1H3F | | |  |
| R-HSA-195721 | | Signaling by WNT | | -2.556094208 | 0.000729402 | | | FZD5/HIST1H3C/HIST1H4B/HIST1H3H/H2AFZ/HIST1H3E/HIST1H2BC/HIST1H3J/H3F3A/HIST1H4L/HIST1H4E/HIST1H2AB/HIST1H2BO/TLE1/HIST1H3B/HIST1H4H/HIST1H2BJ/HIST1H3G/HIST1H4C/HIST1H4D/HIST2H4A/HIST1H2AE/HIST1H2BN/WNT2B/HIST1H2AD/HIST1H3A/HIST1H2BH/HIST1H4F/HIST1H2BI/KREMEN2/HIST1H2BL/HIST1H2BF/HIST1H2BG/HIST1H2BE/HIST1H4A/HIST1H2BB/HIST3H2BB/HIST2H3D/HIST1H2AJ/HIST1H2BM/HIST1H3D/ROR2/HIST1H3F | | |  |
| R-HSA-73886 | | Chromosome Maintenance | | -2.507349486 | 0.000729402 | | | PRIM1/CENPO/HIST1H2BD/HJURP/HIST1H4B/MIS18A/H2AFZ/CENPW/HIST1H2BC/NPM1/HIST1H4L/CENPU/HIST1H4E/CENPH/CENPN/HIST1H2AB/HIST1H2BO/CENPP/HIST1H4H/HIST1H2BJ/HIST1H4C/HIST1H4D/HIST2H4A/HIST1H2AE/HIST1H2BN/HIST1H2AD/RFC3/HIST1H2BH/POLE4/HIST1H4F/HIST1H2BI/HIST1H2BL/CENPA/HIST1H2BF/HIST1H2BG/HIST1H2BE/HIST1H4A/HIST1H2BB/HIST3H2BB/HIST1H2AJ/HIST1H2BM | | |  |
| R-HSA-211000 | | Gene Silencing by RNA | | -2.540792618 | 0.000729402 | | | HIST1H3C/HIST1H4B/HIST1H3H/H2AFZ/HIST1H3E/HIST1H2BC/HIST1H3J/H3F3A/NUP35/HIST1H4L/HIST1H4E/HIST1H2AB/HIST1H2BO/HIST1H3B/HIST1H4H/HIST1H2BJ/HIST1H3G/HIST1H4C/HIST1H4D/HIST2H4A/HIST1H2AE/HIST1H2BN/HIST1H2AD/HIST1H3A/HIST1H2BH/HIST1H4F/HIST1H2BI/HIST1H2BL/HIST1H2BF/HIST1H2BG/HIST1H2BE/HIST1H4A/HIST1H2BB/HIST3H2BB/HIST2H3D/HIST1H2AJ/HIST1H2BM/HIST1H3D/HIST1H3F | | |  |
| R-HSA-8878171 | | Transcriptional regulation by RUNX1 | | -2.719554625 | 0.000729402 | | | HIST1H3C/HIST1H4B/HIST1H3H/H2AFZ/HIST1H3E/HIST1H2BC/HIST1H3J/H3F3A/HIST1H4L/HIST1H4E/HIST1H2AB/HIST1H2BO/HIST1H3B/HIST1H4H/HIST1H2BJ/HIST1H3G/HIST1H4C/HIST1H4D/HIST2H4A/HIST1H2AE/HIST1H2BN/HIST1H2AD/HIST1H3A/HIST1H2BH/HIST1H4F/HIST1H2BI/HIST1H2BL/HIST1H2BF/HIST1H2BG/HIST1H2BE/HIST1H4A/HIST1H2BB/HIST3H2BB/HIST2H3D/HIST1H2AJ/HIST1H2BM/HIST1H3D/HIST1H3F | | |  |
| R-HSA-212165 | | Epigenetic regulation of gene expression | | -2.537060454 | 0.000729402 | | | HIST1H2BD/HIST1H3C/HIST1H4B/HIST1H3H/H2AFZ/HIST1H3E/HIST1H2BC/HIST1H3J/H3F3A/HIST1H4L/TDG/HIST1H4E/HIST1H2AB/HIST1H2BO/HIST1H3B/HIST1H4H/HIST1H2BJ/HIST1H3G/HIST1H4C/HIST1H4D/HIST2H4A/HIST1H2AE/HIST1H2BN/HIST1H2AD/HIST1H3A/HIST1H2BH/HIST1H4F/HIST1H2BI/HIST1H2BL/DNMT3B/HIST1H2BF/HIST1H2BG/HIST1H2BE/HIST1H4A/HIST1H2BB/HIST3H2BB/HIST2H3D/HIST1H2AJ/HIST1H2BM/HIST1H3D/HIST1H3F | | |  |
| R-HSA-73772 | | RNA Polymerase I Promoter Escape | | -2.712608936 | 0.000729402 | | | HIST1H3C/HIST1H4B/HIST1H3H/H2AFZ/HIST1H3E/HIST1H2BC/HIST1H3J/H3F3A/HIST1H4L/HIST1H4E/HIST1H2AB/HIST1H2BO/HIST1H3B/HIST1H4H/HIST1H2BJ/HIST1H3G/HIST1H4C/HIST1H4D/HIST2H4A/HIST1H2AE/HIST1H2BN/HIST1H2AD/HIST1H3A/HIST1H2BH/HIST1H4F/HIST1H2BI/HIST1H2BL/HIST1H2BF/HIST1H2BG/HIST1H2BE/HIST1H4A/HIST1H2BB/HIST3H2BB/HIST2H3D/HIST1H2AJ/HIST1H2BM/HIST1H3D/HIST1H3F | | |  |
| R-HSA-201722 | | Formation of the beta-catenin:TCF transactivating complex | | -2.813765928 | 0.000729402 | | | HIST1H3C/HIST1H4B/HIST1H3H/H2AFZ/HIST1H3E/HIST1H2BC/HIST1H3J/H3F3A/HIST1H4L/HIST1H4E/HIST1H2AB/HIST1H2BO/TLE1/HIST1H3B/HIST1H4H/HIST1H2BJ/HIST1H3G/HIST1H4C/HIST1H4D/HIST2H4A/HIST1H2AE/HIST1H2BN/HIST1H2AD/HIST1H3A/HIST1H2BH/HIST1H4F/HIST1H2BI/HIST1H2BL/HIST1H2BF/HIST1H2BG/HIST1H2BE/HIST1H4A/HIST1H2BB/HIST3H2BB/HIST2H3D/HIST1H2AJ/HIST1H2BM/HIST1H3D/HIST1H3F | | |  |
| R-HSA-212300 | | PRC2 methylates histones and DNA | | -2.818164976 | 0.000729402 | | | HIST1H3C/HIST1H4B/HIST1H3H/H2AFZ/HIST1H3E/HIST1H2BC/HIST1H3J/H3F3A/HIST1H4L/HIST1H4E/HIST1H2AB/HIST1H2BO/HIST1H3B/HIST1H4H/HIST1H2BJ/HIST1H3G/HIST1H4C/HIST1H4D/HIST2H4A/HIST1H2AE/HIST1H2BN/HIST1H2AD/HIST1H3A/HIST1H2BH/HIST1H4F/HIST1H2BI/HIST1H2BL/DNMT3B/HIST1H2BF/HIST1H2BG/HIST1H2BE/HIST1H4A/HIST1H2BB/HIST3H2BB/HIST2H3D/HIST1H2AJ/HIST1H2BM/HIST1H3D/HIST1H3F | | |  |
| R-HSA-5334118 | | DNA methylation | | -2.816366556 | 0.000729402 | | | HIST1H3C/HIST1H4B/HIST1H3H/H2AFZ/HIST1H3E/HIST1H2BC/HIST1H3J/H3F3A/HIST1H4L/HIST1H4E/HIST1H2AB/HIST1H2BO/HIST1H3B/HIST1H4H/HIST1H2BJ/HIST1H3G/HIST1H4C/HIST1H4D/HIST2H4A/HIST1H2AE/HIST1H2BN/HIST1H2AD/HIST1H3A/HIST1H2BH/HIST1H4F/HIST1H2BI/HIST1H2BL/DNMT3B/HIST1H2BF/HIST1H2BG/HIST1H2BE/HIST1H4A/HIST1H2BB/HIST3H2BB/HIST2H3D/HIST1H2AJ/HIST1H2BM/HIST1H3D/HIST1H3F | | |  |
| R-HSA-3214847 | | HATs acetylate histones | | -2.740784945 | 0.000729402 | | | HIST1H3C/HIST1H4B/HIST1H3H/HIST1H3E/HIST1H2BC/HIST1H3J/HIST1H2AL/HIST1H4L/HIST1H4E/HIST1H2AM/HIST1H2AB/HIST1H2BO/HIST1H3B/HIST1H4H/HIST1H2BJ/HIST1H3G/HIST1H4C/HIST1H4D/HIST2H4A/HIST1H2AE/HIST1H2BN/HIST1H2AD/HIST2H2BF/TAF5L/HIST1H2AH/HIST1H3A/HIST1H2AI/HIST1H2BH/HIST1H4F/HIST1H2BI/HIST1H2BL/HIST1H2BF/HIST1H2BG/HIST1H2BE/HIST1H4A/HIST1H2BB/HIST3H2BB/HIST2H3D/HIST1H2AJ/HIST1H2AK/HIST1H2BM/HIST1H3D/HIST1H3F | | |  |
| R-HSA-2299718 | | Condensation of Prophase Chromosomes | | -2.824198978 | 0.000729402 | | | HIST1H3C/HIST1H4B/HIST1H3H/NCAPG2/H2AFZ/HIST1H3E/HIST1H2BC/HIST1H3J/H3F3A/HIST1H4L/CCNB1/HIST1H4E/CDK1/HIST1H2AB/HIST1H2BO/HIST1H3B/HIST1H4H/HIST1H2BJ/HIST1H3G/HIST1H4C/HIST1H4D/HIST2H4A/HIST1H2AE/HIST1H2BN/HIST1H2AD/HIST1H3A/HIST1H2BH/HIST1H4F/HIST1H2BI/HIST1H2BL/HIST1H2BF/HIST1H2BG/HIST1H2BE/HIST1H4A/HIST1H2BB/HIST3H2BB/HIST2H3D/HIST1H2AJ/HIST1H2BM/HIST1H3D/HIST1H3F | | |  |
| R-HSA-427413 | | NoRC negatively regulates rRNA expression | | -2.710249579 | 0.000729402 | | | HIST1H2BD/HIST1H3C/HIST1H4B/HIST1H3H/H2AFZ/HIST1H3E/HIST1H2BC/HIST1H3J/H3F3A/HIST1H4L/HIST1H4E/HIST1H2AB/HIST1H2BO/HIST1H3B/HIST1H4H/HIST1H2BJ/HIST1H3G/HIST1H4C/HIST1H4D/HIST2H4A/HIST1H2AE/HIST1H2BN/HIST1H2AD/HIST1H3A/HIST1H2BH/HIST1H4F/HIST1H2BI/HIST1H2BL/DNMT3B/HIST1H2BF/HIST1H2BG/HIST1H2BE/HIST1H4A/HIST1H2BB/HIST3H2BB/HIST2H3D/HIST1H2AJ/HIST1H2BM/HIST1H3D/HIST1H3F | | |  |
| R-HSA-5250941 | | Negative epigenetic regulation of rRNA expression | | -2.710249579 | 0.000729402 | | | HIST1H2BD/HIST1H3C/HIST1H4B/HIST1H3H/H2AFZ/HIST1H3E/HIST1H2BC/HIST1H3J/H3F3A/HIST1H4L/HIST1H4E/HIST1H2AB/HIST1H2BO/HIST1H3B/HIST1H4H/HIST1H2BJ/HIST1H3G/HIST1H4C/HIST1H4D/HIST2H4A/HIST1H2AE/HIST1H2BN/HIST1H2AD/HIST1H3A/HIST1H2BH/HIST1H4F/HIST1H2BI/HIST1H2BL/DNMT3B/HIST1H2BF/HIST1H2BG/HIST1H2BE/HIST1H4A/HIST1H2BB/HIST3H2BB/HIST2H3D/HIST1H2AJ/HIST1H2BM/HIST1H3D/HIST1H3F | | |  |
| R-HSA-977225 | | Amyloid fiber formation | | -2.745403375 | 0.000729402 | | | HIST1H3C/HIST1H4B/HIST1H3H/H2AFZ/HIST1H3E/HIST1H2BC/HIST1H3J/H3F3A/HIST1H4L/HIST1H4E/HIST1H2AB/HIST1H2BO/HIST1H3B/HIST1H4H/HIST1H2BJ/HIST1H3G/HIST1H4C/HIST1H4D/HIST2H4A/HIST1H2AE/HIST1H2BN/HIST1H2AD/HIST1H3A/HIST1H2BH/HIST1H4F/HIST1H2BI/HIST1H2BL/HIST1H2BF/HIST1H2BG/SNCAIP/HIST1H2BE/HIST1H4A/HIST1H2BB/HIST3H2BB/HIST2H3D/HIST1H2AJ/HIST1H2BM/HIST1H3D/LTF/HIST1H3F | | |  |
| R-HSA-912446 | | Meiotic recombination | | -2.497372896 | 0.000729402 | | | HIST1H3C/HIST1H4B/HIST1H3H/H2AFZ/HIST1H3E/HIST1H2BC/HIST1H3J/H3F3A/HIST1H4L/HIST1H4E/HIST1H2AB/HIST1H2BO/HIST1H3B/HIST1H4H/HIST1H2BJ/HIST1H3G/HIST1H4C/HIST1H4D/HIST2H4A/HIST1H2AE/HIST1H2BN/HIST1H2AD/HIST1H3A/HIST1H2BH/HIST1H4F/HIST1H2BI/HIST1H2BL/HIST1H2BF/HIST1H2BG/HIST1H2BE/HIST1H4A/HIST1H2BB/HIST3H2BB/HIST2H3D/HIST1H2AJ/HIST1H2BM/HIST1H3D/HIST1H3F | | |  |
| R-HSA-5250913 | | Positive epigenetic regulation of rRNA expression | | -2.551012213 | 0.000729402 | | | HIST1H3C/HIST1H4B/HIST1H3H/H2AFZ/HIST1H3E/HIST1H2BC/HIST1H3J/H3F3A/HIST1H4L/HIST1H4E/HIST1H2AB/HIST1H2BO/HIST1H3B/HIST1H4H/HIST1H2BJ/HIST1H3G/HIST1H4C/HIST1H4D/HIST2H4A/HIST1H2AE/HIST1H2BN/HIST1H2AD/HIST1H3A/HIST1H2BH/HIST1H4F/HIST1H2BI/HIST1H2BL/HIST1H2BF/HIST1H2BG/HIST1H2BE/HIST1H4A/HIST1H2BB/HIST3H2BB/HIST2H3D/HIST1H2AJ/HIST1H2BM/HIST1H3D/HIST1H3F | | |  |
| R-HSA-5617472 | | Activation of anterior HOX genes in hindbrain development during early embryogenesis | | -2.561227035 | 0.000729402 | | | HIST1H3C/HIST1H4B/HIST1H3H/H2AFZ/HIST1H3E/HIST1H2BC/HIST1H3J/H3F3A/HIST1H4L/HIST1H4E/HIST1H2AB/HIST1H2BO/HIST1H3B/HIST1H4H/HIST1H2BJ/HIST1H3G/HIST1H4C/HIST1H4D/HIST2H4A/HIST1H2AE/HIST1H2BN/HIST1H2AD/HIST1H3A/HIST1H2BH/HIST1H4F/HIST1H2BI/HIST1H2BL/HIST1H2BF/HIST1H2BG/HIST1H2BE/HIST1H4A/HIST1H2BB/HIST3H2BB/HIST2H3D/HIST1H2AJ/HIST1H2BM/HIST1H3D/HIST1H3F | | |  |
| R-HSA-5619507 | | Activation of HOX genes during differentiation | | -2.561227035 | 0.000729402 | | | HIST1H3C/HIST1H4B/HIST1H3H/H2AFZ/HIST1H3E/HIST1H2BC/HIST1H3J/H3F3A/HIST1H4L/HIST1H4E/HIST1H2AB/HIST1H2BO/HIST1H3B/HIST1H4H/HIST1H2BJ/HIST1H3G/HIST1H4C/HIST1H4D/HIST2H4A/HIST1H2AE/HIST1H2BN/HIST1H2AD/HIST1H3A/HIST1H2BH/HIST1H4F/HIST1H2BI/HIST1H2BL/HIST1H2BF/HIST1H2BG/HIST1H2BE/HIST1H4A/HIST1H2BB/HIST3H2BB/HIST2H3D/HIST1H2AJ/HIST1H2BM/HIST1H3D/HIST1H3F | | |  |
| R-HSA-8939211 | | ESR-mediated signaling | | -2.682024319 | 0.000729402 | | | HIST1H3C/HIST1H4B/NRIP1/HIST1H3H/H2AFZ/HIST1H3E/HIST1H2BC/HIST1H3J/H3F3A/HIST1H4L/KPNA2/HIST1H4E/HIST1H2AB/GTF2F2/HIST1H2BO/HIST1H3B/HIST1H4H/HIST1H2BJ/HIST1H3G/HIST1H4C/HIST1H4D/HIST2H4A/HIST1H2AE/HIST1H2BN/HIST1H2AD/HIST1H3A/HIST1H2BH/HIST1H4F/HIST1H2BI/HIST1H2BL/HIST1H2BF/HIST1H2BG/HIST1H2BE/FKBP5/HIST1H4A/HIST1H2BB/HIST3H2BB/HIST2H3D/HIST1H2AJ/HIST1H2BM/HIST1H3D/ERBB4/EPGN/HIST1H3F | | |  |
| R-HSA-5250924 | | B-WICH complex positively regulates rRNA expression | | -2.602282461 | 0.000729402 | | | HIST1H3C/HIST1H4B/HIST1H3H/H2AFZ/HIST1H3E/HIST1H2BC/HIST1H3J/H3F3A/HIST1H4L/HIST1H4E/HIST1H2AB/HIST1H2BO/HIST1H3B/HIST1H4H/HIST1H2BJ/HIST1H3G/HIST1H4C/HIST1H4D/HIST2H4A/HIST1H2AE/HIST1H2BN/HIST1H2AD/HIST1H3A/HIST1H2BH/HIST1H4F/HIST1H2BI/HIST1H2BL/HIST1H2BF/HIST1H2BG/HIST1H2BE/HIST1H4A/HIST1H2BB/HIST3H2BB/HIST2H3D/HIST1H2AJ/HIST1H2BM/HIST1H3D/HIST1H3F | | |  |
| R-HSA-5625740 | | RHO GTPases activate PKNs | | -2.716344286 | 0.000729402 | | | RHOA/CDC25C/HIST2H2BE/HIST1H2BD/HIST1H3C/HIST1H4B/HIST1H3H/H2AFZ/HIST1H3E/HIST1H2BC/HIST1H3J/H3F3A/HIST1H4L/HIST1H4E/HIST1H2AB/HIST1H2BO/PAK1/HIST1H3B/HIST1H4H/HIST1H2BJ/HIST1H3G/HIST1H4C/HIST1H4D/HIST2H4A/HIST1H2AE/HIST1H2BN/HIST1H2AD/HIST1H3A/HIST1H2BH/HIST1H4F/HIST1H2BI/HIST1H2BL/HIST1H2BF/HIST1H2BG/HIST1H2BE/HIST1H4A/HIST1H2BB/HIST3H2BB/HIST2H3D/HIST1H2AJ/HIST1H2BM/HIST1H3D/HIST1H3F | | |  |
| R-HSA-606279 | | Deposition of new CENPA-containing nucleosomes at the centromere | | -2.598279844 | 0.000729402 | | | HIST1H2BD/HJURP/HIST1H4B/MIS18A/H2AFZ/CENPW/HIST1H2BC/NPM1/HIST1H4L/CENPU/HIST1H4E/CENPH/CENPN/HIST1H2AB/HIST1H2BO/CENPP/HIST1H4H/HIST1H2BJ/HIST1H4C/HIST1H4D/HIST2H4A/HIST1H2AE/HIST1H2BN/HIST1H2AD/HIST1H2BH/HIST1H4F/HIST1H2BI/HIST1H2BL/CENPA/HIST1H2BF/HIST1H2BG/HIST1H2BE/HIST1H4A/HIST1H2BB/HIST3H2BB/HIST1H2AJ/HIST1H2BM | | |  |
| R-HSA-774815 | | Nucleosome assembly | | -2.598279844 | 0.000729402 | | | HIST1H2BD/HJURP/HIST1H4B/MIS18A/H2AFZ/CENPW/HIST1H2BC/NPM1/HIST1H4L/CENPU/HIST1H4E/CENPH/CENPN/HIST1H2AB/HIST1H2BO/CENPP/HIST1H4H/HIST1H2BJ/HIST1H4C/HIST1H4D/HIST2H4A/HIST1H2AE/HIST1H2BN/HIST1H2AD/HIST1H2BH/HIST1H4F/HIST1H2BI/HIST1H2BL/CENPA/HIST1H2BF/HIST1H2BG/HIST1H2BE/HIST1H4A/HIST1H2BB/HIST3H2BB/HIST1H2AJ/HIST1H2BM | | |  |
| R-HSA-2559583 | | Cellular Senescence | | -2.523044785 | 0.000729402 | | | HIST1H3C/HIST1H4B/HIST1H3H/H2AFZ/LMNB1/HIST1H3E/HIST1H2BC/HIST1H3J/UBE2C/CDKN2C/H3F3A/HIST1H4L/HIST1H4E/HIST1H2AB/HIST1H2BO/HIST1H3B/HIST1H4H/CCNA2/HIST1H2BJ/HIST1H3G/HIST1H4C/HIST1H4D/HIST2H4A/HIST1H2AE/HIST1H2BN/HIST1H2AD/HIST1H3A/HIST1H2BH/HIST1H4F/HIST1H1B/HIST1H2BI/HIST1H2BL/HIST1H2BF/HIST1H2BG/HIST1H2BE/HIST1H1D/HIST1H4A/HIST1H2BB/HIST3H2BB/HIST2H3D/HIST1H2AJ/HIST1H2BM/HIST1H3D/HIST1H3F | | |  |
| R-HSA-3247509 | | Chromatin modifying enzymes | | -2.591415445 | 0.000729402 | | | HIST2H2AC/YEATS4/HIST1H3I/HIST2H2AB/HIST2H2BE/KDM4D/SAP30/HIST1H2BD/HIST1H3C/HIST1H4B/HIST1H3H/H2AFZ/HIST1H3E/RPS2/HIST1H2BC/HIST1H3J/HIST1H2AL/HIST1H4L/HIST1H4E/HIST1H2AM/HIST1H2AB/HIST1H2BO/ARID5B/HIST1H3B/HIST1H4H/HIST1H2BJ/HIST1H3G/HIST1H4C/HIST1H4D/HIST2H4A/HIST1H2AE/HIST1H2BN/HIST1H2AD/HIST2H2BF/TAF5L/HIST1H2AH/HIST1H3A/HIST1H2AI/HIST1H2BH/HIST1H4F/HIST1H2BI/HIST1H2BL/HIST1H2BF/HIST1H2BG/HIST1H2BE/HIST1H4A/HIST1H2BB/HIST3H2BB/HIST2H3D/HIST1H2AJ/HIST1H2AK/HIST1H2BM/HIST1H3D/HIST1H3F | | |  |
| R-HSA-4839726 | | Chromatin organization | | -2.591415445 | 0.000729402 | | | HIST2H2AC/YEATS4/HIST1H3I/HIST2H2AB/HIST2H2BE/KDM4D/SAP30/HIST1H2BD/HIST1H3C/HIST1H4B/HIST1H3H/H2AFZ/HIST1H3E/RPS2/HIST1H2BC/HIST1H3J/HIST1H2AL/HIST1H4L/HIST1H4E/HIST1H2AM/HIST1H2AB/HIST1H2BO/ARID5B/HIST1H3B/HIST1H4H/HIST1H2BJ/HIST1H3G/HIST1H4C/HIST1H4D/HIST2H4A/HIST1H2AE/HIST1H2BN/HIST1H2AD/HIST2H2BF/TAF5L/HIST1H2AH/HIST1H3A/HIST1H2AI/HIST1H2BH/HIST1H4F/HIST1H2BI/HIST1H2BL/HIST1H2BF/HIST1H2BG/HIST1H2BE/HIST1H4A/HIST1H2BB/HIST3H2BB/HIST2H3D/HIST1H2AJ/HIST1H2AK/HIST1H2BM/HIST1H3D/HIST1H3F | | |  |
| R-HSA-427359 | | SIRT1 negatively regulates rRNA expression | | -2.781436462 | 0.000729402 | | | HIST1H3C/HIST1H4B/HIST1H3H/H2AFZ/HIST1H3E/HIST1H2BC/HIST1H3J/H3F3A/HIST1H4L/HIST1H4E/HIST1H2AB/HIST1H2BO/HIST1H3B/HIST1H4H/HIST1H2BJ/HIST1H3G/HIST1H4C/HIST1H4D/HIST2H4A/HIST1H2AE/HIST1H2BN/HIST1H2AD/HIST1H3A/HIST1H2BH/HIST1H4F/HIST1H2BI/HIST1H2BL/HIST1H2BF/HIST1H2BG/HIST1H2BE/HIST1H4A/HIST1H2BB/HIST3H2BB/HIST2H3D/HIST1H2AJ/HIST1H2BM/HIST1H3D/HIST1H3F | | |  |
| R-HSA-427389 | | ERCC6 (CSB) and EHMT2 (G9a) positively regulate rRNA expression | | -2.78764676 | 0.000729402 | | | HIST1H3C/HIST1H4B/HIST1H3H/H2AFZ/HIST1H3E/HIST1H2BC/HIST1H3J/H3F3A/HIST1H4L/HIST1H4E/HIST1H2AB/HIST1H2BO/HIST1H3B/HIST1H4H/HIST1H2BJ/HIST1H3G/HIST1H4C/HIST1H4D/HIST2H4A/HIST1H2AE/HIST1H2BN/HIST1H2AD/HIST1H3A/HIST1H2BH/HIST1H4F/HIST1H2BI/HIST1H2BL/HIST1H2BF/HIST1H2BG/HIST1H2BE/HIST1H4A/HIST1H2BB/HIST3H2BB/HIST2H3D/HIST1H2AJ/HIST1H2BM/HIST1H3D/HIST1H3F | | |  |
| R-HSA-8936459 | | RUNX1 regulates genes involved in megakaryocyte differentiation and platelet function | | -2.784179702 | 0.000729402 | | | HIST1H3C/HIST1H4B/HIST1H3H/H2AFZ/HIST1H3E/HIST1H2BC/HIST1H3J/H3F3A/HIST1H4L/HIST1H4E/HIST1H2AB/HIST1H2BO/HIST1H3B/HIST1H4H/HIST1H2BJ/HIST1H3G/HIST1H4C/HIST1H4D/HIST2H4A/HIST1H2AE/HIST1H2BN/HIST1H2AD/HIST1H3A/HIST1H2BH/HIST1H4F/HIST1H2BI/HIST1H2BL/HIST1H2BF/HIST1H2BG/HIST1H2BE/HIST1H4A/HIST1H2BB/HIST3H2BB/HIST2H3D/HIST1H2AJ/HIST1H2BM/HIST1H3D/HIST1H3F | | |  |
| R-HSA-9006931 | | Signaling by Nuclear Receptors | | -2.554672954 | 0.000729402 | | | HIST1H3C/HIST1H4B/NRIP1/HIST1H3H/H2AFZ/HIST1H3E/HIST1H2BC/HIST1H3J/H3F3A/HIST1H4L/KPNA2/HIST1H4E/HIST1H2AB/GTF2F2/HIST1H2BO/ALDH1A3/HIST1H3B/HIST1H4H/HIST1H2BJ/HIST1H3G/HIST1H4C/HIST1H4D/HIST2H4A/HIST1H2AE/HIST1H2BN/HIST1H2AD/HIST1H3A/HIST1H2BH/HIST1H4F/RDH10/HIST1H2BI/HIST1H2BL/HIST1H2BF/HIST1H2BG/HIST1H2BE/PDK1/FKBP5/HIST1H4A/HIST1H2BB/HIST3H2BB/HIST2H3D/HIST1H2AJ/HIST1H2BM/HIST1H3D/ERBB4/EPGN/HIST1H3F | | |  |
| R-HSA-2559580 | | Oxidative Stress Induced Senescence | | -2.754620722 | 0.000729402 | | | HIST1H3C/HIST1H4B/HIST1H3H/H2AFZ/HIST1H3E/HIST1H2BC/HIST1H3J/CDKN2C/H3F3A/HIST1H4L/HIST1H4E/HIST1H2AB/HIST1H2BO/HIST1H3B/HIST1H4H/HIST1H2BJ/HIST1H3G/HIST1H4C/HIST1H4D/HIST2H4A/HIST1H2AE/HIST1H2BN/HIST1H2AD/HIST1H3A/HIST1H2BH/HIST1H4F/HIST1H2BI/HIST1H2BL/HIST1H2BF/HIST1H2BG/HIST1H2BE/HIST1H4A/HIST1H2BB/HIST3H2BB/HIST2H3D/HIST1H2AJ/HIST1H2BM/HIST1H3D/HIST1H3F | | |  |
| R-HSA-73864 | | RNA Polymerase I Transcription | | -2.657699328 | 0.000729402 | | | HIST1H3C/HIST1H4B/HIST1H3H/H2AFZ/HIST1H3E/HIST1H2BC/HIST1H3J/H3F3A/HIST1H4L/HIST1H4E/HIST1H2AB/HIST1H2BO/HIST1H3B/HIST1H4H/HIST1H2BJ/HIST1H3G/HIST1H4C/HIST1H4D/HIST2H4A/HIST1H2AE/HIST1H2BN/HIST1H2AD/HIST1H3A/HIST1H2BH/HIST1H4F/HIST1H2BI/HIST1H2BL/HIST1H2BF/HIST1H2BG/HIST1H2BE/HIST1H4A/HIST1H2BB/HIST3H2BB/HIST2H3D/HIST1H2AJ/HIST1H2BM/HIST1H3D/HIST1H3F | | |  |
| R-HSA-73854 | | RNA Polymerase I Promoter Clearance | | -2.657699328 | 0.000729402 | | | HIST1H3C/HIST1H4B/HIST1H3H/H2AFZ/HIST1H3E/HIST1H2BC/HIST1H3J/H3F3A/HIST1H4L/HIST1H4E/HIST1H2AB/HIST1H2BO/HIST1H3B/HIST1H4H/HIST1H2BJ/HIST1H3G/HIST1H4C/HIST1H4D/HIST2H4A/HIST1H2AE/HIST1H2BN/HIST1H2AD/HIST1H3A/HIST1H2BH/HIST1H4F/HIST1H2BI/HIST1H2BL/HIST1H2BF/HIST1H2BG/HIST1H2BE/HIST1H4A/HIST1H2BB/HIST3H2BB/HIST2H3D/HIST1H2AJ/HIST1H2BM/HIST1H3D/HIST1H3F | | |  |
| R-HSA-5578749 | | Transcriptional regulation by small RNAs | | -2.750704325 | 0.000729402 | | | HIST1H3C/HIST1H4B/HIST1H3H/H2AFZ/HIST1H3E/HIST1H2BC/HIST1H3J/H3F3A/NUP35/HIST1H4L/HIST1H4E/HIST1H2AB/HIST1H2BO/HIST1H3B/HIST1H4H/HIST1H2BJ/HIST1H3G/HIST1H4C/HIST1H4D/HIST2H4A/HIST1H2AE/HIST1H2BN/HIST1H2AD/HIST1H3A/HIST1H2BH/HIST1H4F/HIST1H2BI/HIST1H2BL/HIST1H2BF/HIST1H2BG/HIST1H2BE/HIST1H4A/HIST1H2BB/HIST3H2BB/HIST2H3D/HIST1H2AJ/HIST1H2BM/HIST1H3D/HIST1H3F | | |  |
| R-HSA-1912408 | | Pre-NOTCH Transcription and Translation | | -2.837997512 | 0.000729402 | | | HIST1H3C/HIST1H4B/HIST1H3H/H2AFZ/HIST1H3E/HIST1H2BC/HIST1H3J/H3F3A/HIST1H4L/HIST1H4E/HIST1H2AB/HIST1H2BO/HIST1H3B/HIST1H4H/HIST1H2BJ/HIST1H3G/HIST1H4C/HIST1H4D/HIST2H4A/HIST1H2AE/HIST1H2BN/HIST1H2AD/HIST1H3A/HIST1H2BH/HIST1H4F/HIST1H2BI/HIST1H2BL/HIST1H2BF/HIST1H2BG/HIST1H2BE/HIST1H4A/HIST1H2BB/HIST3H2BB/HIST2H3D/HIST1H2AJ/HIST1H2BM/HIST1H3D/HIST1H3F | | |  |
| R-HSA-1912422 | | Pre-NOTCH Expression and Processing | | -2.837997512 | 0.000729402 | | | HIST1H3C/HIST1H4B/HIST1H3H/H2AFZ/HIST1H3E/HIST1H2BC/HIST1H3J/H3F3A/HIST1H4L/HIST1H4E/HIST1H2AB/HIST1H2BO/HIST1H3B/HIST1H4H/HIST1H2BJ/HIST1H3G/HIST1H4C/HIST1H4D/HIST2H4A/HIST1H2AE/HIST1H2BN/HIST1H2AD/HIST1H3A/HIST1H2BH/HIST1H4F/HIST1H2BI/HIST1H2BL/HIST1H2BF/HIST1H2BG/HIST1H2BE/HIST1H4A/HIST1H2BB/HIST3H2BB/HIST2H3D/HIST1H2AJ/HIST1H2BM/HIST1H3D/HIST1H3F | | |  |
| R-HSA-8939236 | | RUNX1 regulates transcription of genes involved in differentiation of HSCs | | -2.837997512 | 0.000729402 | | | HIST1H3C/HIST1H4B/HIST1H3H/H2AFZ/HIST1H3E/HIST1H2BC/HIST1H3J/H3F3A/HIST1H4L/HIST1H4E/HIST1H2AB/HIST1H2BO/HIST1H3B/HIST1H4H/HIST1H2BJ/HIST1H3G/HIST1H4C/HIST1H4D/HIST2H4A/HIST1H2AE/HIST1H2BN/HIST1H2AD/HIST1H3A/HIST1H2BH/HIST1H4F/HIST1H2BI/HIST1H2BL/HIST1H2BF/HIST1H2BG/HIST1H2BE/HIST1H4A/HIST1H2BB/HIST3H2BB/HIST2H3D/HIST1H2AJ/HIST1H2BM/HIST1H3D/HIST1H3F | | |  |
| R-HSA-5625886 | | Activated PKN1 stimulates transcription of AR (androgen receptor) regulated genes KLK2 and KLK3 | | -2.837997512 | 0.000729402 | | | HIST1H3C/HIST1H4B/HIST1H3H/H2AFZ/HIST1H3E/HIST1H2BC/HIST1H3J/H3F3A/HIST1H4L/HIST1H4E/HIST1H2AB/HIST1H2BO/HIST1H3B/HIST1H4H/HIST1H2BJ/HIST1H3G/HIST1H4C/HIST1H4D/HIST2H4A/HIST1H2AE/HIST1H2BN/HIST1H2AD/HIST1H3A/HIST1H2BH/HIST1H4F/HIST1H2BI/HIST1H2BL/HIST1H2BF/HIST1H2BG/HIST1H2BE/HIST1H4A/HIST1H2BB/HIST3H2BB/HIST2H3D/HIST1H2AJ/HIST1H2BM/HIST1H3D/HIST1H3F | | |  |
| R-HSA-73728 | | RNA Polymerase I Promoter Opening | | -2.837997512 | 0.000729402 | | | HIST1H3C/HIST1H4B/HIST1H3H/H2AFZ/HIST1H3E/HIST1H2BC/HIST1H3J/H3F3A/HIST1H4L/HIST1H4E/HIST1H2AB/HIST1H2BO/HIST1H3B/HIST1H4H/HIST1H2BJ/HIST1H3G/HIST1H4C/HIST1H4D/HIST2H4A/HIST1H2AE/HIST1H2BN/HIST1H2AD/HIST1H3A/HIST1H2BH/HIST1H4F/HIST1H2BI/HIST1H2BL/HIST1H2BF/HIST1H2BG/HIST1H2BE/HIST1H4A/HIST1H2BB/HIST3H2BB/HIST2H3D/HIST1H2AJ/HIST1H2BM/HIST1H3D/HIST1H3F | | |  |
| R-HSA-69481 | | G2/M Checkpoints | | -2.520623661 | 0.000729402 | | | HIST1H4B/GTSE1/CDC7/HIST1H2BC/RMI1/CCNB2/HIST1H4L/BARD1/CCNB1/DBF4/HIST1H4E/CDK1/HIST1H2BO/WEE1/HIST1H4H/ORC1/HIST1H2BJ/HIST1H4C/HIST1H4D/HIST2H4A/PKMYT1/HIST1H2BN/RAD1/RFC3/HIST1H2BH/HIST1H4F/HIST1H2BI/HIST1H2BL/HIST1H2BF/HIST1H2BG/HIST1H2BE/HIST1H4A/HIST1H2BB/HIST3H2BB/HIST1H2BM | | |  |
| R-HSA-1266738 | | Developmental Biology | | -2.06541042 | 0.001144657 | | | HIST1H2BD/HES1/HIST1H3C/RPS29/HIST1H4B/CD24/RPL22L1/HIST1H3H/H2AFZ/HIST1H3E/RPS2/HIST1H2BC/HIST1H3J/H3F3A/HIST1H4L/KIF4A/HIST1H4E/HIST1H2AB/HIST1H2BO/PAK1/HIST1H3B/VLDLR/KRT10/HIST1H4H/PKP2/HIST1H2BJ/HIST1H3G/HIST1H4C/HIST1H4D/HIST2H4A/HIST1H2AE/HIST1H2BN/HIST1H2AD/HIST1H3A/HIST1H2BH/SEMA7A/SEMA5A/HIST1H4F/HIST1H2BI/HIST1H2BL/HIST1H2BF/HIST1H2BG/HIST1H2BE/DPYSL3/EPHA5/HIST1H4A/HIST1H2BB/LGI2/HIST3H2BB/EPHB1/CACNG4/HIST2H3D/HIST1H2AJ/HIST1H2BM/SEMA3A/HIST1H3D/CD36/HIST1H3F/KLK5 | | |  |
| R-HSA-2262752 | | Cellular responses to stress | | -2.250836856 | 0.001144657 | | | H2AFZ/LMNB1/HIST1H3E/HIST1H2BC/HIST1H3J/UBE2C/CDKN2C/H3F3A/NUP35/HIST1H4L/HIST1H4E/HIST1H2AB/HIST1H2BO/HIST1H3B/HIST1H4H/CCNA2/HIST1H2BJ/HIST1H3G/HIST1H4C/HIST1H4D/HIST2H4A/HIST1H2AE/HIST1H2BN/HIST1H2AD/HIST1H3A/HSPA14/HIST1H2BH/HIST1H4F/HIST1H1B/HIST1H2BI/HIST1H2BL/HIST1H2BF/HIST1H2BG/HIST1H2BE/HIST1H1D/FKBP5/HIST1H4A/HIST1H2BB/HIST3H2BB/HIST2H3D/HIST1H2AJ/HIST1H2BM/HIST1H3D/HIST1H3F | | |  |
| R-HSA-73884 | | Base Excision Repair | | -2.408710438 | 0.001144657 | | | TDG/HIST1H4E/HIST1H2AB/HIST1H2BO/HIST1H4H/HIST1H2BJ/HIST1H4C/HIST1H4D/HIST2H4A/HIST1H2AE/HIST1H2BN/HIST1H2AD/RFC3/HIST1H2BH/POLE4/HIST1H4F/HIST1H2BI/HIST1H2BL/HIST1H2BF/HIST1H2BG/HIST1H2BE/HIST1H4A/HIST1H2BB/HIST3H2BB/HIST1H2AJ/HIST1H2BM | | |  |
| R-HSA-3214858 | | RMTs methylate histone arginines | | -2.38466855 | 0.001144657 | | | HIST1H4B/HIST1H3H/H2AFZ/HIST1H3E/RPS2/HIST1H3J/HIST1H2AL/HIST1H4L/HIST1H4E/HIST1H2AM/HIST1H2AB/HIST1H3B/HIST1H4H/HIST1H3G/HIST1H4C/HIST1H4D/HIST2H4A/HIST1H2AE/HIST1H2AD/HIST1H2AH/HIST1H3A/HIST1H2AI/HIST1H4F/HIST1H4A/HIST2H3D/HIST1H2AJ/HIST1H2AK/HIST1H3D/HIST1H3F | | |  |
| R-HSA-171306 | | Packaging Of Telomere Ends | | -2.46371394 | 0.001144657 | | | HIST1H4B/H2AFZ/HIST1H2BC/HIST1H4L/HIST1H4E/HIST1H2AB/HIST1H2BO/HIST1H4H/HIST1H2BJ/HIST1H4C/HIST1H4D/HIST2H4A/HIST1H2AE/HIST1H2BN/HIST1H2AD/HIST1H2BH/HIST1H4F/HIST1H2BI/HIST1H2BL/HIST1H2BF/HIST1H2BG/HIST1H2BE/HIST1H4A/HIST1H2BB/HIST3H2BB/HIST1H2AJ/HIST1H2BM | | |  |
| R-HSA-5693565 | | Recruitment and ATM-mediated phosphorylation of repair and signaling proteins at DNA double strand breaks | | -2.459900648 | 0.001144657 | | | BARD1/HIST1H4E/HIST1H2BO/HIST1H4H/HIST1H2BJ/HIST1H4C/HIST1H4D/HIST2H4A/HIST1H2BN/HIST1H2BH/HIST1H4F/HIST1H2BI/HIST1H2BL/HIST1H2BF/HIST1H2BG/HIST1H2BE/HIST1H4A/HIST1H2BB/HIST3H2BB/HIST1H2BM/APBB1 | | |  |
| R-HSA-5693606 | | DNA Double Strand Break Response | | -2.519663696 | 0.001144657 | | | BARD1/KPNA2/HIST1H4E/HIST1H2BO/HIST1H4H/HIST1H2BJ/HIST1H4C/HIST1H4D/HIST2H4A/HIST1H2BN/HIST1H2BH/HIST1H4F/HIST1H2BI/HIST1H2BL/HIST1H2BF/HIST1H2BG/HIST1H2BE/HIST1H4A/HIST1H2BB/HIST3H2BB/HIST1H2BM/APBB1 | | |  |
| R-HSA-5693571 | | Nonhomologous End-Joining (NHEJ) | | -2.441464974 | 0.001144657 | | | HIST1H4B/HIST1H2BC/HIST1H4L/BARD1/HIST1H4E/HIST1H2BO/HIST1H4H/HIST1H2BJ/HIST1H4C/HIST1H4D/HIST2H4A/HIST1H2BN/HIST1H2BH/HIST1H4F/HIST1H2BI/HIST1H2BL/HIST1H2BF/HIST1H2BG/HIST1H2BE/HIST1H4A/HIST1H2BB/HIST3H2BB/HIST1H2BM | | |  |
| R-HSA-69473 | | G2/M DNA damage checkpoint | | -2.402294639 | 0.001144657 | | | RMI1/HIST1H4L/BARD1/CCNB1/HIST1H4E/CDK1/HIST1H2BO/WEE1/HIST1H4H/HIST1H2BJ/HIST1H4C/HIST1H4D/HIST2H4A/HIST1H2BN/RAD1/RFC3/HIST1H2BH/HIST1H4F/HIST1H2BI/HIST1H2BL/HIST1H2BF/HIST1H2BG/HIST1H2BE/HIST1H4A/HIST1H2BB/HIST3H2BB/HIST1H2BM | | |  |
| R-HSA-157579 | | Telomere Maintenance | | -2.390153969 | 0.001144657 | | | PRIM1/HIST1H2BD/HIST1H4B/H2AFZ/HIST1H2BC/HIST1H4L/HIST1H4E/HIST1H2AB/HIST1H2BO/HIST1H4H/HIST1H2BJ/HIST1H4C/HIST1H4D/HIST2H4A/HIST1H2AE/HIST1H2BN/HIST1H2AD/RFC3/HIST1H2BH/POLE4/HIST1H4F/HIST1H2BI/HIST1H2BL/HIST1H2BF/HIST1H2BG/HIST1H2BE/HIST1H4A/HIST1H2BB/HIST3H2BB/HIST1H2AJ/HIST1H2BM | | |  |
| R-HSA-110328 | | Recognition and association of DNA glycosylase with site containing an affected pyrimidine | | -2.430526102 | 0.001144657 | | | HIST1H4B/H2AFZ/HIST1H2BC/HIST1H4L/TDG/HIST1H4E/HIST1H2AB/HIST1H2BO/HIST1H4H/HIST1H2BJ/HIST1H4C/HIST1H4D/HIST2H4A/HIST1H2AE/HIST1H2BN/HIST1H2AD/HIST1H2BH/HIST1H4F/HIST1H2BI/HIST1H2BL/HIST1H2BF/HIST1H2BG/HIST1H2BE/HIST1H4A/HIST1H2BB/HIST3H2BB/HIST1H2AJ/HIST1H2BM | | |  |
| R-HSA-110329 | | Cleavage of the damaged pyrimidine | | -2.430526102 | 0.001144657 | | | HIST1H4B/H2AFZ/HIST1H2BC/HIST1H4L/TDG/HIST1H4E/HIST1H2AB/HIST1H2BO/HIST1H4H/HIST1H2BJ/HIST1H4C/HIST1H4D/HIST2H4A/HIST1H2AE/HIST1H2BN/HIST1H2AD/HIST1H2BH/HIST1H4F/HIST1H2BI/HIST1H2BL/HIST1H2BF/HIST1H2BG/HIST1H2BE/HIST1H4A/HIST1H2BB/HIST3H2BB/HIST1H2AJ/HIST1H2BM | | |  |
| R-HSA-73928 | | Depyrimidination | | -2.430526102 | 0.001144657 | | | HIST1H4B/H2AFZ/HIST1H2BC/HIST1H4L/TDG/HIST1H4E/HIST1H2AB/HIST1H2BO/HIST1H4H/HIST1H2BJ/HIST1H4C/HIST1H4D/HIST2H4A/HIST1H2AE/HIST1H2BN/HIST1H2AD/HIST1H2BH/HIST1H4F/HIST1H2BI/HIST1H2BL/HIST1H2BF/HIST1H2BG/HIST1H2BE/HIST1H4A/HIST1H2BB/HIST3H2BB/HIST1H2AJ/HIST1H2BM | | |  |
| R-HSA-73929 | | Base-Excision Repair. AP Site Formation | | -2.430526102 | 0.001144657 | | | HIST1H4B/H2AFZ/HIST1H2BC/HIST1H4L/TDG/HIST1H4E/HIST1H2AB/HIST1H2BO/HIST1H4H/HIST1H2BJ/HIST1H4C/HIST1H4D/HIST2H4A/HIST1H2AE/HIST1H2BN/HIST1H2AD/HIST1H2BH/HIST1H4F/HIST1H2BI/HIST1H2BL/HIST1H2BF/HIST1H2BG/HIST1H2BE/HIST1H4A/HIST1H2BB/HIST3H2BB/HIST1H2AJ/HIST1H2BM | | |  |
| R-HSA-5689880 | | Ub-specific processing proteases | | -2.374539418 | 0.001144657 | | | HIST1H2AB/HIST1H2BO/CCNA2/HIST1H2BJ/SKP2/USP13/HIST1H2AE/HIST1H2BN/HIST1H2AD/HIST2H2BF/HIST1H2AH/HIST1H2AI/HIST1H2BH/HIST1H2BI/HIST1H2BL/HIST1H2BF/HIST1H2BG/HIST1H2BE/HIST1H2BB/HIST3H2BB/HIST1H2AJ/HIST1H2AK/HIST1H2BM | | |  |
| R-HSA-1221632 | | Meiotic synapsis | | -2.352431643 | 0.001144657 | | | HIST1H4B/H2AFZ/LMNB1/HIST1H2BC/HIST1H4L/HIST1H4E/HIST1H2AB/HIST1H2BO/HIST1H4H/HIST1H2BJ/HIST1H4C/HIST1H4D/HIST2H4A/HIST1H2AE/HIST1H2BN/HIST1H2AD/HIST1H2BH/HIST1H4F/HIST1H2BI/HIST1H2BL/HIST1H2BF/HIST1H2BG/HIST1H2BE/HIST1H4A/HIST1H2BB/HIST3H2BB/HIST1H2AJ/HIST1H2BM | | |  |
| R-HSA-5693532 | | DNA Double-Strand Break Repair | | -2.302837724 | 0.001596653 | | | HIST1H2BC/RMI1/HIST1H4L/BARD1/KPNA2/HIST1H4E/HIST1H2BO/HIST1H4H/CCNA2/RAD51AP1/HIST1H2BJ/HIST1H4C/HIST1H4D/HIST2H4A/HIST1H2BN/RAD1/RFC3/HIST1H2BH/POLE4/HIST1H4F/HIST1H2BI/HIST1H2BL/HIST1H2BF/HIST1H2BG/HIST1H2BE/HIST1H4A/HIST1H2BB/HIST3H2BB/HIST1H2BM/APBB1 | | |  |
| R-HSA-2559586 | | DNA Damage/Telomere Stress Induced Senescence | | -2.352833599 | 0.001596653 | | | LMNB1/HIST1H2BC/HIST1H4L/HIST1H4E/HIST1H2AB/HIST1H2BO/HIST1H4H/CCNA2/HIST1H2BJ/HIST1H4C/HIST1H4D/HIST2H4A/HIST1H2AE/HIST1H2BN/HIST1H2AD/HIST1H2BH/HIST1H4F/HIST1H1B/HIST1H2BI/HIST1H2BL/HIST1H2BF/HIST1H2BG/HIST1H2BE/HIST1H1D/HIST1H4A/HIST1H2BB/HIST3H2BB/HIST1H2AJ/HIST1H2BM | | |  |
| R-HSA-110330 | | Recognition and association of DNA glycosylase with site containing an affected purine | | -2.387837373 | 0.001596653 | | | HIST1H4B/H2AFZ/HIST1H2BC/HIST1H4L/HIST1H4E/HIST1H2AB/HIST1H2BO/HIST1H4H/HIST1H2BJ/HIST1H4C/HIST1H4D/HIST2H4A/HIST1H2AE/HIST1H2BN/HIST1H2AD/HIST1H2BH/HIST1H4F/HIST1H2BI/HIST1H2BL/HIST1H2BF/HIST1H2BG/HIST1H2BE/HIST1H4A/HIST1H2BB/HIST3H2BB/HIST1H2AJ/HIST1H2BM | | |  |
| R-HSA-110331 | | Cleavage of the damaged purine | | -2.387837373 | 0.001596653 | | | HIST1H4B/H2AFZ/HIST1H2BC/HIST1H4L/HIST1H4E/HIST1H2AB/HIST1H2BO/HIST1H4H/HIST1H2BJ/HIST1H4C/HIST1H4D/HIST2H4A/HIST1H2AE/HIST1H2BN/HIST1H2AD/HIST1H2BH/HIST1H4F/HIST1H2BI/HIST1H2BL/HIST1H2BF/HIST1H2BG/HIST1H2BE/HIST1H4A/HIST1H2BB/HIST3H2BB/HIST1H2AJ/HIST1H2BM | | |  |
| R-HSA-73927 | | Depurination | | -2.387837373 | 0.001596653 | | | HIST1H4B/H2AFZ/HIST1H2BC/HIST1H4L/HIST1H4E/HIST1H2AB/HIST1H2BO/HIST1H4H/HIST1H2BJ/HIST1H4C/HIST1H4D/HIST2H4A/HIST1H2AE/HIST1H2BN/HIST1H2AD/HIST1H2BH/HIST1H4F/HIST1H2BI/HIST1H2BL/HIST1H2BF/HIST1H2BG/HIST1H2BE/HIST1H4A/HIST1H2BB/HIST3H2BB/HIST1H2AJ/HIST1H2BM | | |  |
| R-HSA-5693607 | | Processing of DNA double-strand break ends | | -2.307118066 | 0.002108578 | | | RMI1/HIST1H4L/BARD1/HIST1H4E/HIST1H2BO/HIST1H4H/CCNA2/HIST1H2BJ/HIST1H4C/HIST1H4D/HIST2H4A/HIST1H2BN/RAD1/RFC3/HIST1H2BH/HIST1H4F/HIST1H2BI/HIST1H2BL/HIST1H2BF/HIST1H2BG/HIST1H2BE/HIST1H4A/HIST1H2BB/HIST3H2BB/HIST1H2BM | | |  |
| R-HSA-8953897 | | Cellular responses to external stimuli | | -2.141427089 | 0.002526785 | | | H2AFZ/LMNB1/HIST1H3E/HIST1H2BC/HIST1H3J/UBE2C/CDKN2C/H3F3A/NUP35/HIST1H4L/HIST1H4E/HIST1H2AB/HIST1H2BO/HIST1H3B/HIST1H4H/CCNA2/HIST1H2BJ/HIST1H3G/HIST1H4C/HIST1H4D/HIST2H4A/HIST1H2AE/HIST1H2BN/HIST1H2AD/HIST1H3A/HSPA14/HIST1H2BH/HIST1H4F/HIST1H1B/HIST1H2BI/HIST1H2BL/HIST1H2BF/HIST1H2BG/HIST1H2BE/HIST1H1D/FKBP5/HIST1H4A/HIST1H2BB/HIST3H2BB/HIST2H3D/HIST1H2AJ/HIST1H2BM/HIST1H3D/HIST1H3F | | |  |
| R-HSA-5688426 | | Deubiquitination | | -2.188054951 | 0.00304659 | | | BARD1/CDK1/HIST1H2AM/HIST1H2AB/HIST1H2BO/CCNA2/HIST1H2BJ/SKP2/USP13/HIST1H2AE/HIST1H2BN/HIST1H2AD/HIST2H2BF/HIST1H2AH/HIST1H2AI/HIST1H2BH/HIST1H2BI/HIST1H2BL/HIST1H2BF/HIST1H2BG/HIST1H2BE/HIST1H2BB/HIST3H2BB/HIST1H2AJ/HIST1H2AK/HIST1H2BM | | |  |
| R-HSA-3214842 | | HDMs demethylate histones | | -2.170616081 | 0.003543324 | | | HIST1H3C/HIST1H4B/HIST1H3H/HIST1H3E/HIST1H3J/HIST1H4L/HIST1H4E/ARID5B/HIST1H3B/HIST1H4H/HIST1H3G/HIST1H4C/HIST1H4D/HIST2H4A/HIST1H3A/HIST1H4F/HIST1H4A/HIST2H3D/HIST1H3D/HIST1H3F | | |  |
| R-HSA-8866654 | | E3 ubiquitin ligases ubiquitinate target proteins | | -2.139498825 | 0.003543324 | | | HLA-A/HIST1H2BC/HIST1H2BO/HIST1H2BJ/HIST1H2BN/HIST1H2BH/HIST1H2BI/HIST1H2BL/HIST1H2BF/HIST1H2BG/HIST1H2BE/HIST1H2BB/HIST1H2BM | | |  |
| R-HSA-5693567 | | HDR through Homologous Recombination (HRR) or Single Strand Annealing (SSA) | | -2.215928311 | 0.003863968 | | | HIST1H2BC/RMI1/HIST1H4L/BARD1/HIST1H4E/HIST1H2BO/HIST1H4H/CCNA2/RAD51AP1/HIST1H2BJ/HIST1H4C/HIST1H4D/HIST2H4A/HIST1H2BN/RAD1/RFC3/HIST1H2BH/POLE4/HIST1H4F/HIST1H2BI/HIST1H2BL/HIST1H2BF/HIST1H2BG/HIST1H2BE/HIST1H4A/HIST1H2BB/HIST3H2BB/HIST1H2BM | | |  |
| R-HSA-5693538 | | Homology Directed Repair | | -2.15212998 | 0.003863968 | | | HIST1H2BC/RMI1/HIST1H4L/BARD1/HIST1H4E/HIST1H2BO/HIST1H4H/CCNA2/RAD51AP1/HIST1H2BJ/HIST1H4C/HIST1H4D/HIST2H4A/HIST1H2BN/RAD1/RFC3/HIST1H2BH/POLE4/HIST1H4F/HIST1H2BI/HIST1H2BL/HIST1H2BF/HIST1H2BG/HIST1H2BE/HIST1H4A/HIST1H2BB/HIST3H2BB/HIST1H2BM | | |  |
| R-HSA-8852135 | | Protein ubiquitination | | -2.085847218 | 0.004876345 | | | HLA-A/HIST1H2BC/UBE2C/HIST1H2BO/HIST1H2BJ/HIST1H2BN/HIST1H2BH/HIST1H2BI/HIST1H2BL/HIST1H2BF/HIST1H2BG/HIST1H2BE/HIST1H2BB/HIST1H2BM | | |  |
| R-HSA-73894 | | DNA Repair | | -1.992282183 | 0.008346901 | | | XRCC2/HIST1H2BC/USP1/RMI1/HIST1H4L/BARD1/TDG/KPNA2/HIST1H4E/HIST1H2AB/HIST1H2BO/HIST1H4H/CCNA2/RAD51AP1/HIST1H2BJ/HIST1H4C/HIST1H4D/HIST2H4A/HIST1H2AE/HIST1H2BN/RAD1/HIST1H2AD/RFC3/HIST1H2BH/POLE4/HIST1H4F/HIST1H2BI/HIST1H2BL/HIST1H2BF/HIST1H2BG/HIST1H2BE/HIST1H4A/HIST1H2BB/HIST3H2BB/HIST1H2AJ/HIST1H2BM/APBB1 | | |  |

| **Supplementary Table S2: Deregulated pathways in sarcomatoid CD60 cells at 24h** | | | | | |
| --- | --- | --- | --- | --- | --- |
| **ID** | **Pathway name** | **NES** | **p.adjust** | **Genes in pathway** |  |
| R-HSA-195258 | RHO GTPase Effectors | -2.279927696 | 0.007346736 | HIST2H3D/HIST1H2AJ/HIST1H2BH/HIST3H2BB/HIST1H3D/PRKCZ/HIST1H3F |  |
| R-HSA-194315 | Signaling by Rho GTPases | -2.21325473 | 0.007346736 | HIST2H3D/HIST1H2AJ/HIST1H2BH/HIST3H2BB/HIST1H3D/PRKCZ/HIST1H3F |  |
| R-HSA-1280215 | Cytokine Signaling in Immune system | -2.157837014 | 0.007346736 | SERPINB2/HIST2H3D/HLA-F/IL21R/HIST1H3D/HIST1H3F |  |
| R-HSA-5617472 | Activation of anterior HOX genes in hindbrain development during early embryogenesis | -2.138874875 | 0.007346736 | HIST2H3D/HIST1H2AJ/HIST1H2BH/HOXA3/HIST3H2BB/HIST1H3D/HIST1H3F |  |
| R-HSA-5619507 | Activation of HOX genes during differentiation | -2.138874875 | 0.007346736 | HIST2H3D/HIST1H2AJ/HIST1H2BH/HOXA3/HIST3H2BB/HIST1H3D/HIST1H3F |  |
| R-HSA-8939211 | ESR-mediated signaling | -2.103202013 | 0.007346736 | HIST2H3D/HIST1H2AJ/HIST1H2BH/HIST3H2BB/HIST1H3D/PRKCZ/HIST1H3F |  |
| R-HSA-977225 | Amyloid fiber formation | -2.136468526 | 0.007346736 | TGFBI/HIST1H4A/HIST2H3D/HIST1H2AJ/HIST1H2BH/HIST3H2BB/HIST1H3D/HIST1H3F |  |
| R-HSA-3247509 | Chromatin modifying enzymes | -2.136979941 | 0.007346736 | HIST1H2AK/HIST1H4A/HIST2H3D/HIST1H2AJ/HIST1H2BH/HIST3H2BB/HIST1H3D/HIST1H3F |  |
| R-HSA-4839726 | Chromatin organization | -2.136979941 | 0.007346736 | HIST1H2AK/HIST1H4A/HIST2H3D/HIST1H2AJ/HIST1H2BH/HIST3H2BB/HIST1H3D/HIST1H3F |  |
| R-HSA-3214815 | HDACs deacetylate histones | -2.136979941 | 0.007346736 | HIST1H2AK/HIST1H4A/HIST2H3D/HIST1H2AJ/HIST1H2BH/HIST3H2BB/HIST1H3D/HIST1H3F |  |
| R-HSA-3214847 | HATs acetylate histones | -2.136979941 | 0.007346736 | HIST1H2AK/HIST1H4A/HIST2H3D/HIST1H2AJ/HIST1H2BH/HIST3H2BB/HIST1H3D/HIST1H3F |  |
| R-HSA-1912408 | Pre-NOTCH Transcription and Translation | -2.051890155 | 0.007346736 | HIST2H3D/HIST1H2AJ/HIST1H2BH/HIST3H2BB/HIST1H3D/HIST1H3F |  |
| R-HSA-1912422 | Pre-NOTCH Expression and Processing | -2.051890155 | 0.007346736 | HIST2H3D/HIST1H2AJ/HIST1H2BH/HIST3H2BB/HIST1H3D/HIST1H3F |  |
| R-HSA-2299718 | Condensation of Prophase Chromosomes | -2.051890155 | 0.007346736 | HIST2H3D/HIST1H2AJ/HIST1H2BH/HIST3H2BB/HIST1H3D/HIST1H3F |  |
| R-HSA-68875 | Mitotic Prophase | -2.051890155 | 0.007346736 | HIST2H3D/HIST1H2AJ/HIST1H2BH/HIST3H2BB/HIST1H3D/HIST1H3F |  |
| R-HSA-2559580 | Oxidative Stress Induced Senescence | -2.051890155 | 0.007346736 | HIST2H3D/HIST1H2AJ/HIST1H2BH/HIST3H2BB/HIST1H3D/HIST1H3F |  |
| R-HSA-2559583 | Cellular Senescence | -2.051890155 | 0.007346736 | HIST2H3D/HIST1H2AJ/HIST1H2BH/HIST3H2BB/HIST1H3D/HIST1H3F |  |
| R-HSA-212165 | Epigenetic regulation of gene expression | -2.051890155 | 0.007346736 | HIST2H3D/HIST1H2AJ/HIST1H2BH/HIST3H2BB/HIST1H3D/HIST1H3F |  |
| R-HSA-427413 | NoRC negatively regulates rRNA expression | -2.051890155 | 0.007346736 | HIST2H3D/HIST1H2AJ/HIST1H2BH/HIST3H2BB/HIST1H3D/HIST1H3F |  |
| R-HSA-5250941 | Negative epigenetic regulation of rRNA expression | -2.051890155 | 0.007346736 | HIST2H3D/HIST1H2AJ/HIST1H2BH/HIST3H2BB/HIST1H3D/HIST1H3F |  |
| R-HSA-73864 | RNA Polymerase I Transcription | -2.051890155 | 0.007346736 | HIST2H3D/HIST1H2AJ/HIST1H2BH/HIST3H2BB/HIST1H3D/HIST1H3F |  |
| R-HSA-2559582 | Senescence-Associated Secretory Phenotype (SASP) | -2.051890155 | 0.007346736 | HIST2H3D/HIST1H2AJ/HIST1H2BH/HIST3H2BB/HIST1H3D/HIST1H3F |  |
| R-HSA-73772 | RNA Polymerase I Promoter Escape | -2.051890155 | 0.007346736 | HIST2H3D/HIST1H2AJ/HIST1H2BH/HIST3H2BB/HIST1H3D/HIST1H3F |  |
| R-HSA-73854 | RNA Polymerase I Promoter Clearance | -2.051890155 | 0.007346736 | HIST2H3D/HIST1H2AJ/HIST1H2BH/HIST3H2BB/HIST1H3D/HIST1H3F |  |
| R-HSA-212300 | PRC2 methylates histones and DNA | -2.051890155 | 0.007346736 | HIST2H3D/HIST1H2AJ/HIST1H2BH/HIST3H2BB/HIST1H3D/HIST1H3F |  |
| R-HSA-427359 | SIRT1 negatively regulates rRNA expression | -2.051890155 | 0.007346736 | HIST2H3D/HIST1H2AJ/HIST1H2BH/HIST3H2BB/HIST1H3D/HIST1H3F |  |
| R-HSA-427389 | ERCC6 (CSB) and EHMT2 (G9a) positively regulate rRNA expression | -2.051890155 | 0.007346736 | HIST2H3D/HIST1H2AJ/HIST1H2BH/HIST3H2BB/HIST1H3D/HIST1H3F |  |
| R-HSA-5250913 | Positive epigenetic regulation of rRNA expression | -2.051890155 | 0.007346736 | HIST2H3D/HIST1H2AJ/HIST1H2BH/HIST3H2BB/HIST1H3D/HIST1H3F |  |
| R-HSA-5250924 | B-WICH complex positively regulates rRNA expression | -2.051890155 | 0.007346736 | HIST2H3D/HIST1H2AJ/HIST1H2BH/HIST3H2BB/HIST1H3D/HIST1H3F |  |
| R-HSA-5334118 | DNA methylation | -2.051890155 | 0.007346736 | HIST2H3D/HIST1H2AJ/HIST1H2BH/HIST3H2BB/HIST1H3D/HIST1H3F |  |
| R-HSA-5578749 | Transcriptional regulation by small RNAs | -2.051890155 | 0.007346736 | HIST2H3D/HIST1H2AJ/HIST1H2BH/HIST3H2BB/HIST1H3D/HIST1H3F |  |
| R-HSA-5625740 | RHO GTPases activate PKNs | -2.051890155 | 0.007346736 | HIST2H3D/HIST1H2AJ/HIST1H2BH/HIST3H2BB/HIST1H3D/HIST1H3F |  |
| R-HSA-5625886 | Activated PKN1 stimulates transcription of AR (androgen receptor) regulated genes KLK2 and KLK3 | -2.051890155 | 0.007346736 | HIST2H3D/HIST1H2AJ/HIST1H2BH/HIST3H2BB/HIST1H3D/HIST1H3F |  |
| R-HSA-73728 | RNA Polymerase I Promoter Opening | -2.051890155 | 0.007346736 | HIST2H3D/HIST1H2AJ/HIST1H2BH/HIST3H2BB/HIST1H3D/HIST1H3F |  |
| R-HSA-1266738 | Developmental Biology | -1.952616322 | 0.009944009 | HIST1H4A/HIST2H3D/HIST1H2AJ/HIST1H2BH/UNC5B/HOXA3/CACNG4/HIST3H2BB/HIST1H3D/HIST1H3F |  |
| R-HSA-68886 | M Phase | -2.009942208 | 0.009944009 | HIST2H3D/HIST1H2AJ/HIST1H2BH/HIST3H2BB/HIST1H3D/HIST1H3F |  |
| R-HSA-8939236 | RUNX1 regulates transcription of genes involved in differentiation of HSCs | -2.009942208 | 0.009944009 | HIST2H3D/HIST1H2AJ/HIST1H2BH/HIST3H2BB/HIST1H3D/HIST1H3F |  |
| R-HSA-8936459 | RUNX1 regulates genes involved in megakaryocyte differentiation and platelet function | -2.009305523 | 0.009944009 | HIST2H3D/HIST1H2AJ/HIST1H2BH/HIST3H2BB/HIST1H3D/HIST1H3F |  |
