# Supplementary material for: Tumor treating fields affect mesothelioma cell proliferation by exerting histotype-dependent cell cycle checkpoint activations and transcriptional modulations
Source: Cell Death Dis. 2022 Jul 15;13(7):612. doi: 10.1038/s41419-022-05073-4 (PMC9287343; doi:10.1038/s41419-022-05073-4)
Supplement: Supplementary file 4 — Supplementary Table 3 [file 41419_2022_5073_MOESM4_ESM.docx]

| **Supplementary Table S3: Pathways that were exclusively modulated in the epithelioid subtype.** | | | | |
| --- | --- | --- | --- | --- |
| **ID** | **Description** | **p.adjust** | **Count** | **geneID** |
| R-HSA-68882 | Mitotic Anaphase | 4.09230865620927e-12 | 54 | ANAPC16/AURKB/BUB3/CCNB1/CCNB2/CDK1/CENPA/CENPF/CHMP2B/CHMP4A/CKAP5/DSN1/ERCC6L/ESPL1/INCENP/KIF2C/KNL1/LBR/MAD2L1/MIS12/NDC1/NDC80/NUF2/NUP205/NUP35/NUP37/NUP43/NUP54/NUP98/PDS5A/POM121/PPP1CC/PPP2R1B/PPP2R2A/PPP2R5A/PSMA3/PSMA4/PSMB3/PSMD10/PSME2/PTTG1/RCC1/SEM1/SGO1/SGO2/SIRT2/SUMO1/TMPO/TUBB8/UBE2C/UBE2D1/VRK2/WAPL/ZW10 |
| R-HSA-2555396 | Mitotic Metaphase and Anaphase | 4.09230865620927e-12 | 54 | ANAPC16/AURKB/BUB3/CCNB1/CCNB2/CDK1/CENPA/CENPF/CHMP2B/CHMP4A/CKAP5/DSN1/ERCC6L/ESPL1/INCENP/KIF2C/KNL1/LBR/MAD2L1/MIS12/NDC1/NDC80/NUF2/NUP205/NUP35/NUP37/NUP43/NUP54/NUP98/PDS5A/POM121/PPP1CC/PPP2R1B/PPP2R2A/PPP2R5A/PSMA3/PSMA4/PSMB3/PSMD10/PSME2/PTTG1/RCC1/SEM1/SGO1/SGO2/SIRT2/SUMO1/TMPO/TUBB8/UBE2C/UBE2D1/VRK2/WAPL/ZW10 |
| R-HSA-68886 | M Phase | 1.19732127041472e-10 | 73 | ANAPC16/AURKB/BUB3/CCNB1/CCNB2/CDK1/CENPA/CENPF/CEP70/CEP78/CHMP2B/CHMP4A/CKAP5/CNEP1R1/CTDNEP1/DSN1/ERCC6L/ESPL1/H2AC7/H2AZ1/H4C3/H4C8/INCENP/KIF20A/KIF23/KIF2C/KNL1/LBR/MAD2L1/MAU2/MIS12/MZT1/NCAPD3/NCAPG/NDC1/NDC80/NEDD1/NEK2/NUF2/NUP205/NUP35/NUP37/NUP43/NUP54/NUP98/PDS5A/POM121/PPP1CC/PPP2R1B/PPP2R2A/PPP2R5A/PRKAR2B/PSMA3/PSMA4/PSMB3/PSMD10/PSME2/PTTG1/RCC1/SEM1/SGO1/SGO2/SIRT2/SMC2/SUMO1/TMPO/TPR/TUBB8/UBE2C/UBE2D1/VRK2/WAPL/ZW10 |
| R-HSA-69620 | Cell Cycle Checkpoints | 5.80538392073457e-08 | 53 | ANAPC16/AURKB/BUB3/CCNA2/CCNB1/CCNB2/CDC25C/CDC7/CDK1/CDKN2A/CENPA/CENPF/CHEK1/CKAP5/DBF4/DNA2/DSN1/ERCC6L/H4C3/H4C8/INCENP/KIF2C/KNL1/MAD2L1/MCM8/MDC1/MIS12/NBN/NDC80/NUF2/NUP37/NUP43/NUP98/PPP1CC/PPP2R1B/PPP2R5A/PSMA3/PSMA4/PSMB3/PSMD10/PSME2/RAD1/RAD50/RPA3/SEM1/SGO1/SGO2/SUMO1/UBE2C/UBE2D1/UBE2V2/WEE1/ZW10 |
| R-HSA-2500257 | Resolution of Sister Chromatid Cohesion | 2.64997721392165e-06 | 29 | AURKB/BUB3/CCNB1/CCNB2/CDK1/CENPA/CENPF/CKAP5/DSN1/ERCC6L/INCENP/KIF2C/KNL1/MAD2L1/MIS12/NDC80/NUF2/NUP37/NUP43/NUP98/PDS5A/PPP1CC/PPP2R1B/PPP2R5A/SGO1/SGO2/TUBB8/WAPL/ZW10 |
| R-HSA-2467813 | Separation of Sister Chromatids | 3.23427797341746e-06 | 37 | ANAPC16/AURKB/BUB3/CENPA/CENPF/CKAP5/DSN1/ERCC6L/ESPL1/INCENP/KIF2C/KNL1/MAD2L1/MIS12/NDC80/NUF2/NUP37/NUP43/NUP98/PDS5A/PPP1CC/PPP2R1B/PPP2R5A/PSMA3/PSMA4/PSMB3/PSMD10/PSME2/PTTG1/SEM1/SGO1/SGO2/TUBB8/UBE2C/UBE2D1/WAPL/ZW10 |
| R-HSA-2995410 | Nuclear Envelope (NE) Reassembly | 5.50612668746575e-06 | 21 | CCNB1/CCNB2/CDK1/CHMP2B/CHMP4A/LBR/NDC1/NUP205/NUP35/NUP37/NUP43/NUP54/NUP98/POM121/PPP2R2A/RCC1/SIRT2/SUMO1/TMPO/TUBB8/VRK2 |
| R-HSA-69618 | Mitotic Spindle Checkpoint | 9.65491349927417e-06 | 26 | ANAPC16/AURKB/BUB3/CENPA/CENPF/CKAP5/DSN1/ERCC6L/INCENP/KIF2C/KNL1/MAD2L1/MIS12/NDC80/NUF2/NUP37/NUP43/NUP98/PPP1CC/PPP2R1B/PPP2R5A/SGO1/SGO2/UBE2C/UBE2D1/ZW10 |
| R-HSA-1428517 | The citric acid (TCA) cycle and respiratory electron transport | 1.17557012918636e-05 | 34 | ATP5F1A/ATP5F1B/ATP5MC2/ATP5MC3/ATP5MF/ATP5MG/ATP5PB/ATP5PO/COX14/COX7A2L/COX7B/COX7C/CYCS/DLD/ETFA/FH/NDUFA1/NDUFA11/NDUFA5/NDUFA9/NDUFB11/NDUFB2/NDUFS5/NNT/OGDH/PDHA1/PDHX/PDP1/SDHD/SUCLG1/UCP2/UQCRFS1/UQCRQ/VDAC1 |
| R-HSA-68877 | Mitotic Prometaphase | 1.17557012918636e-05 | 37 | AURKB/BUB3/CCNB1/CCNB2/CDK1/CENPA/CENPF/CEP70/CEP78/CKAP5/DSN1/ERCC6L/INCENP/KIF2C/KNL1/MAD2L1/MIS12/MZT1/NCAPG/NDC80/NEDD1/NEK2/NUF2/NUP37/NUP43/NUP98/PDS5A/PPP1CC/PPP2R1B/PPP2R5A/PRKAR2B/SGO1/SGO2/SMC2/TUBB8/WAPL/ZW10 |
| R-HSA-141424 | Amplification of signal from the kinetochores | 1.69899242093089e-05 | 23 | AURKB/BUB3/CENPA/CENPF/CKAP5/DSN1/ERCC6L/INCENP/KIF2C/KNL1/MAD2L1/MIS12/NDC80/NUF2/NUP37/NUP43/NUP98/PPP1CC/PPP2R1B/PPP2R5A/SGO1/SGO2/ZW10 |
| R-HSA-141444 | Amplification of signal from unattached kinetochores via a MAD2 inhibitory signal | 1.69899242093089e-05 | 23 | AURKB/BUB3/CENPA/CENPF/CKAP5/DSN1/ERCC6L/INCENP/KIF2C/KNL1/MAD2L1/MIS12/NDC80/NUF2/NUP37/NUP43/NUP98/PPP1CC/PPP2R1B/PPP2R5A/SGO1/SGO2/ZW10 |
| R-HSA-4615885 | SUMOylation of DNA replication proteins | 2.29408184323365e-05 | 15 | AURKA/AURKB/INCENP/NDC1/NUP205/NUP35/NUP37/NUP43/NUP54/NUP98/PCNA/POM121/SUMO1/TOP2A/TPR |
| R-HSA-2980766 | Nuclear Envelope Breakdown | 2.95534684634262e-05 | 16 | CCNB1/CCNB2/CDK1/CNEP1R1/CTDNEP1/NDC1/NUP205/NUP35/NUP37/NUP43/NUP54/NUP98/POM121/TMPO/TPR/VRK2 |
| R-HSA-9648025 | EML4 and NUDC in mitotic spindle formation | 0.000154175390635085 | 24 | AURKB/BUB3/CENPA/CENPF/CKAP5/DSN1/ERCC6L/INCENP/KIF2C/KNL1/MAD2L1/MIS12/NDC80/NUF2/NUP37/NUP43/NUP98/PPP1CC/PPP2R1B/PPP2R5A/SGO1/SGO2/TUBB8/ZW10 |
| R-HSA-163200 | Respiratory electron transport, ATP synthesis by chemiosmotic coupling, and heat production by uncoupling proteins. | 0.000175830554898732 | 25 | ATP5F1A/ATP5F1B/ATP5MC2/ATP5MC3/ATP5MF/ATP5MG/ATP5PB/ATP5PO/COX14/COX7A2L/COX7B/COX7C/CYCS/ETFA/NDUFA1/NDUFA11/NDUFA5/NDUFA9/NDUFB11/NDUFB2/NDUFS5/SDHD/UCP2/UQCRFS1/UQCRQ |
| R-HSA-191859 | snRNP Assembly | 0.000175830554898732 | 15 | GEMIN2/NCBP2/NDC1/NUP205/NUP35/NUP37/NUP43/NUP54/NUP98/POM121/SNRPB/SNRPD2/SNRPE/SNRPG/TPR |
| R-HSA-194441 | Metabolism of non-coding RNA | 0.000175830554898732 | 15 | GEMIN2/NCBP2/NDC1/NUP205/NUP35/NUP37/NUP43/NUP54/NUP98/POM121/SNRPB/SNRPD2/SNRPE/SNRPG/TPR |
| R-HSA-3301854 | Nuclear Pore Complex (NPC) Disassembly | 0.00018302457091174 | 12 | CCNB1/CCNB2/CDK1/NDC1/NUP205/NUP35/NUP37/NUP43/NUP54/NUP98/POM121/TPR |
| R-HSA-156711 | Polo-like kinase mediated events | 0.000306941569842991 | 8 | CCNB1/CCNB2/CDC25C/CENPF/LIN52/LIN9/RBBP4/WEE1 |
| R-HSA-2990846 | SUMOylation | 0.000489802227769594 | 31 | AR/AURKA/AURKB/CDKN2A/DDX17/H4C3/H4C8/HIPK2/HNRNPC/IKBKE/INCENP/MDC1/NCOR2/NDC1/NR3C1/NR3C2/NSMCE2/NUP205/NUP35/NUP37/NUP43/NUP54/NUP98/PCNA/POM121/PPARG/SENP2/SUMO1/TDG/TOP2A/TPR |
| R-HSA-9615933 | Postmitotic nuclear pore complex (NPC) reformation | 0.000493861934516494 | 10 | NDC1/NUP205/NUP35/NUP37/NUP43/NUP54/NUP98/POM121/RCC1/SUMO1 |
| R-HSA-174143 | APC/C-mediated degradation of cell cycle proteins | 0.000523442968508694 | 19 | ANAPC16/AURKA/AURKB/BUB3/CCNA2/CCNB1/CDK1/MAD2L1/NEK2/PSMA3/PSMA4/PSMB3/PSMD10/PSME2/PTTG1/SEM1/SKP2/UBE2C/UBE2D1 |
| R-HSA-453276 | Regulation of mitotic cell cycle | 0.000523442968508694 | 19 | ANAPC16/AURKA/AURKB/BUB3/CCNA2/CCNB1/CDK1/MAD2L1/NEK2/PSMA3/PSMA4/PSMB3/PSMD10/PSME2/PTTG1/SEM1/SKP2/UBE2C/UBE2D1 |
| R-HSA-3108232 | SUMO E3 ligases SUMOylate target proteins | 0.000578765128812301 | 30 | AR/AURKA/AURKB/CDKN2A/DDX17/H4C3/H4C8/HIPK2/HNRNPC/IKBKE/INCENP/MDC1/NCOR2/NDC1/NR3C1/NR3C2/NSMCE2/NUP205/NUP35/NUP37/NUP43/NUP54/NUP98/PCNA/POM121/PPARG/SUMO1/TDG/TOP2A/TPR |
| R-HSA-159227 | Transport of the SLBP independent Mature mRNA | 0.000662905757673712 | 11 | EIF4E/NCBP2/NDC1/NUP205/NUP35/NUP37/NUP43/NUP54/NUP98/POM121/TPR |
| R-HSA-163210 | Formation of ATP by chemiosmotic coupling | 0.000679608070793562 | 8 | ATP5F1A/ATP5F1B/ATP5MC2/ATP5MC3/ATP5MF/ATP5MG/ATP5PB/ATP5PO |
| R-HSA-5663220 | RHO GTPases Activate Formins | 0.000679608070793562 | 25 | AURKB/BUB3/CENPA/CENPF/CKAP5/DIAPH1/DSN1/ERCC6L/INCENP/KIF2C/KNL1/MAD2L1/MIS12/NDC80/NUF2/NUP37/NUP43/NUP98/PPP1CC/PPP2R1B/PPP2R5A/SGO1/SGO2/TUBB8/ZW10 |
| R-HSA-453279 | Mitotic G1 phase and G1/S transition | 0.000681293889479986 | 26 | CCNA2/CCNB1/CDC7/CDK1/CDKN2A/CDKN2C/CKS1B/DBF4/LIN52/LIN9/MCM8/PCNA/POLE4/PPP2R1B/PPP2R2A/PSMA3/PSMA4/PSMB3/PSMD10/PSME2/RBBP4/RPA3/SEM1/SKP2/TOP2A/WEE1 |
| R-HSA-159231 | Transport of Mature mRNA Derived from an Intronless Transcript | 0.000738646341736242 | 12 | CPSF2/EIF4E/NCBP2/NDC1/NUP205/NUP35/NUP37/NUP43/NUP54/NUP98/POM121/TPR |
| R-HSA-159230 | Transport of the SLBP Dependant Mature mRNA | 0.000759397989465756 | 11 | EIF4E/NCBP2/NDC1/NUP205/NUP35/NUP37/NUP43/NUP54/NUP98/POM121/TPR |
| R-HSA-72203 | Processing of Capped Intron-Containing Pre-mRNA | 0.000803794846773505 | 36 | CDC5L/CPSF2/DHX15/DNAJC8/EIF4E/GTF2F2/HNRNPC/HNRNPF/LSM2/LSM3/LSM4/LSM5/LSM6/MAGOHB/NCBP2/NDC1/NUDT21/NUP205/NUP35/NUP37/NUP43/NUP54/NUP98/PCBP1/POLR2A/POM121/PPWD1/SNRPB/SNRPC/SNRPD2/SNRPE/SNRPG/SRSF1/SRSF10/TPR/ZCRB1 |
| R-HSA-159234 | Transport of Mature mRNAs Derived from Intronless Transcripts | 0.000875332543246837 | 12 | CPSF2/EIF4E/NCBP2/NDC1/NUP205/NUP35/NUP37/NUP43/NUP54/NUP98/POM121/TPR |
| R-HSA-2995383 | Initiation of Nuclear Envelope (NE) Reformation | 0.000875332543246837 | 8 | CCNB1/CCNB2/CDK1/LBR/PPP2R2A/SIRT2/TMPO/VRK2 |
| R-HSA-176033 | Interactions of Vpr with host cellular proteins | 0.000907172606435864 | 11 | NDC1/NUP205/NUP35/NUP37/NUP43/NUP54/NUP98/POM121/PSIP1/SLC25A4/TPR |
| R-HSA-180746 | Nuclear import of Rev protein | 0.0021811084101922 | 10 | NDC1/NUP205/NUP35/NUP37/NUP43/NUP54/NUP98/POM121/RCC1/TPR |
| R-HSA-180910 | Vpr-mediated nuclear import of PICs | 0.0021811084101922 | 10 | NDC1/NUP205/NUP35/NUP37/NUP43/NUP54/NUP98/POM121/PSIP1/TPR |
| R-HSA-176409 | APC/C:Cdc20 mediated degradation of mitotic proteins | 0.00224673344863896 | 16 | ANAPC16/BUB3/CCNA2/CCNB1/CDK1/MAD2L1/NEK2/PSMA3/PSMA4/PSMB3/PSMD10/PSME2/PTTG1/SEM1/UBE2C/UBE2D1 |
| R-HSA-69242 | S Phase | 0.00224673344863896 | 26 | ANAPC16/CCNA2/CKS1B/DNA2/ESCO2/GINS1/LIN52/LIN9/MCM8/PCNA/PDS5A/POLD3/POLE4/PSMA3/PSMA4/PSMB3/PSMD10/PSME2/RBBP4/RPA3/SEM1/SKP2/UBE2C/UBE2D1/WAPL/WEE1 |
| R-HSA-176814 | Activation of APC/C and APC/C:Cdc20 mediated degradation of mitotic proteins | 0.00255107032046292 | 16 | ANAPC16/BUB3/CCNA2/CCNB1/CDK1/MAD2L1/NEK2/PSMA3/PSMA4/PSMB3/PSMD10/PSME2/PTTG1/SEM1/UBE2C/UBE2D1 |
| R-HSA-165054 | Rev-mediated nuclear export of HIV RNA | 0.00255107032046292 | 10 | NDC1/NUP205/NUP35/NUP37/NUP43/NUP54/NUP98/POM121/RCC1/TPR |
| R-HSA-4085377 | SUMOylation of SUMOylation proteins | 0.00255107032046292 | 10 | NDC1/NUP205/NUP35/NUP37/NUP43/NUP54/NUP98/POM121/SUMO1/TPR |
| R-HSA-69275 | G2/M Transition | 0.00358171070175976 | 29 | AJUBA/AURKA/BORA/CCNA2/CCNB1/CCNB2/CDC25C/CDK1/CENPF/CEP70/CEP78/CKAP5/LIN52/LIN9/MZT1/NEDD1/NEK2/PPP2R1B/PPP2R2A/PRKAR2B/PSMA3/PSMA4/PSMB3/PSMD10/PSME2/RBBP4/SEM1/TUBB8/WEE1 |
| R-HSA-69206 | G1/S Transition | 0.0036009420050816 | 22 | CCNA2/CCNB1/CDC7/CDK1/CKS1B/DBF4/LIN52/LIN9/MCM8/PCNA/POLE4/PPP2R1B/PSMA3/PSMA4/PSMB3/PSMD10/PSME2/RBBP4/RPA3/SEM1/SKP2/WEE1 |
| R-HSA-177243 | Interactions of Rev with host cellular proteins | 0.00401539752195436 | 10 | NDC1/NUP205/NUP35/NUP37/NUP43/NUP54/NUP98/POM121/RCC1/TPR |
| R-HSA-453274 | Mitotic G2-G2/M phases | 0.00401539752195436 | 29 | AJUBA/AURKA/BORA/CCNA2/CCNB1/CCNB2/CDC25C/CDK1/CENPF/CEP70/CEP78/CKAP5/LIN52/LIN9/MZT1/NEDD1/NEK2/PPP2R1B/PPP2R2A/PRKAR2B/PSMA3/PSMA4/PSMB3/PSMD10/PSME2/RBBP4/SEM1/TUBB8/WEE1 |
| R-HSA-168325 | Viral Messenger RNA Synthesis | 0.00409387506870392 | 11 | GTF2F2/NDC1/NUP205/NUP35/NUP37/NUP43/NUP54/NUP98/POLR2A/POM121/TPR |
| R-HSA-68875 | Mitotic Prophase | 0.00410259744245533 | 23 | CCNB1/CCNB2/CDK1/CNEP1R1/CTDNEP1/H2AC7/H2AZ1/H4C3/H4C8/NCAPD3/NDC1/NUP205/NUP35/NUP37/NUP43/NUP54/NUP98/POM121/PPP2R1B/SMC2/TMPO/TPR/VRK2 |
| R-HSA-70326 | Glucose metabolism | 0.00503211286633107 | 17 | GNPDA1/MDH1/NDC1/NUP205/NUP35/NUP37/NUP43/NUP54/NUP98/PFKP/PGAM1/PGK1/POM121/PPP2R1B/SLC25A1/SLC37A4/TPR |
| R-HSA-168271 | Transport of Ribonucleoproteins into the Host Nucleus | 0.00503211286633107 | 9 | NDC1/NUP205/NUP35/NUP37/NUP43/NUP54/NUP98/POM121/TPR |
| R-HSA-168333 | NEP/NS2 Interacts with the Cellular Export Machinery | 0.00503211286633107 | 9 | NDC1/NUP205/NUP35/NUP37/NUP43/NUP54/NUP98/POM121/TPR |
| R-HSA-170822 | Regulation of Glucokinase by Glucokinase Regulatory Protein | 0.00503211286633107 | 9 | NDC1/NUP205/NUP35/NUP37/NUP43/NUP54/NUP98/POM121/TPR |
| R-HSA-5619107 | Defective TPR may confer susceptibility towards thyroid papillary carcinoma (TPC) | 0.00503211286633107 | 9 | NDC1/NUP205/NUP35/NUP37/NUP43/NUP54/NUP98/POM121/TPR |
| R-HSA-8949613 | Cristae formation | 0.00503211286633107 | 9 | ATP5F1A/ATP5F1B/ATP5MC2/ATP5MC3/ATP5MF/ATP5MG/ATP5PB/ATP5PO/CHCHD3 |
| R-HSA-69273 | Cyclin A/B1/B2 associated events during G2/M transition | 0.00528502391289066 | 8 | CCNA2/CCNB1/CCNB2/CDC25C/CDK1/PPP2R1B/PPP2R2A/WEE1 |
| R-HSA-3232142 | SUMOylation of ubiquitinylation proteins | 0.00532343120316538 | 10 | NDC1/NUP205/NUP35/NUP37/NUP43/NUP54/NUP98/POM121/SUMO1/TPR |
| R-HSA-195258 | RHO GTPase Effectors | 0.00542462047273688 | 41 | AR/AURKB/BUB3/CALM3/CDC25C/CENPA/CENPF/CKAP5/DIAPH1/DSN1/ERCC6L/GOPC/H2AC7/H2AZ1/H4C3/H4C8/INCENP/KIF14/KIF2C/KNL1/LIMK1/MAD2L1/MIS12/MYLK/NCKAP1/NDC80/NUF2/NUP37/NUP43/NUP98/PPP1CC/PPP2R1B/PPP2R5A/PRC1/PRKCZ/PTK2/RHPN2/SGO1/SGO2/TUBB8/ZW10 |
| R-HSA-3371453 | Regulation of HSF1-mediated heat shock response | 0.00634891515871649 | 14 | BAG3/BAG4/HSPA14/HSPA4L/NDC1/NUP205/NUP35/NUP37/NUP43/NUP54/NUP98/POM121/RPA3/TPR |
| R-HSA-111471 | Apoptotic factor-mediated response | 0.00657217993058104 | 7 | APIP/AVEN/CASP3/CDKN2A/CYCS/DIABLO/GSDMD |
| R-HSA-69481 | G2/M Checkpoints | 0.00677788452050319 | 25 | CCNB1/CCNB2/CDC25C/CDC7/CDK1/CHEK1/DBF4/DNA2/H4C3/H4C8/MCM8/MDC1/NBN/PSMA3/PSMA4/PSMB3/PSMD10/PSME2/RAD1/RAD50/RPA3/SEM1/SUMO1/UBE2V2/WEE1 |
| R-HSA-4570464 | SUMOylation of RNA binding proteins | 0.00736085788445777 | 11 | HNRNPC/NDC1/NUP205/NUP35/NUP37/NUP43/NUP54/NUP98/POM121/SUMO1/TPR |
| R-HSA-168274 | Export of Viral Ribonucleoproteins from Nucleus | 0.00737554235603299 | 9 | NDC1/NUP205/NUP35/NUP37/NUP43/NUP54/NUP98/POM121/TPR |
| R-HSA-168276 | NS1 Mediated Effects on Host Pathways | 0.00745595494311544 | 10 | KPNA2/NDC1/NUP205/NUP35/NUP37/NUP43/NUP54/NUP98/POM121/TPR |
| R-HSA-429914 | Deadenylation-dependent mRNA decay | 0.00767237381119931 | 12 | CNOT7/EIF4E/EIF4G1/EXOSC8/LSM2/LSM3/LSM4/LSM5/LSM6/PABPC1/PAIP1/TTC37 |
| R-HSA-70171 | Glycolysis | 0.00772967830815589 | 14 | GNPDA1/NDC1/NUP205/NUP35/NUP37/NUP43/NUP54/NUP98/PFKP/PGAM1/PGK1/POM121/PPP2R1B/TPR |
| R-HSA-162587 | HIV Life Cycle | 0.00828650412658972 | 23 | CHMP2B/CHMP4A/FURIN/GTF2F2/NCBP2/NDC1/NUP205/NUP35/NUP37/NUP43/NUP54/NUP98/POLR2A/POM121/PSIP1/RCC1/RNGTT/TAF5/TAF7/TCEA1/TPR/VTA1/XRCC6 |
| R-HSA-1169408 | ISG15 antiviral mechanism | 0.00871464810545135 | 14 | EIF4E/EIF4G1/FLNB/KPNA2/NDC1/NUP205/NUP35/NUP37/NUP43/NUP54/NUP98/POM121/STAT1/TPR |
