# Supplementary material for: Tumor treating fields affect mesothelioma cell proliferation by exerting histotype-dependent cell cycle checkpoint activations and transcriptional modulations
Source: Cell Death Dis. 2022 Jul 15;13(7):612. doi: 10.1038/s41419-022-05073-4 (PMC9287343; doi:10.1038/s41419-022-05073-4)

Full WB p27 – Supplementary Figure S5

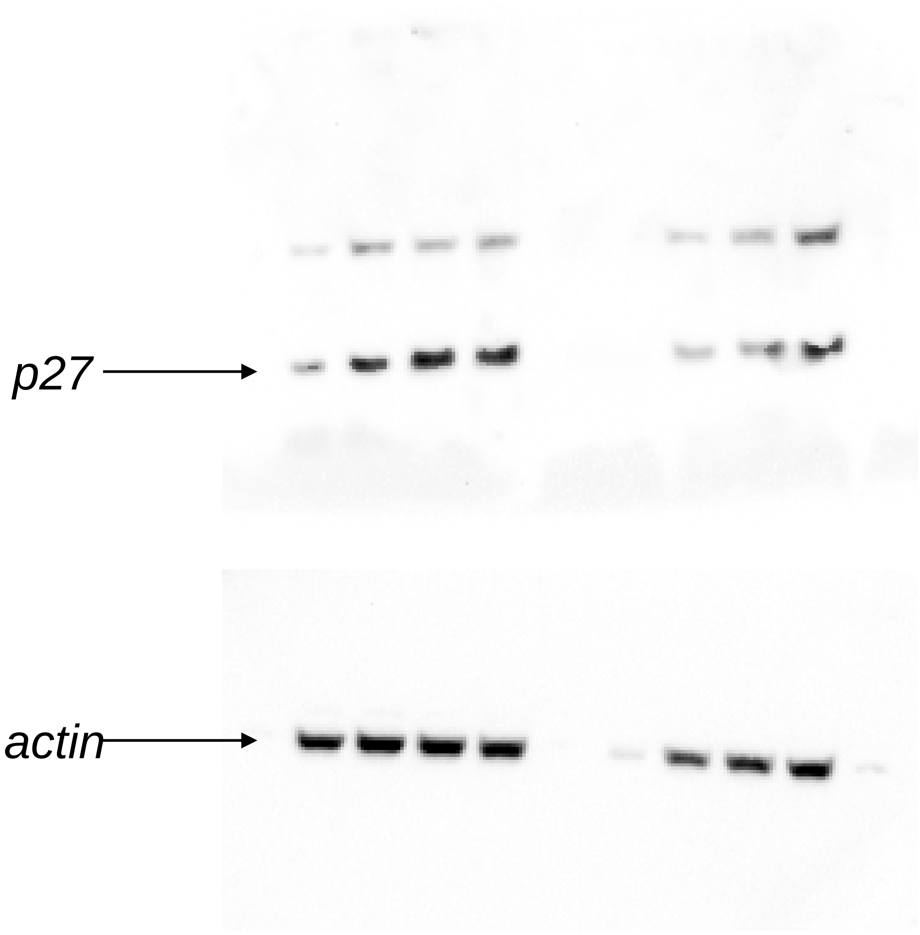

Full WB p53 – Supplementary Figure S5

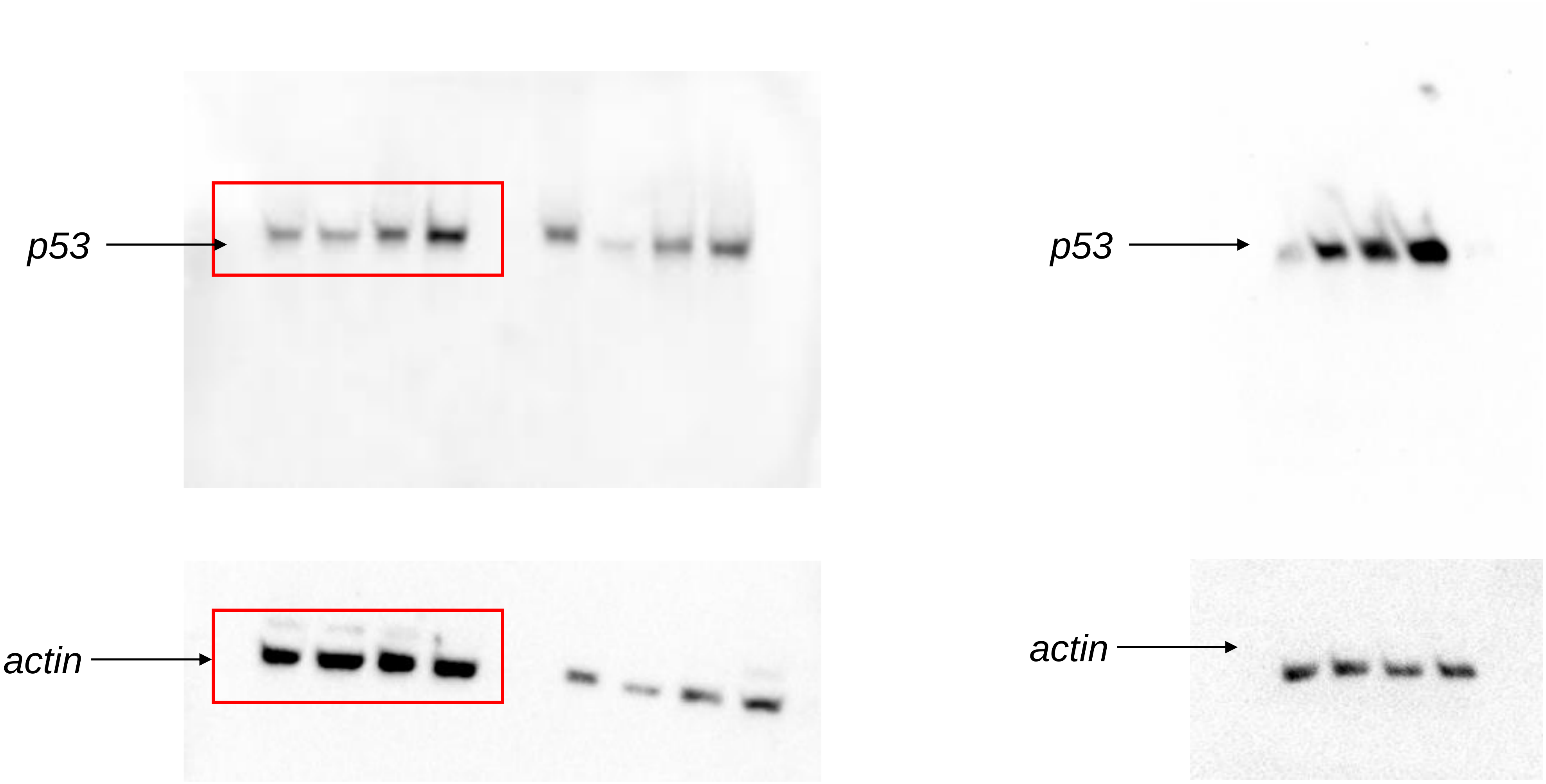

Full WB pChk1 and Chk1 – Supplementary Figure S5

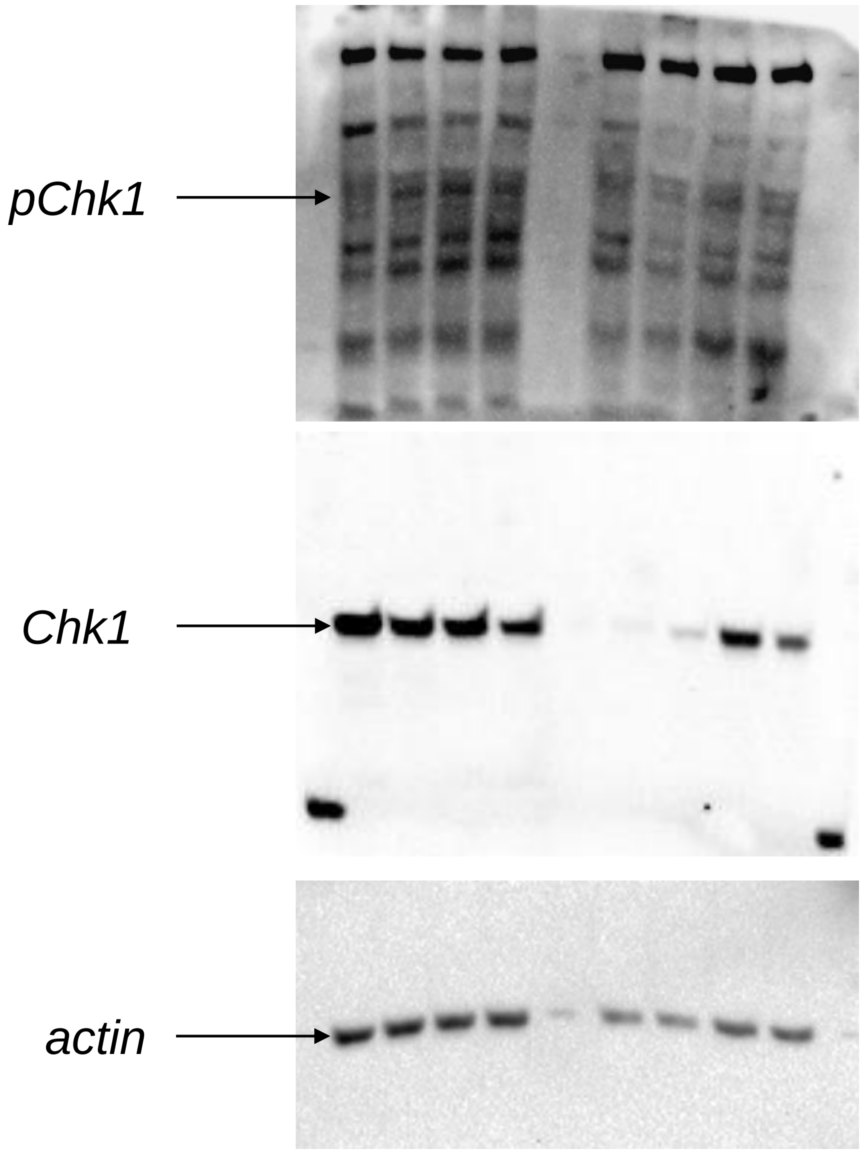

Full WB caspase-9 – Supplementary Figure S5

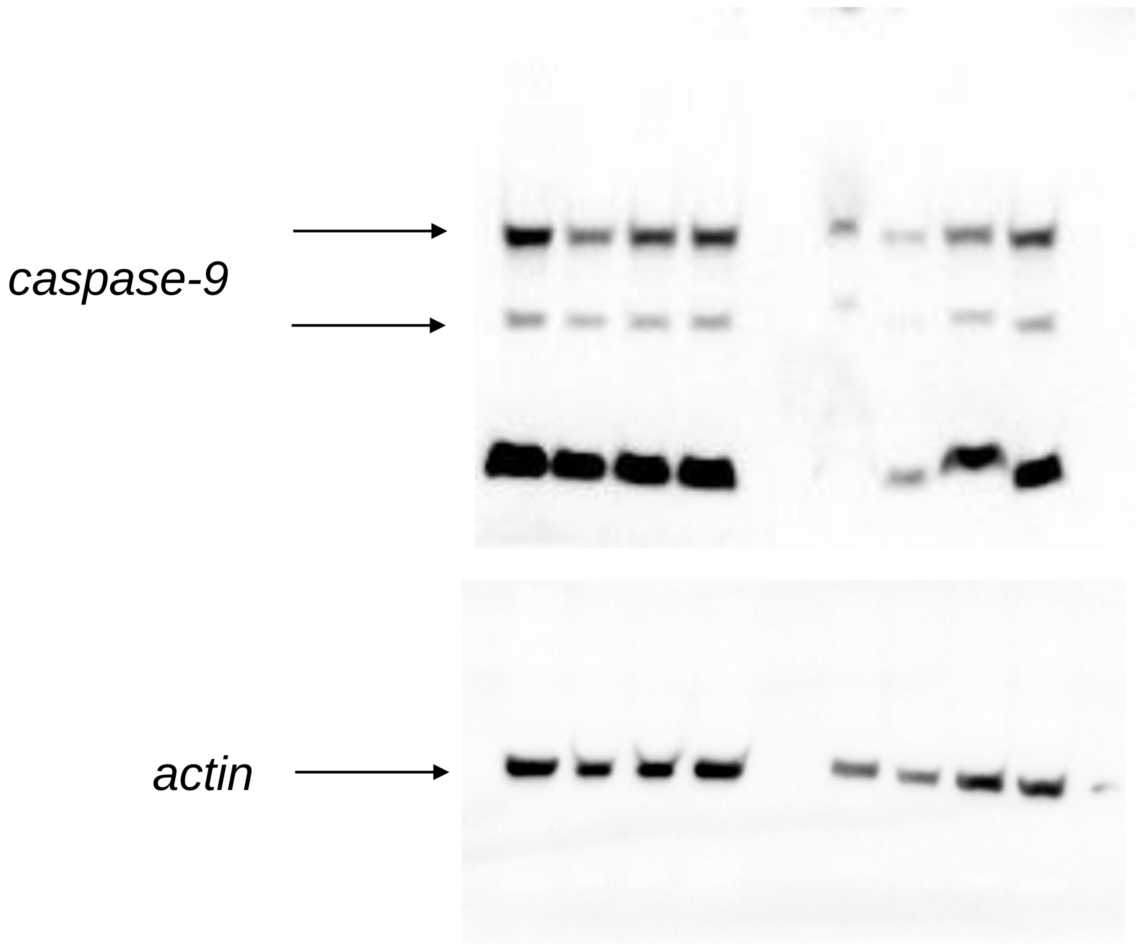

Full WB caspase-3 – Supplementary Figure S5 (in Supplementary Fig. S5 the order of CD473 and CD60 samples is inverted)

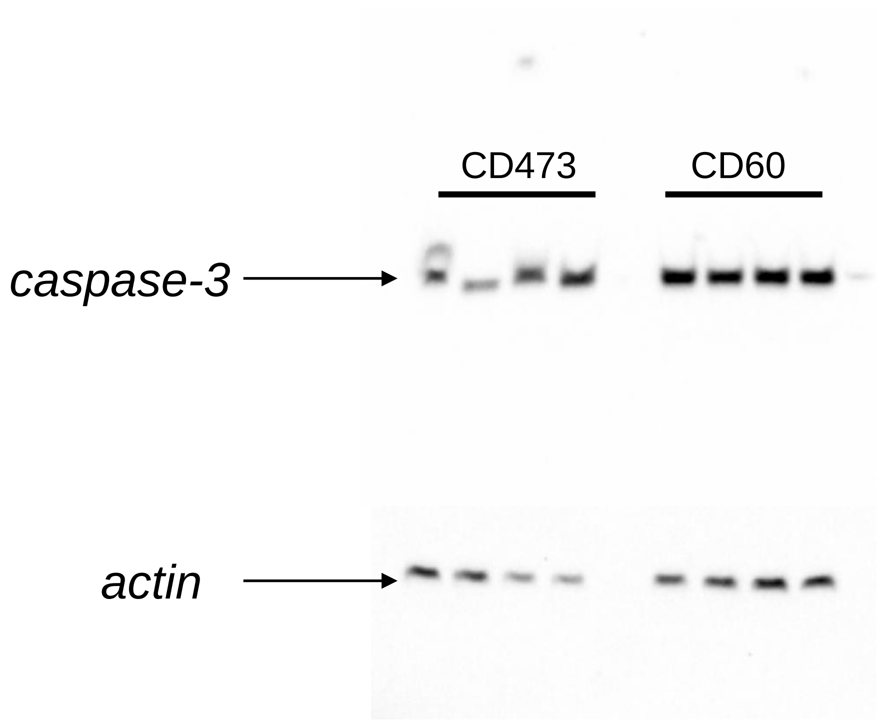

Full WB NOTUM – Supplementary Figure S7

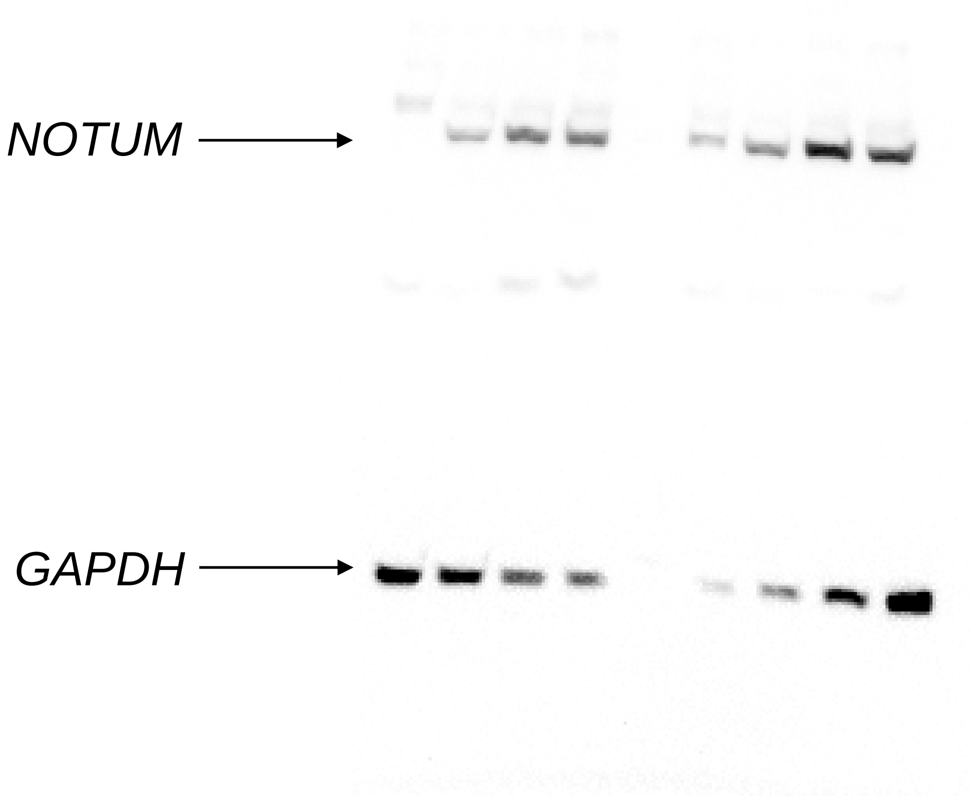

Supplement: Supplementary file 5 — Original Western Blot [file 41419_2022_5073_MOESM5_ESM.pdf]
