# Supplementary material for: Tumor treating fields affect mesothelioma cell proliferation by exerting histotype-dependent cell cycle checkpoint activations and transcriptional modulations
Source: Cell Death Dis. 2022 Jul 15;13(7):612. doi: 10.1038/s41419-022-05073-4 (PMC9287343; doi:10.1038/s41419-022-05073-4)
Supplement: Supplementary file 7 — Author contribution for [file 41419_2022_5073_MOESM7_ESM.pdf]

# DECLARATION OF CONTRIBUTIONS TO ARTICLE

ADMC

Manuscript Number: CDDIS-22-1407RR

Journal Name: *Cell Death & Disease* (the 'Journal')

Proposed Title of the Contribution:

Tumor treating fields affect mesothelioma cell proliferation by exerting histotype-dependent cell cycle checkpoint activations and transcriptional modulations (the 'Contribution)

Author(s):

Laura Mannarino, Federica Mirimao, Nicolò Panini, Lara Paracchini, Sergio Marchini, Luca Beltrame, Rosy Amodeo, Federica Grosso, Roberta Libener, Irene De Simone, Giovanni L. Ceresoli, Paolo A. Zucali, Monica Lupi and Maurizio D'Incalci (the 'Authors')

For all *CDDis* articles, each person named as an author in the published version must be able to show he or she has contributed substantially to the article.

Authorship credit should be based on 1) substantial contributions to conception and design, acquisition of data, or analysis and interpretation of data; 2) drafting the article or revising it critically for important intellectual content; and 3) final approval of the version to be published. Authors should meet conditions 1, 2 and 3.

Any person who cannot be shown to have made a substantial contribution to the article cannot be listed as an author in the final version. The name of any person who is deemed to have made a minor contribution can, however, appear in the Acknowledgments section of the article.

Please complete the table below to indicate the contributions of all named authors to the manuscript.

| Author Full Name: | Specification of Contribution to the Manuscript: |
|---|---|
| Laura Mannarino | Performed the experiments and analyzed the data |
| Federica Mirimao | Performed the experiments and analyzed the data |
| Nicolò Panini | Performed flow cytometric analyses |
| Lara Paracchini | Acquired and analyzed genomic and transcriptomic data |
| Sergio Marchini | Acquired and analyzed genomic and transcriptomic data |
| Luca Beltrame | Acquired and analyzed genomic and transcriptomic data |
| Rosy Amodeo | Contributed to the experiments and provided conceptual inputs |
| Federica Grosso | Collected patients' tissues used for transcriptomic analyses and provided conceptual inputs |
| Roberta Libener | Stabilized and provided patient-derived cell lines |
| Irene De Simone | Collected patients' tissues used for transcriptomic analyses and provided conceptual inputs |
| Giovanni Luca Ceresoli | Collected patients' tissues used for transcriptomic analyses and provided conceptual inputs |
| Paolo Andrea Zucali | Collected patients' tissues used for transcriptomic analyses and provided conceptual inputs |
| Monica Lupi and Maurizio D'Incalci | Designed the experiments and wrote the paper |

Please complete the table below to indicate the contributions of all named authors to the figures.

Figure 1:

FM generated the data; ML assembled the figure.

Figure 2:

FM generated the data of panel A; NP generated the data of panel B; ML assembled the figure.

Figure 3:

FM and NP generated the data; ML assembled the figure.

Figure 4:

FM generated the data of panel A; NP generated the data of panel B; ML assembled the figure.

Figure 5:

LP and RA generated the data; SM, LM and LB analyzed the data; LM and ML assembled the figure.

Figure 6:

LP and RA generated the data; LM and LB analyzed the data; LM and ML assembled the figure.

| Signed for and on behalf of the Author(s): | Print Name: | Date: |
|---|---|---|
| Monica Lupi | Monica Lupi | July 5th 2022 |
